# Three-dimensional models of *Mycobacterium tuberculosis* proteins Rv1555, Rv1554 and their docking analyses with sildenafil, tadalafil, vardenafil drugs, suggest interference with quinol binding likely to affect protein’s function

**DOI:** 10.1186/s12900-018-0085-4

**Published:** 2018-04-18

**Authors:** Pallabini Dash, M. Bala Divya, Lalitha Guruprasad, Kunchur Guruprasad

**Affiliations:** 1 Kunchur Guruprasad, Bioinformatics, Centre for Cellular and Molecular Biology (CCMB), Uppal Road, Hyderabad, 500007 India; 2 School of Chemistry, University of Hyderabad, Gachibowli, Hyderabad, 500046 India

**Keywords:** Tuberculosis, Predicted drug targets, Repurposed drugs, Computational biology

## Abstract

**Background:**

Earlier based on bioinformatics analyses, we had predicted the *Mycobacterium tuberculosis* (*M.tb*) proteins; Rv1555 and Rv1554, among the potential new tuberculosis drug targets. According to the ‘TB-drugome’ the Rv1555 protein is ‘druggable’ with sildenafil (Viagra), tadalafil (Cialis) and vardenafil (Levitra) drugs. In the present work, we intended to understand via computer modeling studies, how the above drugs are likely to inhibit the *M.tb* protein’s function.

**Results:**

The three-dimensional computer models for *M.tb* proteins; Rv1555 and Rv1554 constructed on the template of equivalent membrane anchor subunits of the homologous *E.coli* quinol fumarate reductase respiratory protein complex, followed by drug docking analyses, suggested that the binding of above drugs interferes with quinol binding sites. Also, we experimentally observed the in-vitro growth inhibition of *E.coli* bacteria containing the homologous *M.tb* protein sequences with sildenafil and tadalafil drugs.

**Conclusions:**

The predicted binding sites of the drugs is likely to affect the above *M.tb* proteins function as quinol binding is known to be essential for electron transfer function during anaerobic respiration in the homologous *E.coli* protein complex. Therefore, sildenafil and related drugs currently used in the treatment of male erectile dysfunction targeting the human phosphodiesterase 5 enzyme may be evaluated for their plausible role as repurposed drugs to treat human tuberculosis.

**Electronic supplementary material:**

The online version of this article (10.1186/s12900-018-0085-4) contains supplementary material, which is available to authorized users.

## Background

Tuberculosis (TB) is caused by the bacillus *M.tb* and is one of the major infectious diseases affecting human beings. The current standard therapeutic treatment makes use of a combination of four main drugs; rifampicin, isoniazid, pyrazinamide, ethambutol given to a patient for a period of four to 6 months. However, with the emergence of drug-resistance, particularly, for unresponsive patients with multi-drug resistance TB (MDR-TB) and extensively drug-resistant TB (XDR-TB), there is an urgent need to identify new targets and drugs to treat tuberculosis in humans. In this context, based on our previous bioinformatics analyses, we had predicted the *M.tb* Rv1555 protein as a potential new TB drug target [1]. The *M. tuberculosis* Rv1555 protein is homologous to the D-chain monomer in the crystal structure of *Escherichia coli* quinol-fumarate reductase (QFR) respiratory protein complex [2, 3]. According to the ‘TB-drugome’ [4], this protein is predicted to bind the drugs; sildenafil (Viagra), tadalafil (Cialis) and vardenafil (Levitra). However, the binding site and mode of binding that is likely to inhibit the protein’s function is not known. These drugs are currently used in the treatment of male erectile dysfunction and are known inhibitors of the human phosphodiesterase 5 (PDE5) enzyme. The crystal structures of human PDE5 enzyme bound to the above drugs are available in the Protein Data Bank (PDB) [5]. The PDB codes corresponding to sildenafil, tadalafil and vardenafil bound to the catalytic domain of human PDE5 are; 1UDT, 1UDU and 1UHO, respectively [6]. Another crystal structure for human PDE5 complexed with sildenafil is also available in the PDB and it defines the region Tyr664-Tyr676 and orientation of the methylpiperazine moiety in sildenafil (PDB code: 2H42) [7].

In the present study, using computer modeling and docking analyses, we suggest how the binding of sildenafil, tadalafil and vardenafil drugs to the *M.tb* proteins Rv1555 and Rv1554 is likely to inhibit their function. The amino acid residues predicted to interact with above drugs in the *M.tb* proteins serve as useful candidates for mutation and biochemical validation studies. In this study, we also experimentally demonstrate the in-vitro inhibition of *E.coli* bacterial growth with sildenafil and tadalafil drugs.

## Methods

### Three dimensional modelling

Computer models corresponding to the three-dimensional structure of the *M.tb* proteins were constructed using MODELER software [8]. MODELER aligns the query protein sequence to sequence(s) of template and constructs a model based on the satisfaction of spatial restraints extracted from the template. The template represents homologous proteins of known three-dimensional structure selected from the PDB using the BLAST program [9]. The template for the *M.tb* Rv1555 protein corresponds to the homologous D-chain of the *E.coli* QFR respiratory protein complex (PDB code:1KF6) [3]. The *M.tb* dimer model represents two protein chains corresponding to Rv1555 protein and its membrane associated subunit protein Rv1554 as in the equivalent *E.coli* QFR membrane anchor subunits. The *M.tb* Rv1554 protein was identified using the C-chain of *E.coli* QFR respiratory protein complex (PDB code:1KF6_C-chain) as query sequence in the BLAST program [9] and by searching the database of *M.tb* protein sequences in H37Rv genome available at NCBI. The dimer model was generated by aligning the two *M.tb* sequences to their corresponding *E.coli* QFR sequences in MODELER and by the extraction and satisfaction of spatial restraints in the dimer template. The CLUSTAL program [10] was used for comparing the *E.coli* QFR and *M.tb* protein sequences. The models were evaluated using the Ramachandran plot available in the PROCHECK software [11].

### Drug docking

The binding sites and poses for sildenafil, tadalafil and vardenafil drugs to the *M.tb* proteins, were predicted using the Genetic Optimisation for Ligand Docking (GOLD) software [12]. GOLD uses the genetic algorithm for docking flexible ligands into protein binding sites in order to predict the bound conformations of small chemical compounds/ligands to protein target of known three-dimensional structure. The Hermes (1.8.0 version) visualizer component in GOLD (5.4.0 version) was used for preparing the input files for docking and GoldMine (1.7.0 version) was used for analyses of the results. The Wizard feature in GOLD was used to guide the docking procedure. The coordinates of *M.tb* Rv1555 protein was loaded using Hermes and hydrogen atoms were added to the protein to define the ionisation and tautomeric states unambiguously. In order to predict the drug binding sites, the ‘cavity_atoms’ file in GOLD was defined by specifying all residues in the protein chain. The detection of cavity was restricted to solvent-accessible surface and all hydrogen bond donor/acceptors were forced to be treated as solvent accessible. The Cartesian coordinates for sildenafil, tadalafil, vardenafil drugs were obtained from the corresponding crystal structure complexes of these drugs to the human PDE5 [6, 7] protein (PDB codes; 1UDT, 1UDU, 1UHO, respectively). These inhibitors were protonated with hydrogen atoms and the files were saved in the .mol2 format for use in GOLD. The number of conformations to be generated for each inhibitor was set to 10, by allowing for early termination when the best three solutions were within 1.5 Å of each other. The ‘internal ligand energy offset’ was selected and the Piecewise Linear Potential function (CHEMPLP) was chosen as the fitness function for the Genetic Algorithm (GA). The slow (most accurate) option for performing the GA search was selected with the default parameter file set to ‘auto’ mode for optimization of the fitness score. The GOLD default parameters were as follows: population size;100, selection pressure;1.1, number of operations;100,000, number of islands;5, niche size;2, crossover frequency;95, mutation frequency;95, migration frequency;10. The resulting conformation and corresponding rank (.rnk) files were arranged according to the GoldScore and saved under the ‘output options’. The GOLD program was executed in the interactive mode and the results of docking were examined using ‘view solutions’. The best conformation according to the GoldScore was selected for representing the predicted binding site and pose. The docking of each ligand to the protein target was repeated in order to ensure reproducibility of the binding site and pose corresponding to the best docked ligand conformation in each case. All ligands were docked to the target protein using the same procedure. The docking of ligands to the dimer comprising Rv1555 and Rv1554 proteins was carried out in the same manner. In this case, the coordinates of the *M.tb* dimer model was used and the ‘cavity_atoms’ file was defined by including all residues of the dimer to constitute the binding site. Further, in order to evaluate the binding site and poses of the drugs to the *M.tb* proteins when structural flexibility is allowed, we once again repeated the docking analyses using the GOLD software. One set of docking analyses with the drugs was carried out with the proteins considered flexible and another with both the protein and the drugs considered flexible. The above analyses was carried out for the monomer and dimer *M.tb* protein models. The PyMol software [13] was used to compare the results of docking for the rigid and flexible proteins, visualize the drug binding site, drug binding mode, infer drug-protein interactions and to generate figures.

### *E.coli* growth inhibition studies with sildenafil and tadalafil drugs

Two drug molecules, sildenafil and tadalafil obtained as a gift from a local company were used for the inhibition studies. Various concentrations corresponding to 5 μg/μL, 10 μg/μL, 20 μg/μL 30 μg/μL, 40 μg/μL and 50 μg/μL of sildenafil and tadalafil were prepared in dimethyl sulfoxide (DMSO). *E. coli* cells were grown in fresh Luria-Bertani (LB) medium at 37 °C and these cells were used for plating. 100 μL of fresh *E. coli* suspension was streaked on the plain agar plate and was left to dry for 5 min. About 6 mm sterilized paper discs were placed on the agar plate by gently pressing them. 40 μL of various concentrations of both drugs were added to the paper discs. The solvent DMSO was used as a control. The plates were inverted and incubated for 18 h at 37 °C. All experiments were done under aseptic condition.

## Results

### Three-dimensional models

**Fig. 1:**
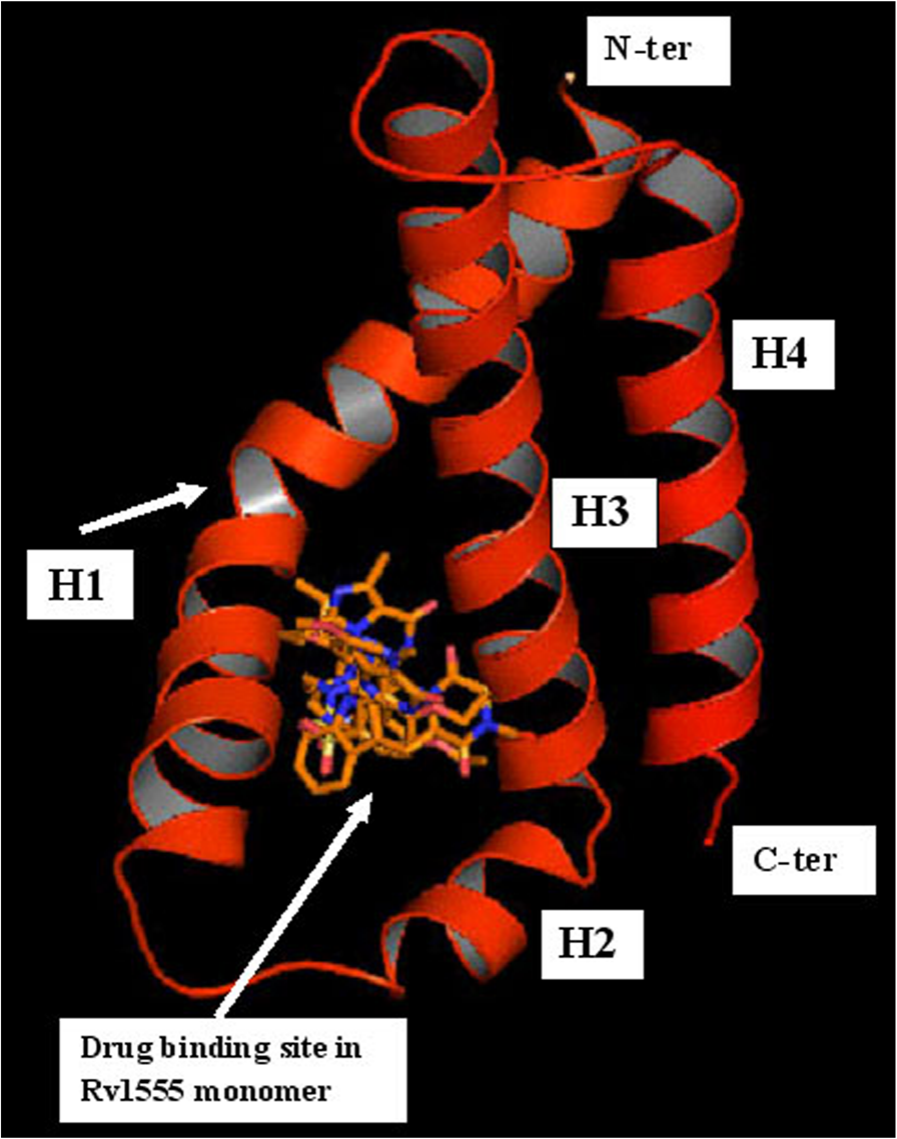
Sildenafil (Viagra), tadalafil (Cialis), vardenafil (Levitra) drugs bound to *M.tb* Rv1555 protein. The helices are labelled and the ‘kink’ in H1 helix is indicated with an arrow

The *M. tuberculosis* Rv1555 protein shares ~ 43% sequence identity to its homologous *E.coli* quinol-fumarate reductase subunit sequence (PDB code:1KF6_D-chain) used as template for modeling. According to the Ramachandran plot available in PROCHECK software, 87.9% residues were observed in the ‘allowed region’, 12.1% in the ‘additionally allowed regions’ and none of the residues were observed in the ‘disallowed region’, suggesting the good stereo-chemical quality of the model. The root mean square deviation value for the model compared to the template was 0.49Ǻ (for alpha-carbon atoms) and 0.89Ǻ (for all atoms included) as expected for good quality models. The predicted fold corresponding to the *M.tb* Rv1555 protein mainly comprised four helices (labeled H1 to H4) as shown in Fig. 1. The H1 helix near the N-terminus was ‘kinked’ and the H2 helix was relatively short compared to the other helices. The dimer *M.tb* model comprised Rv1555 and Rv1554 proteins. The *M.tb* Rv1554 protein predicted as the equivalent of the *E.coli frdC* gene (PDB code:1KF6_C-chain) had a BLAST score 58.2 and e-value 1e-12. More than 95% query sequence had 31% sequence identity. The Ramachandran plot corresponding to the dimer model contained more than 90% residues in the ‘most favoured region’ suggesting good quality of the model. The Rv1554 protein in the dimer also primarily comprised four helices (labeled H1’ to H4’) and represents the relative orientation of the individual membrane subunit proteins. The H1’ helix in Rv1554 is not ‘kinked’ unlike the H1 helix in Rv1555. In Rv1555, there is a proline residue at position 991 at the site of the ‘kink’, whereas there is no proline in the H1’ helix in Rv1554. The *M.tb* dimer model had a lower root mean square deviation value 0.37Ǻ compared to the monomer model.

### Docking sildenafil, tadalafil, vardenafil drugs in *M.tb* Rv1555 protein

The predicted models for sildenafil, tadalafil and vardenafil bound to the *M.tb* Rv1555 protein are shown in Fig. 1. All three drugs bind in a ‘cleft’ formed between the helices; H1 to H3 suggesting the above drugs can be accommodated in the *M.tb* Rv1555 protein and in support of predictions from the TB-drugome [4]. The binding of all three drugs at the same site with different binding poses is a feature that is common to the human PDE5 (PDB codes; 1UDT, 1UDU, 1UHO) and above *M.tb* proteins.

### How is the binding of sildenafil, tadalafil and vardenafil drugs to the *M.tb* Rv1555 protein likely to inhibit its function?

**Fig. 2:**
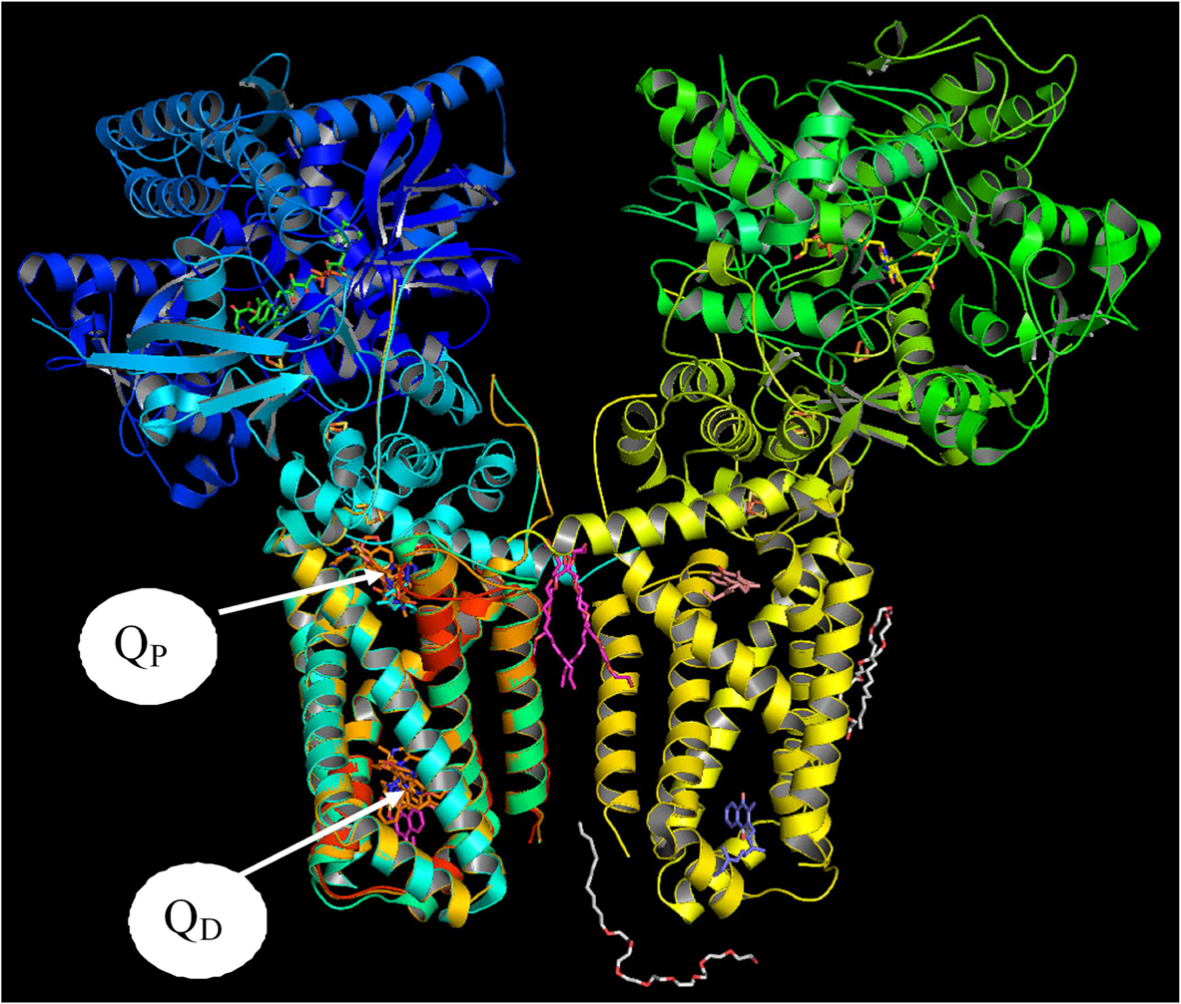
Structural overlay of the *M.tb* monomer (Rv1555) and *M.tb* dimer (Rv1555, Rv1554) proteins complexed with sildenafil, tadalafil, vardenafil drugs on to the crystal structure of *E. coli* QFR tetrameric dimer protein complex (PDB code: 1L0V). The drug binding sites coincide with the equivalent menaquinol binding at the Q_D_ site for monomer and at the Q_P_ site for dimer *M.tb* proteins

**Fig. 3:**
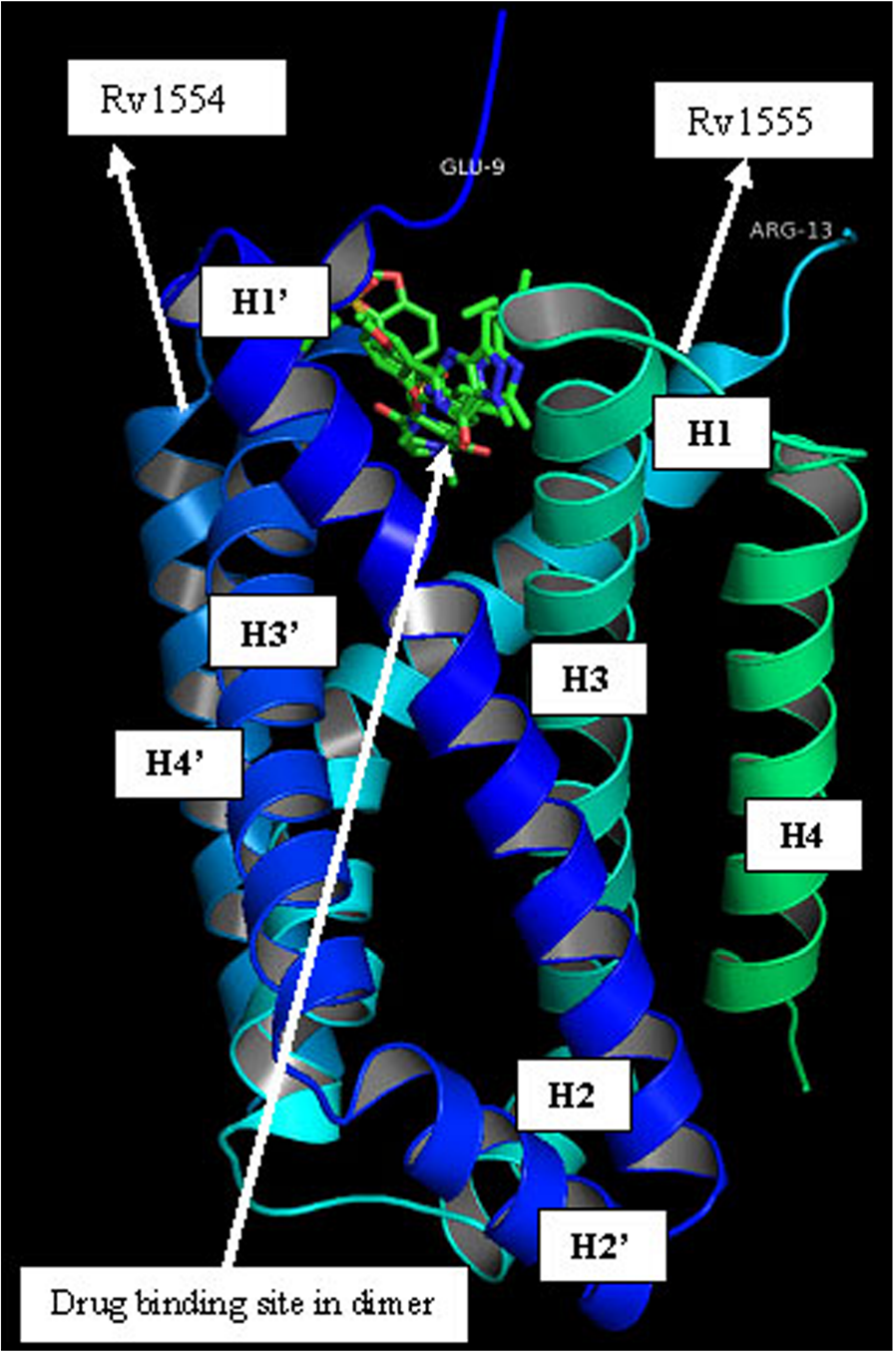
Sildenafil, tadalafil, vardenafil drugs bound to the *M.tb* dimer proteins (Rv1555 and Rv1554)

In order to address the above, we examined the homolog of *M.tb* Rv1555 protein in the crystal structure complex corresponding to the *E.coli* protein that was used as template for modeling the *M.tb* protein. When our models of the *M.tb* Rv1555 protein with bound sildenafil, tadalafil and vardenafil drugs were superimposed on to the crystal structure complex of the *E.coli* fumarate-reductase QFR protein (PDB code:1L0V), the binding site for the drugs were observed to coincide with the Q_D_ menaquinol binding site in the *E.coli* protein as shown in Fig. 2. Whereas, when our dimer *M.tb* model comprising Rv1555 and Rv1554 proteins was superimposed onto the crystal structure complex of the *E.coli* fumarate-reductase QFR protein, the binding site for the drugs, interestingly, was observed to coincide with the Q_P_ menaquinol binding site in the *E.coli* protein as shown in Fig. 3. The Q_P_ binding site corresponds to a ‘cavity’ between the H1, H3, H1’ and H3’ helices towards the other end of the Q_D_ binding site. The amino acid residues comprising the binding site pockets defined by interactions with sildenafil, tadalafil and vardenafil drugs in the crystal structure complexes of human PDE5 and modeled drug-*M.tb* protein complexes are shown in Table 1. The numbering of residues for human PDE5 is according to the crystal structure (PDB code:1UDT), whereas, the numbering for the *M.tb* models is in the sequential order. It is observed that the predicted binding sites for the drugs; sildenafil, tadalafil and vardenafil drugs in *M.tb* Rv1555 protein is different in the context of the monomer and dimer models suggesting the existence of more than one binding site. Both the binding site pockets are defined for the *M.tb* proteins, i.e., Q_P_ site as in the case of dimer and Q_D_ site as in case of the monomer. The Q_D_ binding site mainly comprises hydrophobic residues, whereas, the Q_P_ site contains certain amino acid residues involved in hydrogen bond formation with the drugs.

**Table 1:** Specificity pockets defined by interactions with sildenafil (Viagra), tadalafil (Cialis), vardenafil (Levitra) drugs in three-dimensional computer models of the *M.tb* dimer, *M.tb* monomer and crystal structure complexes of human phosphodiesterase 5 enzyme

Protein	Sildenafil (Viagra)	Tadalafil (Cialis)	Vardenafil (Levitra)
*M.tb* dimer model (Rv1554)	**Phe22, Arg25**, **Glu26**, **Trp83**, **Ser86**, Ala87, **Arg89**, Ala90	**Phe22**, **Arg25**, **Glu26**, **Trp83**, **Ser86**, Ala87, **Arg89**, Ala90	Ser18, Arg21, **Phe22**, **Arg25**, **Glu26**, **Trp83**, **Ser86**, **Arg89**
(Rv1555) Q_P_ site	**Trp20**, **Phe23**, Ser24, **His84**, **Arg85**, **Phe88**	**Trp20**, **Phe23**, Ser24, **His84**, **Arg85**, **Phe88**	**Trp20**, **Phe23**, **His84**, **Arg85**, **Phe88**
*M.tb* monomer model (Rv1555) Q_D_ site	Val33, **Val36, Leu37**, **Leu40, Phe41, Leu58**, Val70, **Val71, Leu74**, **Met121**	**Val36, Leu37**, Leu38, **Leu40**, **Phe41, Leu58**, Val62, **Val71, Leu74,** Val75, **Met121**	Val33, **Val36, Leu37**, Leu38, **Leu40**, **Phe41, Leu58,** Val70, **Val71, Leu74**, Val75, **Met121**
Crystal structures of human PDE5 -drug complexes	(1UDT– VIA) **Tyr612,**His613,Asn661, Tyr664,Ile768,Ala779, Val782,**Ala783**,**Leu804, Met816,Gln817,** Gly819**, Phe820**	(1UDU– CIA) **Tyr612**,Ser663,Val782,**Ala783**, Phe786,Phe787, **Leu804,**Ile813**, Met816, Gln817, Phe820**	(1UHO– VDN) **Tyr612**,His613,Tyr664, Ala779, **Ala783**,Phe786, **Leu804, Met816, Gln817,** Gly819**, Phe820**

Note: Amino acid residues common to all three drug binding pockets are shown in bold. The residue numbering is in sequential order for *M.tb* proteins and according to numbering in crystal structure complex for human PDE5 protein as in PDB codes:1UDT, 1UDU, 1UHO for sildenafil, tadalafil, vardenafil, respectively

**Fig. 4:**
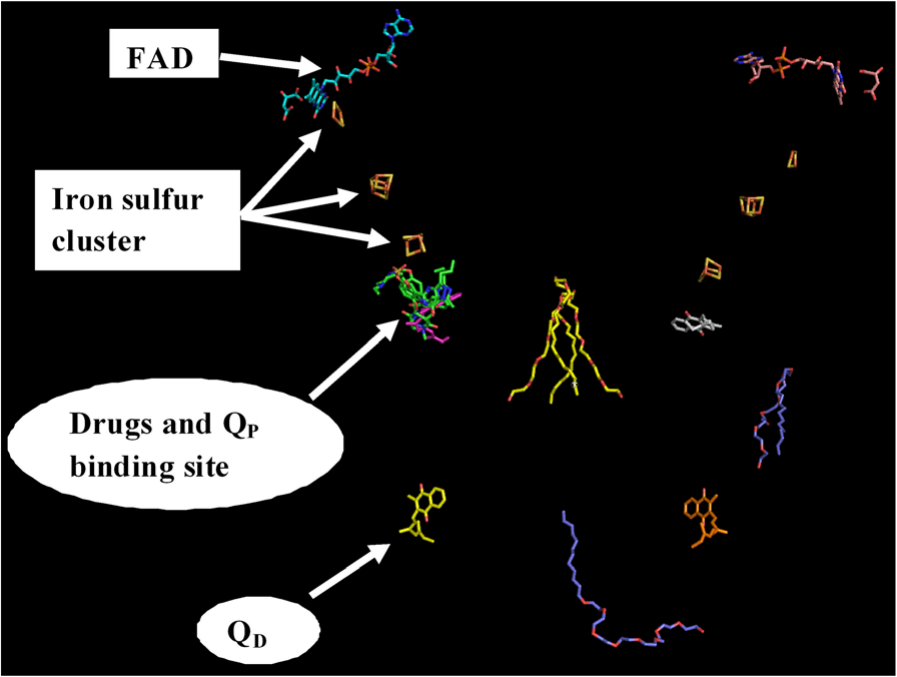
The FAD, iron-sulfur clusters, menaquinol molecules bound near the Q_P_ and Q_D_ sites in the crystal structure of *E.coli* QFR tetrameric dimer protein complex (PDB code: 1L0V) (protein is excluded for the sake of clarity). Sildenafil, tadalafil, vardenafil drug binding coinciding with the Q_P_ site in the *M.tb* dimer protein

**Fig. 5:**
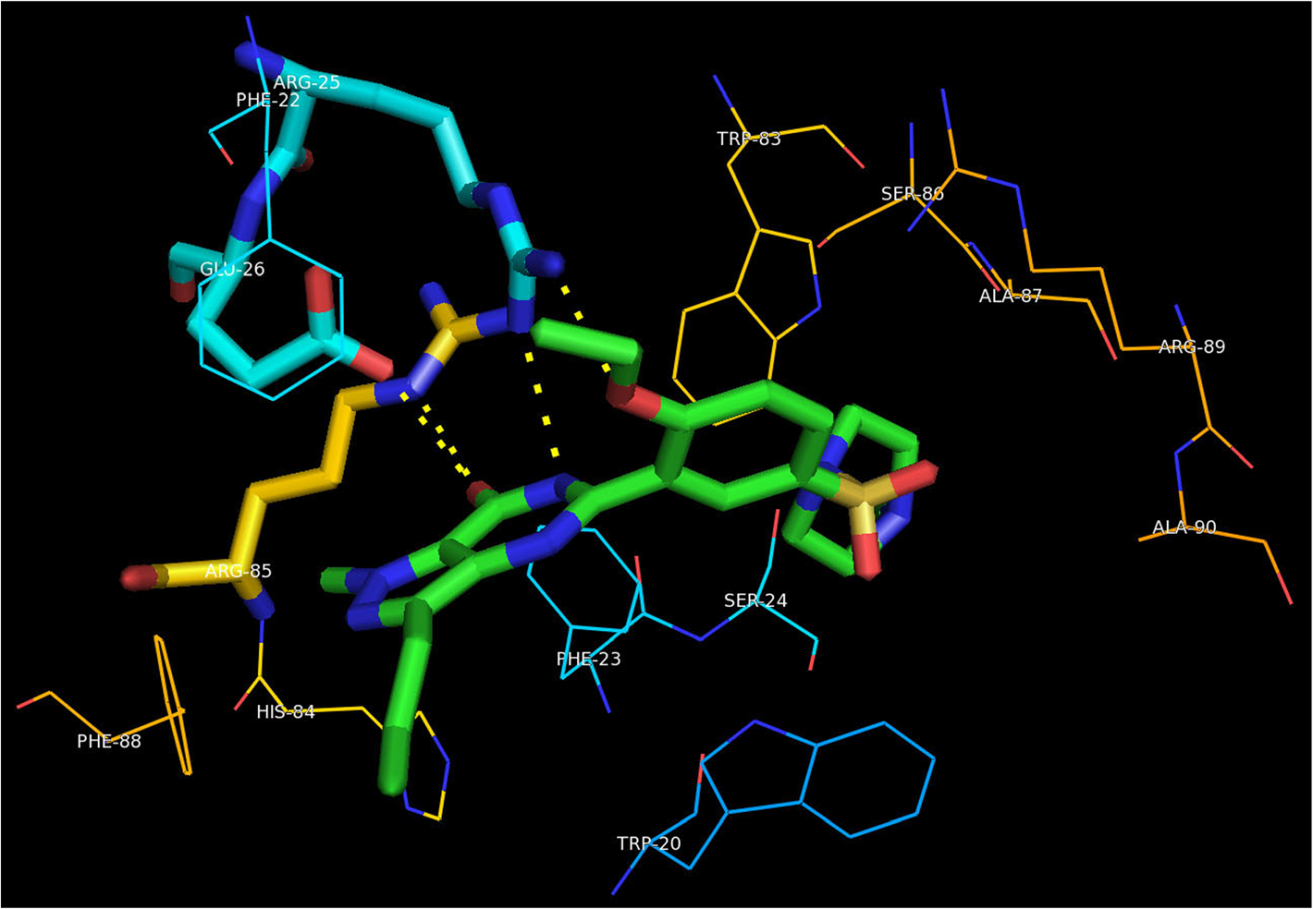
Specificity pockets and hydrogen bonds defined by interactions with sildenafil (Viagra) in the *M.tb* dimer

**Fig. 6:**
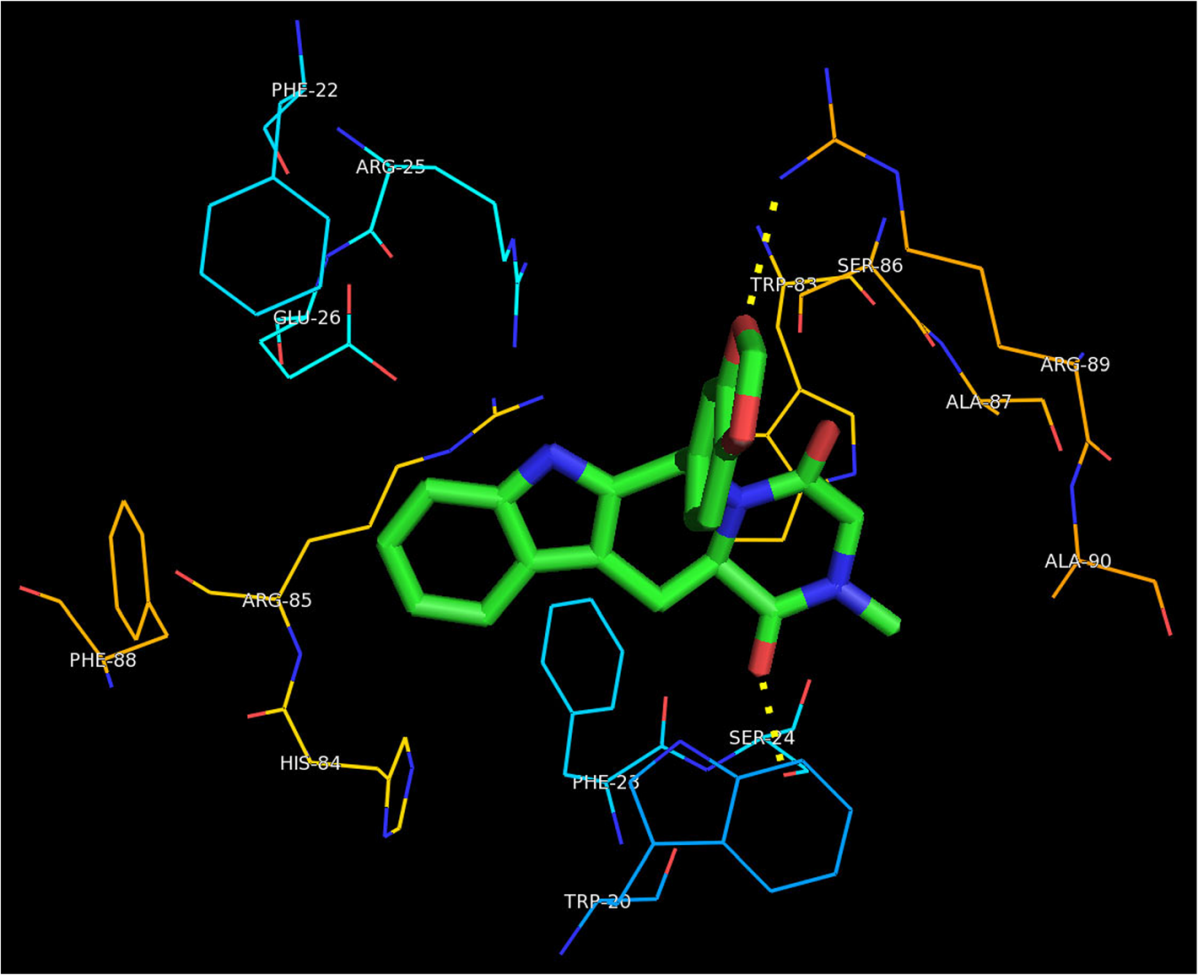
Specificity pockets and hydrogen bonds defined by interactions with tadalafil (Cialis) in the *M.tb* dimer

**Fig. 7:**
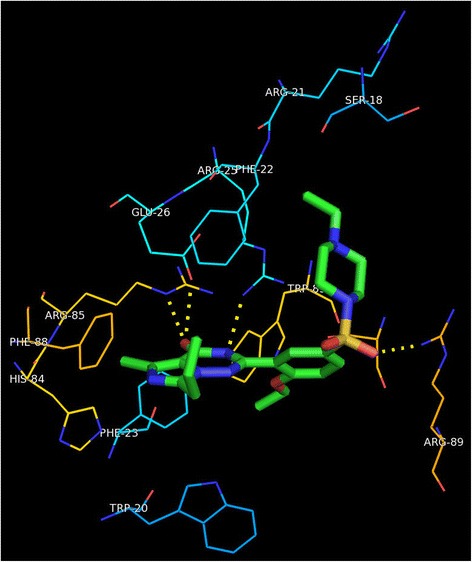
Specificity pockets and hydrogen bonds defined by interactions with vardenafil (Levitra) in the *M.tb* dimer

In Fig. 4, we show the results of the superposition of the crystal structure of *E.coli* QFR protein onto the dimer model of the *M.tb* Rv1555 complexed with the sildenafil, tadalafil and vardenafil drugs. For the sake of clarity, the proteins are excluded from the figure and only the flavin adenine dinucleotide, the iron sulfur clusters, menaquinols bound at the Q_P_/Q_D_ sites in the crystal structure of the *E.coli* QFR protein and the drugs bound near the equivalent Q_P_ site in the *M.tb* Rv1555 protein are shown. In the dimer model, hydrogen bond interactions were observed between all three drugs and the *M.tb* proteins Rv1555 and Rv1554 as shown in Table 2. The side-chain of Arg85 in Rv1555 protein, and the side-chains of Arg25 and Glu26 in Rv1554 protein are predicted to be involved in hydrogen bond interactions with sildenafil as shown in Fig. 5. The side-chain of Ser24 in Rv1555 and side-chain of Arg89 in Rv1554 are predicted to make hydrogen bond interactions with tadalafil as shown in Fig. 6. The side-chain of Arg85 in Rv1555 protein and side-chains of Arg25, Glu26, Arg89 in Rv1554 in the dimer are predicted to be involved in hydrogen bond interactions with vardenafil as shown in Fig. 7. The coordinates of the models of the *M.tb* dimer complexes with sildenafil, tadalafil and vardenafil drugs in the PDB format are attached as ‘Additional files’ in the supplementary material and are labeled Additional file 1, Additional file 2 and Additional file 3, respectively. Our analyses suggests that the binding of sildenafil, tadalafil, vardenafil drugs to the *M.tb* Rv1555 and Rv1554 proteins interferes with equivalent quinol binding sites observed in the homologous membrane anchor subunits of the *E.coli* QFR protein.

**Table 2:** Drug-protein hydrogen bond interactions in human PDE5 and *M.tb* proteins

Drug	Drug atom labels	Human PDE5 residues (atom label)	Drug atom labels	*M.tb* dimer protein residues (atom label)	*M.tb* dimer proteins (Rv1555/Rv1554)
Sildenafil (Viagra)	N22 O27 N29	Gln817 (OE1) Gln817 (NE2) (solvent mediated interactions with Asp764 main and side chain, Ala767 main chain & Tyr612 side chain)	O27 N22 O3 O27	Arg85 (NE) Arg25 (NH1) Arg25(NH2) Glu26 (OE2)	Rv1555 Rv1554 Rv1554 Rv1554
Tadalafil (Cialis)	N9	Gln817 (OE1)	O28 O32	Arg89 (NH1) Ser24 (OG)	Rv1554 Rv1555
Vardenafil (Levitra)	N22 O27 O12 (in SO2)	Gln817 (OE1) Gln817 (NE2) (solvent mediated interactions with Ser663 main chain)	O27 O27 N22 O12 (in SO2)	Arg85 (NE) Glu26 (OE2) Arg25 (NH1) Arg89 (NH1)	Rv1555 Rv1554 Rv1554 Rv1554

### Sequence alignment corresponding to the *M.tb* and *E.coli* membrane anchor subunits

**Fig. 8:**
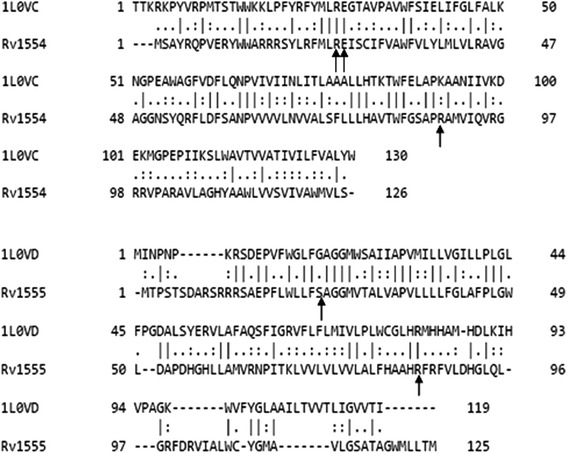
Multiple sequence alignment for the *M.tb* Rv1555 protein and its associated membrane subunit protein (Rv1554) in the dimer, along with their corresponding homologous sequences in the *E.coli* quinol-fumarate reductase membrane anchor subunit dimer protein (as in PDB code:1L0V). The residues are labelled sequentially for the individual subunits in the proteins. Residues involved in the drug-protein side-chain hydrogen bond interactions are indicated by arrows

The multiple sequence alignment corresponding to the equivalent membrane anchor subunit sequences in the *E.coli* QFR proteins (1L0V_C-chain, 1L0V_D-chain) and the *M.tb* proteins (Rv1555, Rv1554) are shown in Fig. 8. The glutamic acid residue Glu29 corresponding to the FrdC chain in *E. coli* QFR protein (PDB code:1L0V_C-chain), which is suggested to act as a proton shuttle from the quinol during enzyme turnover [3] is also conserved in the *M.tb* Rv1554 protein (Glu26 in the alignment), suggesting a similar action in the equivalent *M.tb* protein. Further, Arg85 in Rv1555 protein predicted to make side-chain hydrogen bond interactions with sildenafil, tadalafil is conserved in *E.coli* and *M.tb* proteins. Also, Arg25 in Rv1554 protein predicted to be involved in side-chain hydrogen bond interactions with sildenafil and vardenafil drugs is conserved in the alignment comprising the *E.coli* and *M.tb* protein sequences.

### Flexibile-protein flexible-drug docking

**Fig. 9:**
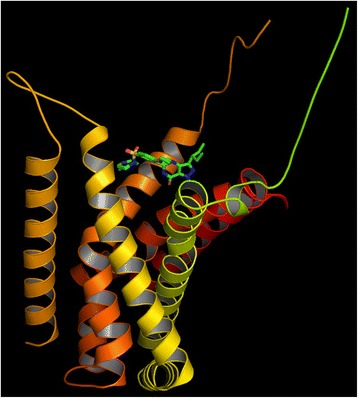
Structural overlay showing the binding of sildenafil drug to the *M.tb* rigid and flexible dimer protein complexes

In the case of flexible-protein flexible-drug docking too, all the three drugs bind near equivalent Q_P_ and Q_D_ sites in the case of dimer and monomer, respectively. Particularly, the binding with sildenafil in the dimer complex was almost identical to that observed in the case of rigid protein docking complex as shown in Fig. 9. The side-chains of Arg25, Glu26 and Arg85 were involved in hydrogen bond interactions with sildenafil suggesting the drug can bind to the *M.tb* protein even when the protein and drug are treated as flexible. Certain differences were observed with respect to vardenafil and tadalafil binding in the case of the flexible protein complex relative to the rigid protein complex. In case of vardenafil, the drug binding site was the same, except the orientation of drug was different. However, the hydrogen bond interactions involving the side-chain of Arg25 with vardenafil were present in the rigid as well as the flexible protein complexes. In case of tadalafil too, the drug binding site was the same, except that there was a relative shift in the position of the bound drug. This shift favours the formation of additional hydrogen bonds via interactions with side-chains of Arg25, Ser86 and Arg89 in the flexible protein complex. The Arg89 side-chain hydrogen bond interaction with tadalafil was common to the rigid and flexible drug-protein complexes.

### Inhibition of *E.coli* bacterial growth in the presence of sildenafil and tadalafil

**Fig. 10:**
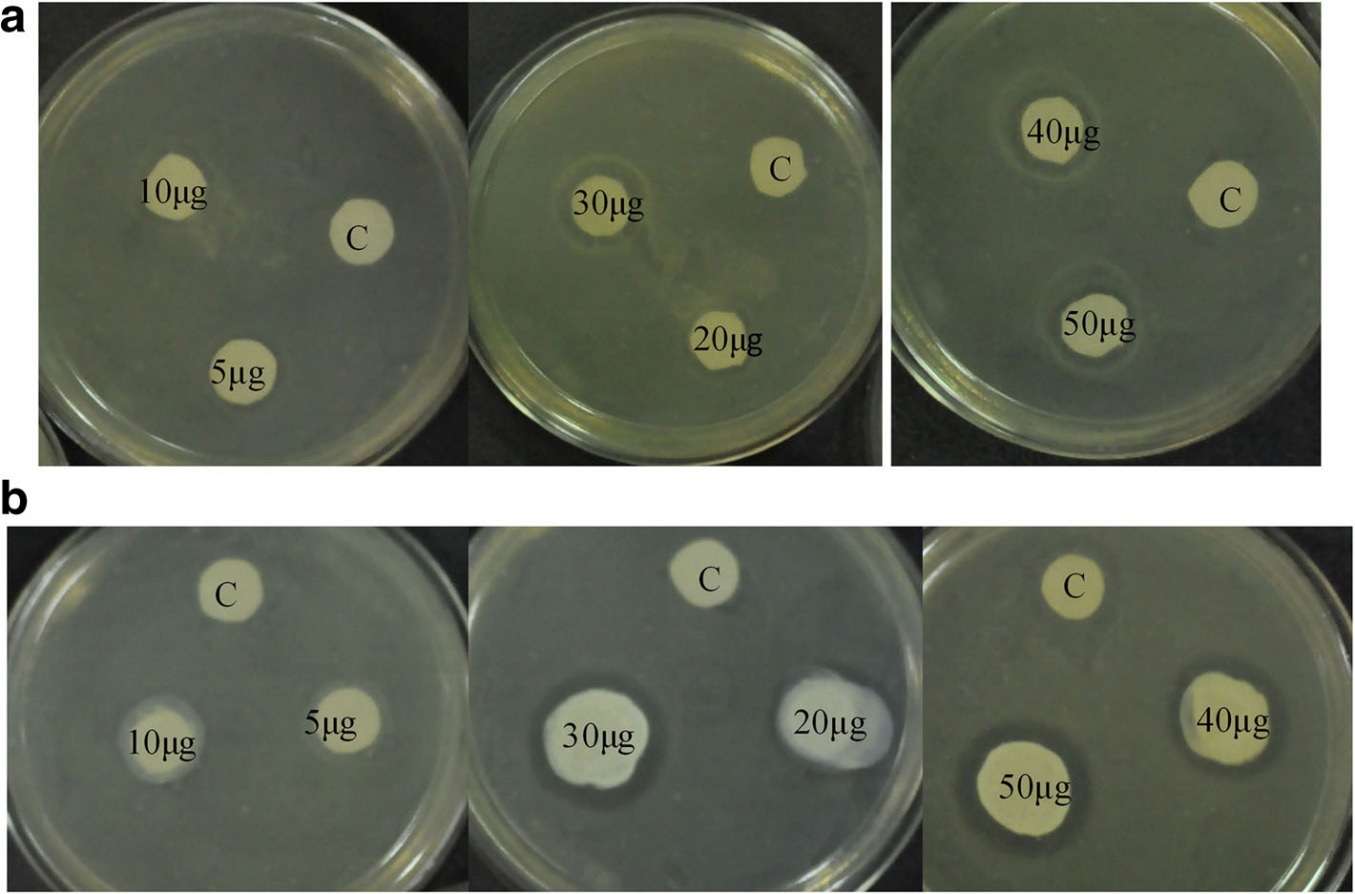
Antibacterial activity of (A) Sildenafil, (B) Tadalafil. 40 μL of 5 μg/μL, 10 μg/μL, 20 μg/μL, 30 μg/μL, 40 μg/μl, 50 μg/μL and control (C) added to the paper disc

In order to investigate the effect of sildenafil and tadalafil on the growth of *E. coli* cells, we carried out experiments using the agar disc diffusion method [14, 15]. Based on visual inspection, as shown in Fig. 10, we observed that DMSO (control) allows the growth *of E. coli*, while increasing concentrations (5 to 50 μg/μL) of both drugs, steadily increase the size of zone of inhibition. This indicates that sildenafil and tadalafil effectively inhibit the growth of *E.coli*.

## Discussion

Kinnings et al. have constructed the ‘TB-drugome’ [4] which corresponds to the network of drug-protein interactions. Here, drugs represent all known approved drugs that have been structurally characterized and the proteins represent putative *M*.*tb* receptors. The ‘TB-drugome’ is based on the premise that unrelated proteins can bind similar ligands if they share the same ligand binding sites. Accordingly, the *M.tb* Rv1555 protein was predicted to be druggable with sildenafil, tadalafil and vardenafil drugs [4]. However, the mode of drug binding and the mechanism of inhibition of the *M.tb* protein’s function are not known. These drugs are known inhibitors of human phosphodiesterase PDE5 enzyme that belongs to the intracellular second messengers cyclic AMP and cyclic GMP degrading enzyme superfamily and that serve as drug targets for a variety of diseases, such as, asthma, depression, heart failure, inflammation and erectile dysfunction [16–22]. For the treatment of erectile dysfunction, PDE5 is a well known target for sildenafil (Viagra) and similar other drugs. The three-dimensional structures corresponding to the catalytic domain of human PDE5 (residues 537–860) complexed with sildenafil (PDB codes:1UDT, 2H42), tadalafil (1UDU) and vardenafil (1UHO) are available in the PDB and all three drugs are known to bind near the same site, although their binding poses are different [6].

The template for modeling the *M.tb* Rv1555 protein represents one of the membrane anchor subunits in the crystal structure of the *E. coli* QFR respiratory protein complex (i.e., the D-chain in PDB code:1KF6). In *E.coli*, the function of the QFR protein is to catalyze the terminal step of anaerobic respiration when fumarate acts as the terminal electron acceptor [23]. During anaerobic respiration, electrons are transferred via the iron-sulfur clusters to flavin adenine dinucleotide (FAD) that is covalently-bound at the active site leading to the reduction of fumarate to succinate [24, 25]. The *E. coli* QFR protein crystal structure complex is made up of four subunits representing the polypeptide chains; FrdA, FrdB, FrdC and FrdD [2]. The FrdA chain represents the flavoprotein and FrdB chain represents the iron-sulfur protein that contains the iron-sulfur clusters; [2Fe:2S], [4Fe:4S], and [3Fe:4S]. Both these subunits; FrdA and FrdB correspond to the soluble domain involved in the fumarate reduction [2]. There are two further sites and these are involved in the electron transfer reaction of the enzyme and are associated with the membrane anchor subunits; FrdC and FrdD that have been proposed to bind quinones [26–28]. Quinones are central to energy transduction processes in respiration as membrane-soluble electron carriers that can couple proton and electron transfer reactions and during fumarate reduction, electrons from reduced quinone are transferred to the iron-sulfur clusters [2]. There are two distinct quinone binding sites associated with the membrane anchor subunits referred as Q_P_ and Q_D_ where Q_P_ is the site that is proximal to the [3Fe:4S] iron-sulfur cluster of the soluble domain and Q_D_ is the distal site which is opposite of the membrane to the site of fumarate reduction [3].

The native structure of *E.coli* QFR containing only quinones (PDB code:1L0V) shows density at both Q_P_ and Q_D_ corresponding to the positions of the two quinones on opposite ends of the membrane [2]_._ However, according to [3], while it is not known whether both Q_P_ and Q_D_ quinone binding sites are mechanistically significant, the crystal structures of two *E.coli* QFR protein inhibitor complexes (PDB codes: 1KF6, 1KFY) with known quinol binding site inhibitors; 2-heptyl-4-hydroxyquinoline-N-oxide (HQNO) and 2-[1-(*p*-chlorophenyl)ethyl]4,6-dinitrophenol have been shown to block the binding of menaquinols at the Q_P_ site and that the binding of drugs near the Q_P_ equivalent site is important for function in *E.coli* QFR proteins. Further, for inhibitors bound at the Q_P_ site no density was observed at Q_D_ site corresponding to quinol binding [3]. In this context, it is interesting to note that the drugs sildenafil, tadalafil, vardenafil bind near the equivalent Q_P_ site in the *M.tb* protein dimer comprising Rv1555 and Rv1554 proteins.

### Comparison of the binding site interactions made by sildenafil, tadalafil, vardenafil drugs in human phosphodiesterase PDE5 enzyme and *M.tb* homologs of the *E.coli* QFR membrane subunit proteins

The crystal structure of human PDE5 and the model of *M.tb* Rv1555 protein cannot be structurally superimposed. Therefore, we examined the features that may contribute to the binding of above drugs to these proteins. A summary of the drug-protein interactions via hydrogen bonds observed in the crystal structures of human PDE5 (as in PDB codes:1UDT, 1UDU and 1UHO) and in the *M.tb* protein models are listed in Table 2. We notice that the same set of atoms; N22 and O27 in sildenafil and vardenafil are engaged in hydrogen bond interactions with human PDE5 and the *M.tb* proteins, although different residues are involved, i.e., Gln817 in human PDE5 and Arg85, Arg25, Glu26 in *M.tb* proteins. In the case of tadalafil binding, both N9 and O28 atoms involved in the hydrogen bonding with Gln817 in human PDE5 and with Arg89 in the *M.tb* protein are on the same side. Another feature observed was that both human PDE5 and the *M.tb* proteins were mainly comprised of helices and Gln817 which makes side-chain hydrogen bond interactions with all the three drugs is associated with a ‘kinked’ helix Lys809-Ser836 in human PDE5 (PDB code:1UDT). In the *M.tb* protein too, the side-chain of Arg85 predicted to be involved in hydrogen bond interactions with sildenafil and vardenafil is associated with a ‘kinked’ helix (Ala15-Leu47). These common features suggest the ability for sildenafil, tadalafil and vardenafil drugs to possess the capability to bind both human PDE5 and the *M.tb* Rv1555/Rv1554 proteins.

In conclusion, our computational analyses suggest that sildenafil, tadalafil and vardenafil drugs bind near the Q_D_ site of the Rv1555 *M.tb* monomer protein and near the Q_P_ site of the Rv1555 *M.tb* protein in the context of the dimer comprising the Rv1555 and Rv1554 proteins. These sites correspond to the equivalent quinol binding sites in the crystal structure of the homologous *E.coli* quinol fumarate reductase enzyme. Comparing the binding sites and poses of the drugs in the flexible and rigid protein complexes, sildenafil binding was observed to be almost identical. Vardenafil binding site was also the same except for the orientation. Tadalafil showed relative shift in binding to the flexible protein. We note that sildenafil and vardenafil drugs make four hydrogen bonds with the dimer compared to two hydrogen bonds made by tadalafil. The amino acid residues; Ser24, Arg25, Glu26, Arg85, Ser86 and Arg89 involved in the side-chain hydrogen bond interactions with the *M.tb* proteins depending upon the drug type are suggested as potential candidates for mutation studies in order to evaluate their role in the protein’s function.

**Fig. 11:**
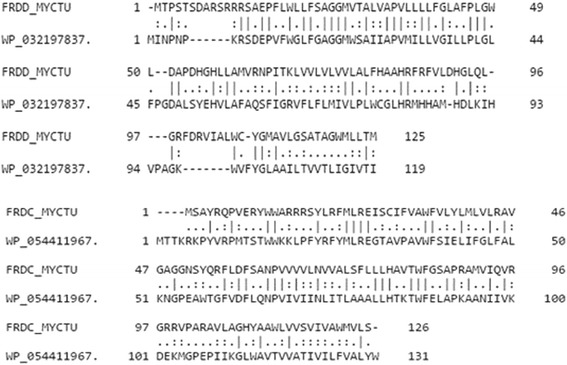
Sequence alignments corresponding to the *M.tb* Rv1555 (FRDD_MYCTU) and Rv1554 (FRDC_MYCTU) proteins along with their homologs from *E.coli*

The agar diffusion test (disc diffusion test) is a simple and widely used [29, 30] test to study the susceptibility of bacteria to various drugs. If a drug stops the growth of bacteria, the area around the paper disc will not have bacterial growth and therefore will have a halo appearance. This is referred to as a zone of inhibition. The size of the zone of inhibition is dependent on the drug effectiveness to prevent the growth of the bacterium. We could not test the inhibitory profile of these drugs to mycobacteria and therefore evaluated the same on *E.coli* bacteria that contains homologs of the *M.tb* Rv1555 and Rv1554 sequences as shown in the alignments in Fig. 11. The accession codes for *E.coli* proteins corresponding to *M.tb* proteins Rv1555 (FRDD_MYCTU) and Rv1554 (FRDC_MYCTU) obtained using the BLASTP program were WP_032197837.1 (maximum score = 58.5, query coverage = 69%, e-value = 2e-10) and WP_054411967.1 (maximum score = 81.3, query coverage = 98%, e-value = 4e-19), respectively. Our results confirm the inhibitory role of sildenafil and tadalafil on *E.coli*. It is possible that mycobacteria growth may also be affected when treated with these drugs.

Based on our analyses, we suggest that the predicted binding of sildenafil, tadalafil and vardenafil drugs near the Q_P_ site in the *M.tb* Rv1555 protein, is likely to interfere with quinol binding thereby possibly affecting the electron transfer known to be important for anaerobic respiration function in the homologous *E.coli* QFR protein. The above drugs may play a role equivalent of quinol inhibitors in the above *M.tb* proteins and validated for their effectiveness as repurposed drugs to treat tuberculosis.

In summary, our modeling studies suggest, that the drugs; sildenafil, tadalafil and vardenafil are capable of binding the *M.tb* membrane anchor subunit proteins; Rv1555, Rv1554 that are homologous to the *E.coli* quinol-binding fumarate reductase proteins. The predicted binding sites of these drugs to the above *M.tb* proteins is likely to interfere with quinol binding known to be essential for electron transfer function during anaerobic respiration in the homologous *E.coli* QFR protein. Although our experimental results do not directly demonstrate the inhibition of the above *M.tb* target proteins with the drugs, they successfully demonstrated, the in-vitro inhibition of *E.coli* bacterial growth with sildenafil and tadalafil drugs. However, studies with *M.tb* strains would be required to provide a more accurate and reliable proof for further validating these as repurposed drugs for tuberculosis.

## Conclusions

The predicted binding sites for sildenafil (Viagra), tadalafil (Cialis), vardenafil (Levitra) drugs to the *M.tb* Rv1555 and Rv1554 proteins suggests interference with quinol binding that may be important for the protein’s function as in the homolgous membrane anchor subunit proteins of the *E.coli* quinol-fumarate reductase respiratory protein complex. Further, sildenafil and tadalafil were observed to possess antibacterial activity in-vitro. The above drugs currently used to treat male erectile dysfunction by targeting the human phosphodiesterase 5 protein may further be validated by conducting activity analysis with *M.tb* strains in order to guide studies related to their role as plausible repurposed drugs for tuberculosis. The following residues in *M.tb* Rv1555 protein; Trp20, Phe23, His84, Arg85, Phe88 near the Q_P_ site and Val36, Leu37, Leu40, Phe41, Leu58, Val71, Leu74, Met121 near the Q_D_ site are involved in the binding to all the three drugs. Ser24, Arg25, Glu26, Arg85, Ser86 and Arg89 are among the residues involved in the side-chain hydrogen bond interactions in the *M.tb* proteins depending upon the drug type. These residues serve as potential candidates for conducting mutation studies, in order to evaluate their role in the protein’s function.

## Additional files

Additional file 1: Three-dimensional coordinates of the *M.tb* dimer protein complexed with sildenafil in the PDB format. (DOC 224 kb)

Additional file 2: Three-dimensional coordinates of the *M.tb* dimer protein complexed with tadalafil in the PDB format. (DOC 223 kb)

Additional file 3: Three-dimensional coordinates of the *M.tb* dimer protein complexed with vardenafil in the PDB format. (DOC 224 kb)

## Abbreviations

*E.coli*: *Escherichia coli*
FAD: Flavin adenine dinucleotide
HQNO: 2-heptyl-4-hydroxyquinoline-N-oxide
*M.tb*: *Mycobacterium tuberculosis*
MDR-TB: Multi-drug resistance tuberculosis
PDB: Protein data bank
PDE5: Phosphodiesterase 5
QFR: Quinol-fumarate reductase
TB: Tuberculosis
XDR-TB: Extremely drug-resistance tuberculosis
