# Supplementary material for: Three-dimensional models of Mycobacterium tuberculosis proteins Rv1555, Rv1554 and their docking analyses with sildenafil, tadalafil, vardenafil drugs, suggest interference with quinol binding likely to affect protein’s function
Source: BMC Struct Biol. 2018 Apr 18;18:5. doi: 10.1186/s12900-018-0085-4 (PMC5907181; doi:10.1186/s12900-018-0085-4)
Supplement: Supplementary file 1 — Three-dimensional coordinates of the M.tb dimer protein complexed with sildenafil in the PDB format. (DOC 224 kb) [file 12900_2018_85_MOESM1_ESM.doc]

REMARK SILDENAFIL MODELED IN THE RV1555 DIMER WITH RV1554

ATOM 6401 N MET C 1 -5.604 3.257 -30.273 1.00145.71 N

ATOM 6402 CA MET C 1 -5.189 4.127 -31.397 1.00145.71 C

ATOM 6403 CB MET C 1 -3.851 4.821 -31.073 1.00145.71 C

ATOM 6404 CG MET C 1 -2.657 3.871 -30.930 1.00145.71 C

ATOM 6405 SD MET C 1 -2.016 3.180 -32.486 1.00145.71 S

ATOM 6406 CE MET C 1 -1.301 4.741 -33.078 1.00145.71 C

ATOM 6407 C MET C 1 -5.067 3.431 -32.713 1.00145.71 C

ATOM 6408 O MET C 1 -4.845 2.221 -32.785 1.00145.71 O

ATOM 6409 N SER C 2 -5.257 4.196 -33.807 1.00 93.20 N

ATOM 6410 CA SER C 2 -5.094 3.654 -35.127 1.00 93.20 C

ATOM 6411 CB SER C 2 -6.425 3.440 -35.865 1.00 93.20 C

ATOM 6412 OG SER C 2 -6.183 2.910 -37.160 1.00 93.20 O

ATOM 6413 C SER C 2 -4.323 4.677 -35.902 1.00 93.20 C

ATOM 6414 O SER C 2 -4.897 5.634 -36.421 1.00 93.20 O

ATOM 6415 N ALA C 3 -2.998 4.474 -36.032 1.00 38.64 N

ATOM 6416 CA ALA C 3 -2.161 5.431 -36.701 1.00 38.64 C

ATOM 6417 CB ALA C 3 -0.658 5.151 -36.528 1.00 38.64 C

ATOM 6418 C ALA C 3 -2.459 5.411 -38.166 1.00 38.64 C

ATOM 6419 O ALA C 3 -2.574 4.350 -38.776 1.00 38.64 O

ATOM 6420 N TYR C 4 -2.543 6.609 -38.779 1.00 57.34 N

ATOM 6421 CA TYR C 4 -2.876 6.725 -40.170 1.00 57.34 C

ATOM 6422 CB TYR C 4 -3.966 7.793 -40.393 1.00 57.34 C

ATOM 6423 CG TYR C 4 -4.373 7.830 -41.825 1.00 57.34 C

ATOM 6424 CD1 TYR C 4 -5.242 6.884 -42.318 1.00 57.34 C

ATOM 6425 CD2 TYR C 4 -3.910 8.815 -42.667 1.00 57.34 C

ATOM 6426 CE1 TYR C 4 -5.634 6.909 -43.635 1.00 57.34 C

ATOM 6427 CE2 TYR C 4 -4.299 8.845 -43.986 1.00 57.34 C

ATOM 6428 CZ TYR C 4 -5.161 7.891 -44.471 1.00 57.34 C

ATOM 6429 OH TYR C 4 -5.562 7.921 -45.823 1.00 57.34 O

ATOM 6430 C TYR C 4 -1.634 7.168 -40.883 1.00 57.34 C

ATOM 6431 O TYR C 4 -1.143 8.273 -40.661 1.00 57.34 O

ATOM 6432 N ARG C 5 -1.092 6.311 -41.777 1.00100.21 N

ATOM 6433 CA ARG C 5 0.134 6.661 -42.437 1.00100.21 C

ATOM 6434 CB ARG C 5 1.142 5.497 -42.468 1.00100.21 C

ATOM 6435 CG ARG C 5 2.606 5.915 -42.628 1.00100.21 C

ATOM 6436 CD ARG C 5 2.959 6.512 -43.990 1.00100.21 C

ATOM 6437 NE ARG C 5 4.424 6.791 -43.985 1.00100.21 N

ATOM 6438 CZ ARG C 5 4.905 7.954 -43.456 1.00100.21 C

ATOM 6439 NH1 ARG C 5 4.048 8.861 -42.903 1.00100.21 N

ATOM 6440 NH2 ARG C 5 6.246 8.208 -43.478 1.00100.21 N

ATOM 6441 C ARG C 5 -0.188 7.039 -43.850 1.00100.21 C

ATOM 6442 O ARG C 5 -0.729 6.242 -44.614 1.00100.21 O

ATOM 6443 N GLN C 6 0.148 8.287 -44.232 1.00 87.65 N

ATOM 6444 CA GLN C 6 -0.119 8.770 -45.560 1.00 87.65 C

ATOM 6445 CB GLN C 6 -0.013 10.301 -45.673 1.00 87.65 C

ATOM 6446 CG GLN C 6 1.403 10.817 -45.406 1.00 87.65 C

ATOM 6447 CD GLN C 6 1.408 12.336 -45.516 1.00 87.65 C

ATOM 6448 OE1 GLN C 6 2.090 13.020 -44.755 1.00 87.65 O

ATOM 6449 NE2 GLN C 6 0.634 12.882 -46.492 1.00 87.65 N

ATOM 6450 C GLN C 6 0.906 8.177 -46.489 1.00 87.65 C

ATOM 6451 O GLN C 6 2.064 7.994 -46.119 1.00 87.65 O

ATOM 6452 N PRO C 7 0.489 7.840 -47.685 1.00 70.18 N

ATOM 6453 CA PRO C 7 1.391 7.273 -48.662 1.00 70.18 C

ATOM 6454 CD PRO C 7 -0.874 7.378 -47.881 1.00 70.18 C

ATOM 6455 CB PRO C 7 0.529 6.415 -49.593 1.00 70.18 C

ATOM 6456 CG PRO C 7 -0.911 6.889 -49.336 1.00 70.18 C

ATOM 6457 C PRO C 7 2.191 8.306 -49.402 1.00 70.18 C

ATOM 6458 O PRO C 7 1.762 9.456 -49.474 1.00 70.18 O

ATOM 6459 N VAL C 8 3.354 7.913 -49.971 1.00102.63 N

ATOM 6460 CA VAL C 8 4.163 8.841 -50.716 1.00102.63 C

ATOM 6461 CB VAL C 8 5.576 8.932 -50.209 1.00102.63 C

ATOM 6462 CG1 VAL C 8 5.536 9.505 -48.783 1.00102.63 C

ATOM 6463 CG2 VAL C 8 6.234 7.543 -50.283 1.00102.63 C

ATOM 6464 C VAL C 8 4.201 8.402 -52.151 1.00102.63 C

ATOM 6465 O VAL C 8 4.610 7.288 -52.474 1.00102.63 O

ATOM 6466 N GLU C 9 3.778 9.304 -53.059 1.00 83.29 N

ATOM 6467 CA GLU C 9 3.688 9.005 -54.463 1.00 83.29 C

ATOM 6468 CB GLU C 9 2.614 9.846 -55.174 1.00 83.29 C

ATOM 6469 CG GLU C 9 1.203 9.651 -54.612 1.00 83.29 C

ATOM 6470 CD GLU C 9 0.666 8.313 -55.095 1.00 83.29 C

ATOM 6471 OE1 GLU C 9 1.335 7.679 -55.954 1.00 83.29 O

ATOM 6472 OE2 GLU C 9 -0.423 7.907 -54.610 1.00 83.29 O

ATOM 6473 C GLU C 9 4.997 9.305 -55.139 1.00 83.29 C

ATOM 6474 O GLU C 9 5.854 10.008 -54.608 1.00 83.29 O

ATOM 6475 N ARG C 10 5.164 8.758 -56.360 1.00178.71 N

ATOM 6476 CA ARG C 10 6.313 8.938 -57.210 1.00178.71 C

ATOM 6477 CB ARG C 10 6.197 8.147 -58.523 1.00178.71 C

ATOM 6478 CG ARG C 10 7.286 8.495 -59.539 1.00178.71 C

ATOM 6479 CD ARG C 10 7.002 7.961 -60.944 1.00178.71 C

ATOM 6480 NE ARG C 10 5.938 8.822 -61.531 1.00178.71 N

ATOM 6481 CZ ARG C 10 6.276 9.985 -62.161 1.00178.71 C

ATOM 6482 NH1 ARG C 10 7.589 10.342 -62.274 1.00178.71 N

ATOM 6483 NH2 ARG C 10 5.303 10.790 -62.679 1.00178.71 N

ATOM 6484 C ARG C 10 6.370 10.383 -57.600 1.00178.71 C

ATOM 6485 O ARG C 10 7.433 10.928 -57.891 1.00178.71 O

ATOM 6486 N TYR C 11 5.172 10.984 -57.652 1.00147.85 N

ATOM 6487 CA TYR C 11 4.750 12.306 -58.031 1.00147.85 C

ATOM 6488 CB TYR C 11 3.216 12.368 -58.162 1.00147.85 C

ATOM 6489 CG TYR C 11 2.780 13.737 -58.557 1.00147.85 C

ATOM 6490 CD1 TYR C 11 2.887 14.163 -59.860 1.00147.85 C

ATOM 6491 CD2 TYR C 11 2.238 14.588 -57.620 1.00147.85 C

ATOM 6492 CE1 TYR C 11 2.474 15.425 -60.221 1.00147.85 C

ATOM 6493 CE2 TYR C 11 1.824 15.850 -57.975 1.00147.85 C

ATOM 6494 CZ TYR C 11 1.942 16.270 -59.278 1.00147.85 C

ATOM 6495 OH TYR C 11 1.517 17.565 -59.646 1.00147.85 O

ATOM 6496 C TYR C 11 5.184 13.369 -57.072 1.00147.85 C

ATOM 6497 O TYR C 11 5.165 14.544 -57.431 1.00147.85 O

ATOM 6498 N TRP C 12 5.567 13.009 -55.828 1.00 98.12 N

ATOM 6499 CA TRP C 12 5.737 13.941 -54.745 1.00 98.12 C

ATOM 6500 CB TRP C 12 6.466 13.315 -53.543 1.00 98.12 C

ATOM 6501 CG TRP C 12 7.858 12.837 -53.877 1.00 98.12 C

ATOM 6502 CD2 TRP C 12 9.037 13.655 -53.806 1.00 98.12 C

ATOM 6503 CD1 TRP C 12 8.267 11.608 -54.304 1.00 98.12 C

ATOM 6504 NE1 TRP C 12 9.626 11.610 -54.511 1.00 98.12 N

ATOM 6505 CE2 TRP C 12 10.113 12.863 -54.208 1.00 98.12 C

ATOM 6506 CE3 TRP C 12 9.210 14.959 -53.438 1.00 98.12 C

ATOM 6507 CZ2 TRP C 12 11.383 13.365 -54.249 1.00 98.12 C

ATOM 6508 CZ3 TRP C 12 10.492 15.461 -53.480 1.00 98.12 C

ATOM 6509 CH2 TRP C 12 11.557 14.679 -53.878 1.00 98.12 C

ATOM 6510 C TRP C 12 6.522 15.166 -55.136 1.00 98.12 C

ATOM 6511 O TRP C 12 6.150 16.272 -54.749 1.00 98.12 O

ATOM 6512 N TRP C 13 7.600 15.028 -55.920 1.00 89.80 N

ATOM 6513 CA TRP C 13 8.456 16.129 -56.279 1.00 89.80 C

ATOM 6514 CB TRP C 13 9.684 15.669 -57.079 1.00 89.80 C

ATOM 6515 CG TRP C 13 9.344 14.934 -58.351 1.00 89.80 C

ATOM 6516 CD2 TRP C 13 9.223 15.563 -59.635 1.00 89.80 C

ATOM 6517 CD1 TRP C 13 9.107 13.605 -58.546 1.00 89.80 C

ATOM 6518 NE1 TRP C 13 8.841 13.365 -59.873 1.00 89.80 N

ATOM 6519 CE2 TRP C 13 8.913 14.562 -60.555 1.00 89.80 C

ATOM 6520 CE3 TRP C 13 9.365 16.867 -60.014 1.00 89.80 C

ATOM 6521 CZ2 TRP C 13 8.735 14.854 -61.877 1.00 89.80 C

ATOM 6522 CZ3 TRP C 13 9.185 17.159 -61.347 1.00 89.80 C

ATOM 6523 CH2 TRP C 13 8.876 16.170 -62.259 1.00 89.80 C

ATOM 6524 C TRP C 13 7.733 17.175 -57.073 1.00 89.80 C

ATOM 6525 O TRP C 13 8.099 18.348 -57.026 1.00 89.80 O

ATOM 6526 N ALA C 14 6.707 16.783 -57.844 1.00 52.81 N

ATOM 6527 CA ALA C 14 6.007 17.687 -58.711 1.00 52.81 C

ATOM 6528 CB ALA C 14 4.894 16.989 -59.511 1.00 52.81 C

ATOM 6529 C ALA C 14 5.367 18.806 -57.939 1.00 52.81 C

ATOM 6530 O ALA C 14 5.268 19.924 -58.442 1.00 52.81 O

ATOM 6531 N ARG C 15 4.871 18.538 -56.717 1.00 91.49 N

ATOM 6532 CA ARG C 15 4.102 19.543 -56.036 1.00 91.49 C

ATOM 6533 CB ARG C 15 3.527 19.003 -54.720 1.00 91.49 C

ATOM 6534 CG ARG C 15 2.614 17.798 -54.955 1.00 91.49 C

ATOM 6535 CD ARG C 15 1.995 17.229 -53.680 1.00 91.49 C

ATOM 6536 NE ARG C 15 1.135 16.075 -54.072 1.00 91.49 N

ATOM 6537 CZ ARG C 15 0.259 15.536 -53.172 1.00 91.49 C

ATOM 6538 NH1 ARG C 15 0.167 16.056 -51.914 1.00 91.49 N

ATOM 6539 NH2 ARG C 15 -0.525 14.478 -53.531 1.00 91.49 N

ATOM 6540 C ARG C 15 4.844 20.820 -55.735 1.00 91.49 C

ATOM 6541 O ARG C 15 4.520 21.861 -56.303 1.00 91.49 O

ATOM 6542 N ARG C 16 5.899 20.791 -54.895 1.00153.94 N

ATOM 6543 CA ARG C 16 6.521 22.037 -54.522 1.00153.94 C

ATOM 6544 CB ARG C 16 7.105 22.058 -53.099 1.00153.94 C

ATOM 6545 CG ARG C 16 6.037 22.161 -52.010 1.00153.94 C

ATOM 6546 CD ARG C 16 6.612 22.332 -50.602 1.00153.94 C

ATOM 6547 NE ARG C 16 7.535 23.503 -50.632 1.00153.94 N

ATOM 6548 CZ ARG C 16 7.741 24.243 -49.504 1.00153.94 C

ATOM 6549 NH1 ARG C 16 7.058 23.948 -48.359 1.00153.94 N

ATOM 6550 NH2 ARG C 16 8.633 25.277 -49.519 1.00153.94 N

ATOM 6551 C ARG C 16 7.622 22.375 -55.468 1.00153.94 C

ATOM 6552 O ARG C 16 8.338 21.503 -55.951 1.00153.94 O

ATOM 6553 N ARG C 17 7.797 23.687 -55.718 1.00124.07 N

ATOM 6554 CA ARG C 17 8.775 24.185 -56.639 1.00124.07 C

ATOM 6555 CB ARG C 17 8.760 25.719 -56.743 1.00124.07 C

ATOM 6556 CG ARG C 17 7.455 26.304 -57.288 1.00124.07 C

ATOM 6557 CD ARG C 17 7.282 26.120 -58.796 1.00124.07 C

ATOM 6558 NE ARG C 17 6.446 24.906 -59.003 1.00124.07 N

ATOM 6559 CZ ARG C 17 5.086 25.020 -59.004 1.00124.07 C

ATOM 6560 NH1 ARG C 17 4.507 26.245 -58.835 1.00124.07 N

ATOM 6561 NH2 ARG C 17 4.307 23.912 -59.173 1.00124.07 N

ATOM 6562 C ARG C 17 10.137 23.805 -56.153 1.00124.07 C

ATOM 6563 O ARG C 17 11.001 23.430 -56.943 1.00124.07 O

ATOM 6564 N SER C 18 10.361 23.879 -54.829 1.00 70.24 N

ATOM 6565 CA SER C 18 11.664 23.604 -54.300 1.00 70.24 C

ATOM 6566 CB SER C 18 11.709 23.697 -52.766 1.00 70.24 C

ATOM 6567 OG SER C 18 11.391 25.016 -52.347 1.00 70.24 O

ATOM 6568 C SER C 18 12.050 22.213 -54.692 1.00 70.24 C

ATOM 6569 O SER C 18 13.211 21.952 -55.004 1.00 70.24 O

ATOM 6570 N TYR C 19 11.087 21.273 -54.665 1.00 54.62 N

ATOM 6571 CA TYR C 19 11.364 19.920 -55.055 1.00 54.62 C

ATOM 6572 CB TYR C 19 10.205 18.945 -54.785 1.00 54.62 C

ATOM 6573 CG TYR C 19 9.993 18.895 -53.312 1.00 54.62 C

ATOM 6574 CD1 TYR C 19 10.927 18.312 -52.486 1.00 54.62 C

ATOM 6575 CD2 TYR C 19 8.847 19.414 -52.758 1.00 54.62 C

ATOM 6576 CE1 TYR C 19 10.728 18.262 -51.126 1.00 54.62 C

ATOM 6577 CE2 TYR C 19 8.641 19.367 -51.400 1.00 54.62 C

ATOM 6578 CZ TYR C 19 9.583 18.793 -50.582 1.00 54.62 C

ATOM 6579 OH TYR C 19 9.372 18.745 -49.188 1.00 54.62 O

ATOM 6580 C TYR C 19 11.653 19.875 -56.525 1.00 54.62 C

ATOM 6581 O TYR C 19 12.568 19.176 -56.960 1.00 54.62 O

ATOM 6582 N LEU C 20 10.878 20.626 -57.336 1.00 86.27 N

ATOM 6583 CA LEU C 20 11.082 20.577 -58.757 1.00 86.27 C

ATOM 6584 CB LEU C 20 10.124 21.464 -59.576 1.00 86.27 C

ATOM 6585 CG LEU C 20 8.719 20.877 -59.815 1.00 86.27 C

ATOM 6586 CD1 LEU C 20 7.941 20.650 -58.515 1.00 86.27 C

ATOM 6587 CD2 LEU C 20 7.941 21.742 -60.818 1.00 86.27 C

ATOM 6588 C LEU C 20 12.465 21.041 -59.069 1.00 86.27 C

ATOM 6589 O LEU C 20 13.169 20.410 -59.855 1.00 86.27 O

ATOM 6590 N ARG C 21 12.914 22.130 -58.423 1.00 71.71 N

ATOM 6591 CA ARG C 21 14.206 22.675 -58.724 1.00 71.71 C

ATOM 6592 CB ARG C 21 14.570 23.875 -57.830 1.00 71.71 C

ATOM 6593 CG ARG C 21 13.731 25.130 -58.085 1.00 71.71 C

ATOM 6594 CD ARG C 21 14.075 26.285 -57.141 1.00 71.71 C

ATOM 6595 NE ARG C 21 13.306 27.485 -57.578 1.00 71.71 N

ATOM 6596 CZ ARG C 21 13.862 28.353 -58.473 1.00 71.71 C

ATOM 6597 NH1 ARG C 21 15.104 28.102 -58.979 1.00 71.71 N

ATOM 6598 NH2 ARG C 21 13.182 29.473 -58.858 1.00 71.71 N

ATOM 6599 C ARG C 21 15.219 21.607 -58.461 1.00 71.71 C

ATOM 6600 O ARG C 21 16.201 21.474 -59.188 1.00 71.71 O

ATOM 6601 N PHE C 22 14.997 20.824 -57.392 1.00 69.09 N

ATOM 6602 CA PHE C 22 15.867 19.754 -57.002 1.00 69.09 C

ATOM 6603 CB PHE C 22 15.324 19.065 -55.734 1.00 69.09 C

ATOM 6604 CG PHE C 22 16.026 17.777 -55.471 1.00 69.09 C

ATOM 6605 CD1 PHE C 22 17.278 17.738 -54.900 1.00 69.09 C

ATOM 6606 CD2 PHE C 22 15.396 16.593 -55.780 1.00 69.09 C

ATOM 6607 CE1 PHE C 22 17.893 16.530 -54.659 1.00 69.09 C

ATOM 6608 CE2 PHE C 22 16.005 15.385 -55.541 1.00 69.09 C

ATOM 6609 CZ PHE C 22 17.259 15.353 -54.980 1.00 69.09 C

ATOM 6610 C PHE C 22 15.928 18.750 -58.107 1.00 69.09 C

ATOM 6611 O PHE C 22 17.008 18.342 -58.534 1.00 69.09 O

ATOM 6612 N MET C 23 14.761 18.350 -58.632 1.00 45.22 N

ATOM 6613 CA MET C 23 14.761 17.366 -59.669 1.00 45.22 C

ATOM 6614 CB MET C 23 13.348 16.989 -60.135 1.00 45.22 C

ATOM 6615 CG MET C 23 12.578 16.199 -59.076 1.00 45.22 C

ATOM 6616 SD MET C 23 13.338 14.602 -58.653 1.00 45.22 S

ATOM 6617 CE MET C 23 12.804 13.752 -60.167 1.00 45.22 C

ATOM 6618 C MET C 23 15.520 17.923 -60.830 1.00 45.22 C

ATOM 6619 O MET C 23 16.352 17.237 -61.421 1.00 45.22 O

ATOM 6620 N LEU C 24 15.289 19.206 -61.161 1.00105.83 N

ATOM 6621 CA LEU C 24 15.975 19.783 -62.280 1.00105.83 C

ATOM 6622 CB LEU C 24 15.618 21.246 -62.595 1.00105.83 C

ATOM 6623 CG LEU C 24 14.299 21.434 -63.363 1.00105.83 C

ATOM 6624 CD1 LEU C 24 13.075 21.057 -62.519 1.00105.83 C

ATOM 6625 CD2 LEU C 24 14.208 22.845 -63.962 1.00105.83 C

ATOM 6626 C LEU C 24 17.447 19.768 -62.033 1.00105.83 C

ATOM 6627 O LEU C 24 18.232 19.544 -62.950 1.00105.83 O

ATOM 6628 N ARG C 25 17.891 20.028 -60.795 1.00 98.98 N

ATOM 6629 CA ARG C 25 19.310 20.064 -60.608 1.00 98.98 C

ATOM 6630 CB ARG C 25 19.724 20.457 -59.181 1.00 98.98 C

ATOM 6631 CG ARG C 25 21.235 20.627 -59.024 1.00 98.98 C

ATOM 6632 CD ARG C 25 21.851 19.641 -58.036 1.00 98.98 C

ATOM 6633 NE ARG C 25 21.271 19.951 -56.699 1.00 98.98 N

ATOM 6634 CZ ARG C 25 21.549 19.143 -55.638 1.00 98.98 C

ATOM 6635 NH1 ARG C 25 22.375 18.073 -55.809 1.00 98.98 N

ATOM 6636 NH2 ARG C 25 21.024 19.417 -54.409 1.00 98.98 N

ATOM 6637 C ARG C 25 19.890 18.717 -60.921 1.00 98.98 C

ATOM 6638 O ARG C 25 20.862 18.609 -61.663 1.00 98.98 O

ATOM 6639 N GLU C 26 19.278 17.641 -60.394 1.00132.72 N

ATOM 6640 CA GLU C 26 19.768 16.310 -60.623 1.00132.72 C

ATOM 6641 CB GLU C 26 19.012 15.228 -59.826 1.00132.72 C

ATOM 6642 CG GLU C 26 19.598 14.909 -58.444 1.00132.72 C

ATOM 6643 CD GLU C 26 20.022 16.180 -57.734 1.00132.72 C

ATOM 6644 OE1 GLU C 26 19.287 17.196 -57.811 1.00132.72 O

ATOM 6645 OE2 GLU C 26 21.109 16.143 -57.101 1.00132.72 O

ATOM 6646 C GLU C 26 19.672 15.967 -62.077 1.00132.72 C

ATOM 6647 O GLU C 26 20.519 15.246 -62.600 1.00132.72 O

ATOM 6648 N ILE C 27 18.653 16.492 -62.781 1.00138.87 N

ATOM 6649 CA ILE C 27 18.424 16.119 -64.151 1.00138.87 C

ATOM 6650 CB ILE C 27 17.120 16.580 -64.760 1.00138.87 C

ATOM 6651 CG1 ILE C 27 16.995 18.097 -64.895 1.00138.87 C

ATOM 6652 CG2 ILE C 27 15.995 15.968 -63.913 1.00138.87 C

ATOM 6653 CD1 ILE C 27 15.736 18.531 -65.645 1.00138.87 C

ATOM 6654 C ILE C 27 19.598 16.471 -65.012 1.00138.87 C

ATOM 6655 O ILE C 27 19.806 15.861 -66.059 1.00138.87 O

ATOM 6656 N SER C 28 20.396 17.473 -64.609 1.00101.32 N

ATOM 6657 CA SER C 28 21.544 17.911 -65.356 1.00101.32 C

ATOM 6658 CB SER C 28 22.330 18.944 -64.536 1.00101.32 C

ATOM 6659 OG SER C 28 23.629 19.113 -65.068 1.00101.32 O

ATOM 6660 C SER C 28 22.452 16.738 -65.644 1.00101.32 C

ATOM 6661 O SER C 28 23.285 16.783 -66.550 1.00101.32 O

ATOM 6662 N CYS C 29 22.296 15.637 -64.895 1.00 95.89 N

ATOM 6663 CA CYS C 29 23.121 14.469 -65.031 1.00 95.89 C

ATOM 6664 CB CYS C 29 22.748 13.365 -64.029 1.00 95.89 C

ATOM 6665 SG CYS C 29 21.052 12.775 -64.302 1.00 95.89 S

ATOM 6666 C CYS C 29 22.997 13.867 -66.412 1.00 95.89 C

ATOM 6667 O CYS C 29 23.954 13.289 -66.927 1.00 95.89 O

ATOM 6668 N ILE C 30 21.815 13.911 -67.048 1.00 65.90 N

ATOM 6669 CA ILE C 30 21.710 13.258 -68.325 1.00 65.90 C

ATOM 6670 CB ILE C 30 20.305 12.999 -68.756 1.00 65.90 C

ATOM 6671 CG1 ILE C 30 19.605 12.059 -67.759 1.00 65.90 C

ATOM 6672 CG2 ILE C 30 20.370 12.389 -70.160 1.00 65.90 C

ATOM 6673 CD1 ILE C 30 18.096 11.962 -67.979 1.00 65.90 C

ATOM 6674 C ILE C 30 22.466 13.932 -69.444 1.00 65.90 C

ATOM 6675 O ILE C 30 23.185 13.253 -70.176 1.00 65.90 O

ATOM 6676 N PHE C 31 22.341 15.276 -69.614 1.00129.18 N

ATOM 6677 CA PHE C 31 23.008 15.947 -70.700 1.00129.18 C

ATOM 6678 CB PHE C 31 22.561 17.411 -70.971 1.00129.18 C

ATOM 6679 CG PHE C 31 21.174 17.175 -71.482 1.00129.18 C

ATOM 6680 CD1 PHE C 31 20.157 16.995 -70.581 1.00129.18 C

ATOM 6681 CD2 PHE C 31 20.861 17.086 -72.820 1.00129.18 C

ATOM 6682 CE1 PHE C 31 18.868 16.737 -70.981 1.00129.18 C

ATOM 6683 CE2 PHE C 31 19.563 16.831 -73.227 1.00129.18 C

ATOM 6684 CZ PHE C 31 18.553 16.648 -72.317 1.00129.18 C

ATOM 6685 C PHE C 31 24.478 15.826 -70.480 1.00129.18 C

ATOM 6686 O PHE C 31 25.225 15.684 -71.447 1.00129.18 O

ATOM 6687 N VAL C 32 24.940 15.862 -69.215 1.00101.58 N

ATOM 6688 CA VAL C 32 26.349 15.753 -68.976 1.00101.58 C

ATOM 6689 CB VAL C 32 26.747 16.041 -67.552 1.00101.58 C

ATOM 6690 CG1 VAL C 32 26.567 17.555 -67.306 1.00101.58 C

ATOM 6691 CG2 VAL C 32 25.934 15.138 -66.615 1.00101.58 C

ATOM 6692 C VAL C 32 26.874 14.422 -69.425 1.00101.58 C

ATOM 6693 O VAL C 32 27.947 14.353 -70.027 1.00101.58 O

ATOM 6694 N ALA C 33 26.152 13.321 -69.149 1.00 41.48 N

ATOM 6695 CA ALA C 33 26.599 12.025 -69.594 1.00 41.48 C

ATOM 6696 CB ALA C 33 25.721 10.873 -69.075 1.00 41.48 C

ATOM 6697 C ALA C 33 26.545 11.972 -71.092 1.00 41.48 C

ATOM 6698 O ALA C 33 27.418 11.417 -71.755 1.00 41.48 O

ATOM 6699 N TRP C 34 25.501 12.571 -71.681 1.00219.34 N

ATOM 6700 CA TRP C 34 25.289 12.380 -73.085 1.00219.34 C

ATOM 6701 CB TRP C 34 23.991 12.992 -73.591 1.00219.34 C

ATOM 6702 CG TRP C 34 23.286 11.890 -74.312 1.00219.34 C

ATOM 6703 CD2 TRP C 34 23.145 11.606 -75.715 1.00219.34 C

ATOM 6704 CD1 TRP C 34 22.633 10.899 -73.655 1.00219.34 C

ATOM 6705 NE1 TRP C 34 22.078 10.031 -74.543 1.00219.34 N

ATOM 6706 CE2 TRP C 34 22.387 10.442 -75.814 1.00219.34 C

ATOM 6707 CE3 TRP C 34 23.599 12.254 -76.823 1.00219.34 C

ATOM 6708 CZ2 TRP C 34 22.056 9.903 -77.022 1.00219.34 C

ATOM 6709 CZ3 TRP C 34 23.281 11.688 -78.041 1.00219.34 C

ATOM 6710 CH2 TRP C 34 22.524 10.539 -78.143 1.00219.34 C

ATOM 6711 C TRP C 34 26.434 13.026 -73.778 1.00219.34 C

ATOM 6712 O TRP C 34 26.890 12.586 -74.833 1.00219.34 O

ATOM 6713 N PHE C 35 26.913 14.125 -73.187 1.00103.70 N

ATOM 6714 CA PHE C 35 28.056 14.837 -73.669 1.00103.70 C

ATOM 6715 CB PHE C 35 28.274 16.128 -72.854 1.00103.70 C

ATOM 6716 CG PHE C 35 29.691 16.577 -72.936 1.00103.70 C

ATOM 6717 CD1 PHE C 35 30.200 17.258 -74.016 1.00103.70 C

ATOM 6718 CD2 PHE C 35 30.514 16.278 -71.878 1.00103.70 C

ATOM 6719 CE1 PHE C 35 31.520 17.643 -74.019 1.00103.70 C

ATOM 6720 CE2 PHE C 35 31.830 16.660 -71.873 1.00103.70 C

ATOM 6721 CZ PHE C 35 32.335 17.344 -72.950 1.00103.70 C

ATOM 6722 C PHE C 35 29.268 13.955 -73.591 1.00103.70 C

ATOM 6723 O PHE C 35 30.105 13.983 -74.495 1.00103.70 O

ATOM 6724 N VAL C 36 29.423 13.154 -72.515 1.00120.72 N

ATOM 6725 CA VAL C 36 30.598 12.330 -72.449 1.00120.72 C

ATOM 6726 CB VAL C 36 30.823 11.566 -71.160 1.00120.72 C

ATOM 6727 CG1 VAL C 36 30.817 12.552 -69.980 1.00120.72 C

ATOM 6728 CG2 VAL C 36 29.841 10.399 -71.049 1.00120.72 C

ATOM 6729 C VAL C 36 30.534 11.351 -73.586 1.00120.72 C

ATOM 6730 O VAL C 36 31.571 10.983 -74.134 1.00120.72 O

ATOM 6731 N LEU C 37 29.312 10.900 -73.960 1.00 66.97 N

ATOM 6732 CA LEU C 37 29.110 9.987 -75.061 1.00 66.97 C

ATOM 6733 CB LEU C 37 27.648 9.827 -75.525 1.00 66.97 C

ATOM 6734 CG LEU C 37 26.661 9.112 -74.605 1.00 66.97 C

ATOM 6735 CD1 LEU C 37 25.240 9.130 -75.198 1.00 66.97 C

ATOM 6736 CD2 LEU C 37 27.134 7.677 -74.367 1.00 66.97 C

ATOM 6737 C LEU C 37 29.629 10.645 -76.290 1.00 66.97 C

ATOM 6738 O LEU C 37 30.366 10.039 -77.065 1.00 66.97 O

ATOM 6739 N TYR C 38 29.248 11.922 -76.506 1.00 74.81 N

ATOM 6740 CA TYR C 38 29.631 12.553 -77.739 1.00 74.81 C

ATOM 6741 CB TYR C 38 29.199 14.026 -77.936 1.00 74.81 C

ATOM 6742 CG TYR C 38 27.895 14.188 -78.655 1.00 74.81 C

ATOM 6743 CD1 TYR C 38 26.685 14.074 -78.009 1.00 74.81 C

ATOM 6744 CD2 TYR C 38 27.901 14.486 -80.003 1.00 74.81 C

ATOM 6745 CE1 TYR C 38 25.510 14.256 -78.701 1.00 74.81 C

ATOM 6746 CE2 TYR C 38 26.731 14.667 -80.702 1.00 74.81 C

ATOM 6747 CZ TYR C 38 25.529 14.552 -80.044 1.00 74.81 C

ATOM 6748 OH TYR C 38 24.317 14.741 -80.742 1.00 74.81 O

ATOM 6749 C TYR C 38 31.108 12.585 -77.849 1.00 74.81 C

ATOM 6750 O TYR C 38 31.654 12.321 -78.918 1.00 74.81 O

ATOM 6751 N LEU C 39 31.796 12.901 -76.746 1.00 54.76 N

ATOM 6752 CA LEU C 39 33.214 13.008 -76.830 1.00 54.76 C

ATOM 6753 CB LEU C 39 33.872 13.374 -75.497 1.00 54.76 C

ATOM 6754 CG LEU C 39 33.607 14.828 -75.089 1.00 54.76 C

ATOM 6755 CD1 LEU C 39 34.382 15.188 -73.814 1.00 54.76 C

ATOM 6756 CD2 LEU C 39 33.899 15.789 -76.253 1.00 54.76 C

ATOM 6757 C LEU C 39 33.757 11.689 -77.248 1.00 54.76 C

ATOM 6758 O LEU C 39 34.734 11.620 -77.989 1.00 54.76 O

ATOM 6759 N MET C 40 33.142 10.600 -76.770 1.00113.13 N

ATOM 6760 CA MET C 40 33.618 9.297 -77.109 1.00113.13 C

ATOM 6761 CB MET C 40 32.764 8.195 -76.468 1.00113.13 C

ATOM 6762 CG MET C 40 33.259 6.787 -76.775 1.00113.13 C

ATOM 6763 SD MET C 40 32.146 5.496 -76.156 1.00113.13 S

ATOM 6764 CE MET C 40 32.179 6.105 -74.446 1.00113.13 C

ATOM 6765 C MET C 40 33.515 9.116 -78.586 1.00113.13 C

ATOM 6766 O MET C 40 34.453 8.641 -79.223 1.00113.13 O

ATOM 6767 N LEU C 41 32.382 9.515 -79.188 1.00129.40 N

ATOM 6768 CA LEU C 41 32.248 9.254 -80.588 1.00129.40 C

ATOM 6769 CB LEU C 41 30.859 9.627 -81.137 1.00129.40 C

ATOM 6770 CG LEU C 41 30.594 9.102 -82.561 1.00129.40 C

ATOM 6771 CD1 LEU C 41 30.611 7.564 -82.588 1.00129.40 C

ATOM 6772 CD2 LEU C 41 29.296 9.686 -83.142 1.00129.40 C

ATOM 6773 C LEU C 41 33.309 10.027 -81.308 1.00129.40 C

ATOM 6774 O LEU C 41 34.035 9.474 -82.131 1.00129.40 O

ATOM 6775 N VAL C 42 33.485 11.316 -80.958 1.00100.59 N

ATOM 6776 CA VAL C 42 34.436 12.128 -81.657 1.00100.59 C

ATOM 6777 CB VAL C 42 34.474 13.564 -81.207 1.00100.59 C

ATOM 6778 CG1 VAL C 42 35.044 13.652 -79.787 1.00100.59 C

ATOM 6779 CG2 VAL C 42 35.298 14.367 -82.224 1.00100.59 C

ATOM 6780 C VAL C 42 35.802 11.534 -81.495 1.00100.59 C

ATOM 6781 O VAL C 42 36.597 11.545 -82.432 1.00100.59 O

ATOM 6782 N LEU C 43 36.120 10.994 -80.304 1.00 47.54 N

ATOM 6783 CA LEU C 43 37.432 10.456 -80.077 1.00 47.54 C

ATOM 6784 CB LEU C 43 37.639 9.983 -78.631 1.00 47.54 C

ATOM 6785 CG LEU C 43 39.053 9.448 -78.351 1.00 47.54 C

ATOM 6786 CD1 LEU C 43 40.121 10.518 -78.624 1.00 47.54 C

ATOM 6787 CD2 LEU C 43 39.149 8.913 -76.917 1.00 47.54 C

ATOM 6788 C LEU C 43 37.670 9.287 -80.994 1.00 47.54 C

ATOM 6789 O LEU C 43 38.765 9.124 -81.527 1.00 47.54 O

ATOM 6790 N ARG C 44 36.666 8.408 -81.175 1.00115.47 N

ATOM 6791 CA ARG C 44 36.815 7.271 -82.046 1.00115.47 C

ATOM 6792 CB ARG C 44 35.673 6.239 -81.937 1.00115.47 C

ATOM 6793 CG ARG C 44 35.695 5.382 -80.665 1.00115.47 C

ATOM 6794 CD ARG C 44 34.686 4.226 -80.686 1.00115.47 C

ATOM 6795 NE ARG C 44 35.180 3.214 -81.665 1.00115.47 N

ATOM 6796 CZ ARG C 44 34.804 1.903 -81.572 1.00115.47 C

ATOM 6797 NH1 ARG C 44 33.949 1.495 -80.589 1.00115.47 N

ATOM 6798 NH2 ARG C 44 35.296 0.990 -82.459 1.00115.47 N

ATOM 6799 C ARG C 44 36.866 7.727 -83.473 1.00115.47 C

ATOM 6800 O ARG C 44 37.535 7.121 -84.306 1.00115.47 O

ATOM 6801 N ALA C 45 36.105 8.781 -83.808 1.00 32.03 N

ATOM 6802 CA ALA C 45 36.065 9.292 -85.148 1.00 32.03 C

ATOM 6803 CB ALA C 45 35.045 10.429 -85.330 1.00 32.03 C

ATOM 6804 C ALA C 45 37.412 9.836 -85.495 1.00 32.03 C

ATOM 6805 O ALA C 45 37.873 9.701 -86.628 1.00 32.03 O

ATOM 6806 N VAL C 46 38.077 10.487 -84.522 1.00 97.39 N

ATOM 6807 CA VAL C 46 39.346 11.081 -84.817 1.00 97.39 C

ATOM 6808 CB VAL C 46 39.924 11.936 -83.727 1.00 97.39 C

ATOM 6809 CG1 VAL C 46 40.389 11.054 -82.564 1.00 97.39 C

ATOM 6810 CG2 VAL C 46 41.046 12.788 -84.345 1.00 97.39 C

ATOM 6811 C VAL C 46 40.334 10.014 -85.167 1.00 97.39 C

ATOM 6812 O VAL C 46 41.191 10.221 -86.023 1.00 97.39 O

ATOM 6813 N GLY C 47 40.264 8.854 -84.486 1.00 31.05 N

ATOM 6814 CA GLY C 47 41.158 7.755 -84.741 1.00 31.05 C

ATOM 6815 C GLY C 47 40.906 7.183 -86.108 1.00 31.05 C

ATOM 6816 O GLY C 47 41.827 6.764 -86.806 1.00 31.05 O

ATOM 6817 N ALA C 48 39.623 7.163 -86.504 1.00 68.78 N

ATOM 6818 CA ALA C 48 39.072 6.601 -87.707 1.00 68.78 C

ATOM 6819 CB ALA C 48 37.542 6.721 -87.769 1.00 68.78 C

ATOM 6820 C ALA C 48 39.630 7.281 -88.921 1.00 68.78 C

ATOM 6821 O ALA C 48 39.552 6.742 -90.020 1.00 68.78 O

ATOM 6822 N GLY C 49 40.114 8.527 -88.798 1.00 36.11 N

ATOM 6823 CA GLY C 49 40.656 9.173 -89.957 1.00 36.11 C

ATOM 6824 C GLY C 49 39.780 10.335 -90.284 1.00 36.11 C

ATOM 6825 O GLY C 49 38.655 10.444 -89.801 1.00 36.11 O

ATOM 6826 N GLY C 50 40.281 11.221 -91.163 1.00 27.22 N

ATOM 6827 CA GLY C 50 39.610 12.441 -91.497 1.00 27.22 C

ATOM 6828 C GLY C 50 38.262 12.112 -92.044 1.00 27.22 C

ATOM 6829 O GLY C 50 37.309 12.864 -91.846 1.00 27.22 O

ATOM 6830 N ASN C 51 38.147 10.985 -92.766 1.00 32.94 N

ATOM 6831 CA ASN C 51 36.889 10.658 -93.365 1.00 32.94 C

ATOM 6832 CB ASN C 51 36.916 9.290 -94.072 1.00 32.94 C

ATOM 6833 CG ASN C 51 37.933 9.341 -95.205 1.00 32.94 C

ATOM 6834 OD1 ASN C 51 38.217 10.403 -95.757 1.00 32.94 O

ATOM 6835 ND2 ASN C 51 38.501 8.158 -95.562 1.00 32.94 N

ATOM 6836 C ASN C 51 35.852 10.567 -92.285 1.00 32.94 C

ATOM 6837 O ASN C 51 34.783 11.166 -92.398 1.00 32.94 O

ATOM 6838 N SER C 52 36.148 9.820 -91.200 1.00 71.53 N

ATOM 6839 CA SER C 52 35.207 9.618 -90.126 1.00 71.53 C

ATOM 6840 CB SER C 52 35.658 8.532 -89.137 1.00 71.53 C

ATOM 6841 OG SER C 52 34.697 8.380 -88.103 1.00 71.53 O

ATOM 6842 C SER C 52 35.014 10.879 -89.336 1.00 71.53 C

ATOM 6843 O SER C 52 33.908 11.182 -88.888 1.00 71.53 O

ATOM 6844 N TYR C 53 36.096 11.652 -89.153 1.00 96.15 N

ATOM 6845 CA TYR C 53 36.079 12.848 -88.359 1.00 96.15 C

ATOM 6846 CB TYR C 53 37.472 13.503 -88.352 1.00 96.15 C

ATOM 6847 CG TYR C 53 37.470 14.747 -87.537 1.00 96.15 C

ATOM 6848 CD1 TYR C 53 37.167 15.959 -88.113 1.00 96.15 C

ATOM 6849 CD2 TYR C 53 37.784 14.701 -86.198 1.00 96.15 C

ATOM 6850 CE1 TYR C 53 37.175 17.111 -87.362 1.00 96.15 C

ATOM 6851 CE2 TYR C 53 37.792 15.849 -85.443 1.00 96.15 C

ATOM 6852 CZ TYR C 53 37.486 17.055 -86.025 1.00 96.15 C

ATOM 6853 OH TYR C 53 37.495 18.234 -85.252 1.00 96.15 O

ATOM 6854 C TYR C 53 35.102 13.808 -88.960 1.00 96.15 C

ATOM 6855 O TYR C 53 34.324 14.452 -88.255 1.00 96.15 O

ATOM 6856 N GLN C 54 35.114 13.908 -90.298 1.00 76.32 N

ATOM 6857 CA GLN C 54 34.264 14.819 -91.004 1.00 76.32 C

ATOM 6858 CB GLN C 54 34.513 14.773 -92.524 1.00 76.32 C

ATOM 6859 CG GLN C 54 35.936 15.201 -92.904 1.00 76.32 C

ATOM 6860 CD GLN C 54 36.098 15.128 -94.417 1.00 76.32 C

ATOM 6861 OE1 GLN C 54 35.557 14.241 -95.075 1.00 76.32 O

ATOM 6862 NE2 GLN C 54 36.866 16.094 -94.988 1.00 76.32 N

ATOM 6863 C GLN C 54 32.842 14.434 -90.746 1.00 76.32 C

ATOM 6864 O GLN C 54 31.972 15.293 -90.615 1.00 76.32 O

ATOM 6865 N ARG C 55 32.574 13.118 -90.673 1.00179.83 N

ATOM 6866 CA ARG C 55 31.242 12.622 -90.475 1.00179.83 C

ATOM 6867 CB ARG C 55 31.165 11.089 -90.543 1.00179.83 C

ATOM 6868 CG ARG C 55 29.734 10.558 -90.617 1.00179.83 C

ATOM 6869 CD ARG C 55 29.660 9.033 -90.690 1.00179.83 C

ATOM 6870 NE ARG C 55 30.406 8.617 -91.910 1.00179.83 N

ATOM 6871 CZ ARG C 55 31.536 7.862 -91.785 1.00179.83 C

ATOM 6872 NH1 ARG C 55 31.956 7.469 -90.548 1.00179.83 N

ATOM 6873 NH2 ARG C 55 32.247 7.504 -92.894 1.00179.83 N

ATOM 6874 C ARG C 55 30.743 13.050 -89.131 1.00179.83 C

ATOM 6875 O ARG C 55 29.568 13.368 -88.969 1.00179.83 O

ATOM 6876 N PHE C 56 31.628 13.040 -88.121 1.00 66.29 N

ATOM 6877 CA PHE C 56 31.275 13.410 -86.781 1.00 66.29 C

ATOM 6878 CB PHE C 56 32.451 13.113 -85.836 1.00 66.29 C

ATOM 6879 CG PHE C 56 32.203 13.706 -84.501 1.00 66.29 C

ATOM 6880 CD1 PHE C 56 31.391 13.093 -83.574 1.00 66.29 C

ATOM 6881 CD2 PHE C 56 32.798 14.902 -84.191 1.00 66.29 C

ATOM 6882 CE1 PHE C 56 31.183 13.668 -82.342 1.00 66.29 C

ATOM 6883 CE2 PHE C 56 32.594 15.476 -82.965 1.00 66.29 C

ATOM 6884 CZ PHE C 56 31.788 14.863 -82.036 1.00 66.29 C

ATOM 6885 C PHE C 56 30.890 14.859 -86.743 1.00 66.29 C

ATOM 6886 O PHE C 56 29.877 15.236 -86.153 1.00 66.29 O

ATOM 6887 N LEU C 57 31.678 15.717 -87.410 1.00151.75 N

ATOM 6888 CA LEU C 57 31.377 17.117 -87.412 1.00151.75 C

ATOM 6889 CB LEU C 57 32.436 17.970 -88.129 1.00151.75 C

ATOM 6890 CG LEU C 57 33.791 17.991 -87.393 1.00151.75 C

ATOM 6891 CD1 LEU C 57 34.805 18.906 -88.098 1.00151.75 C

ATOM 6892 CD2 LEU C 57 33.609 18.338 -85.906 1.00151.75 C

ATOM 6893 C LEU C 57 30.066 17.317 -88.093 1.00151.75 C

ATOM 6894 O LEU C 57 29.306 18.214 -87.733 1.00151.75 O

ATOM 6895 N ASP C 58 29.774 16.491 -89.113 1.00 37.00 N

ATOM 6896 CA ASP C 58 28.537 16.629 -89.821 1.00 37.00 C

ATOM 6897 CB ASP C 58 28.394 15.627 -90.980 1.00 37.00 C

ATOM 6898 CG ASP C 58 27.281 16.119 -91.896 1.00 37.00 C

ATOM 6899 OD1 ASP C 58 27.198 17.359 -92.111 1.00 37.00 O

ATOM 6900 OD2 ASP C 58 26.493 15.265 -92.383 1.00 37.00 O

ATOM 6901 C ASP C 58 27.417 16.373 -88.858 1.00 37.00 C

ATOM 6902 O ASP C 58 26.384 17.037 -88.912 1.00 37.00 O

ATOM 6903 N PHE C 59 27.603 15.398 -87.947 1.00114.53 N

ATOM 6904 CA PHE C 59 26.599 15.020 -86.989 1.00114.53 C

ATOM 6905 CB PHE C 59 27.095 13.859 -86.107 1.00114.53 C

ATOM 6906 CG PHE C 59 25.999 13.338 -85.237 1.00114.53 C

ATOM 6907 CD1 PHE C 59 25.722 13.916 -84.017 1.00114.53 C

ATOM 6908 CD2 PHE C 59 25.256 12.252 -85.636 1.00114.53 C

ATOM 6909 CE1 PHE C 59 24.716 13.429 -83.210 1.00114.53 C

ATOM 6910 CE2 PHE C 59 24.251 11.759 -84.836 1.00114.53 C

ATOM 6911 CZ PHE C 59 23.978 12.347 -83.622 1.00114.53 C

ATOM 6912 C PHE C 59 26.319 16.196 -86.107 1.00114.53 C

ATOM 6913 O PHE C 59 25.165 16.560 -85.889 1.00114.53 O

ATOM 6914 N SER C 60 27.380 16.849 -85.603 1.00 92.99 N

ATOM 6915 CA SER C 60 27.200 17.977 -84.727 1.00 92.99 C

ATOM 6916 CB SER C 60 28.534 18.575 -84.239 1.00 92.99 C

ATOM 6917 OG SER C 60 29.249 17.628 -83.459 1.00 92.99 O

ATOM 6918 C SER C 60 26.495 19.073 -85.479 1.00 92.99 C

ATOM 6919 O SER C 60 25.886 19.953 -84.873 1.00 92.99 O

ATOM 6920 N ALA C 61 26.611 19.052 -86.823 1.00 89.78 N

ATOM 6921 CA ALA C 61 26.092 20.011 -87.765 1.00 89.78 C

ATOM 6922 CB ALA C 61 26.614 19.788 -89.194 1.00 89.78 C

ATOM 6923 C ALA C 61 24.609 20.011 -87.843 1.00 89.78 C

ATOM 6924 O ALA C 61 24.031 21.069 -88.093 1.00 89.78 O

ATOM 6925 N ASN C 62 23.981 18.820 -87.659 1.00298.45 N

ATOM 6926 CA ASN C 62 22.551 18.614 -87.690 1.00298.45 C

ATOM 6927 CB ASN C 62 22.136 17.309 -86.972 1.00298.45 C

ATOM 6928 CG ASN C 62 20.623 17.107 -87.018 1.00298.45 C

ATOM 6929 OD1 ASN C 62 19.861 18.041 -87.257 1.00298.45 O

ATOM 6930 ND2 ASN C 62 20.177 15.847 -86.769 1.00298.45 N

ATOM 6931 C ASN C 62 22.015 19.738 -86.896 1.00298.45 C

ATOM 6932 O ASN C 62 22.679 20.164 -85.959 1.00298.45 O

ATOM 6933 N PRO C 63 20.960 20.369 -87.243 1.00203.05 N

ATOM 6934 CA PRO C 63 20.609 21.424 -86.347 1.00203.05 C

ATOM 6935 CD PRO C 63 20.630 20.696 -88.619 1.00203.05 C

ATOM 6936 CB PRO C 63 19.760 22.405 -87.149 1.00203.05 C

ATOM 6937 CG PRO C 63 19.462 21.681 -88.478 1.00203.05 C

ATOM 6938 C PRO C 63 19.957 20.881 -85.101 1.00203.05 C

ATOM 6939 O PRO C 63 19.892 21.605 -84.115 1.00203.05 O

ATOM 6940 N VAL C 64 19.438 19.647 -85.126 1.00 41.40 N

ATOM 6941 CA VAL C 64 18.778 19.153 -83.947 1.00 41.40 C

ATOM 6942 CB VAL C 64 18.055 17.857 -84.169 1.00 41.40 C

ATOM 6943 CG1 VAL C 64 17.438 17.412 -82.833 1.00 41.40 C

ATOM 6944 CG2 VAL C 64 17.018 18.064 -85.287 1.00 41.40 C

ATOM 6945 C VAL C 64 19.790 18.940 -82.865 1.00 41.40 C

ATOM 6946 O VAL C 64 19.560 19.243 -81.698 1.00 41.40 O

ATOM 6947 N VAL C 65 20.955 18.403 -83.243 1.00108.04 N

ATOM 6948 CA VAL C 65 22.018 18.105 -82.333 1.00108.04 C

ATOM 6949 CB VAL C 65 23.180 17.442 -83.010 1.00108.04 C

ATOM 6950 CG1 VAL C 65 22.716 16.088 -83.574 1.00108.04 C

ATOM 6951 CG2 VAL C 65 23.736 18.406 -84.072 1.00108.04 C

ATOM 6952 C VAL C 65 22.521 19.376 -81.729 1.00108.04 C

ATOM 6953 O VAL C 65 22.996 19.385 -80.595 1.00108.04 O

ATOM 6954 N VAL C 66 22.495 20.481 -82.493 1.00126.27 N

ATOM 6955 CA VAL C 66 23.003 21.709 -81.972 1.00126.27 C

ATOM 6956 CB VAL C 66 23.198 22.811 -82.984 1.00126.27 C

ATOM 6957 CG1 VAL C 66 24.101 22.283 -84.110 1.00126.27 C

ATOM 6958 CG2 VAL C 66 21.853 23.378 -83.442 1.00126.27 C

ATOM 6959 C VAL C 66 22.110 22.189 -80.871 1.00126.27 C

ATOM 6960 O VAL C 66 22.591 22.728 -79.879 1.00126.27 O

ATOM 6961 N VAL C 67 20.779 22.027 -81.004 1.00107.66 N

ATOM 6962 CA VAL C 67 19.911 22.510 -79.969 1.00107.66 C

ATOM 6963 CB VAL C 67 18.454 22.471 -80.336 1.00107.66 C

ATOM 6964 CG1 VAL C 67 18.275 23.265 -81.643 1.00107.66 C

ATOM 6965 CG2 VAL C 67 17.955 21.022 -80.389 1.00107.66 C

ATOM 6966 C VAL C 67 20.127 21.713 -78.712 1.00107.66 C

ATOM 6967 O VAL C 67 20.251 22.281 -77.627 1.00107.66 O

ATOM 6968 N LEU C 68 20.213 20.372 -78.819 1.00 61.21 N

ATOM 6969 CA LEU C 68 20.396 19.569 -77.646 1.00 61.21 C

ATOM 6970 CB LEU C 68 20.390 18.052 -77.907 1.00 61.21 C

ATOM 6971 CG LEU C 68 18.964 17.528 -78.132 1.00 61.21 C

ATOM 6972 CD1 LEU C 68 18.950 16.018 -78.394 1.00 61.21 C

ATOM 6973 CD2 LEU C 68 18.059 17.906 -76.946 1.00 61.21 C

ATOM 6974 C LEU C 68 21.689 19.938 -77.013 1.00 61.21 C

ATOM 6975 O LEU C 68 21.780 20.031 -75.791 1.00 61.21 O

ATOM 6976 N ASN C 69 22.732 20.166 -77.827 1.00 97.83 N

ATOM 6977 CA ASN C 69 23.982 20.513 -77.238 1.00 97.83 C

ATOM 6978 CB ASN C 69 25.166 20.353 -78.202 1.00 97.83 C

ATOM 6979 CG ASN C 69 25.357 18.837 -78.390 1.00 97.83 C

ATOM 6980 OD1 ASN C 69 25.747 18.389 -79.467 1.00 97.83 O

ATOM 6981 ND2 ASN C 69 25.073 18.026 -77.328 1.00 97.83 N

ATOM 6982 C ASN C 69 23.888 21.872 -76.604 1.00 97.83 C

ATOM 6983 O ASN C 69 24.570 22.139 -75.617 1.00 97.83 O

ATOM 6984 N VAL C 70 23.054 22.782 -77.152 1.00 34.06 N

ATOM 6985 CA VAL C 70 22.869 24.072 -76.535 1.00 34.06 C

ATOM 6986 CB VAL C 70 21.966 24.987 -77.306 1.00 34.06 C

ATOM 6987 CG1 VAL C 70 21.797 26.280 -76.491 1.00 34.06 C

ATOM 6988 CG2 VAL C 70 22.560 25.207 -78.705 1.00 34.06 C

ATOM 6989 C VAL C 70 22.222 23.857 -75.204 1.00 34.06 C

ATOM 6990 O VAL C 70 22.558 24.509 -74.216 1.00 34.06 O

ATOM 6991 N VAL C 71 21.255 22.924 -75.154 1.00 27.61 N

ATOM 6992 CA VAL C 71 20.568 22.629 -73.933 1.00 27.61 C

ATOM 6993 CB VAL C 71 19.524 21.570 -74.090 1.00 27.61 C

ATOM 6994 CG1 VAL C 71 18.877 21.331 -72.717 1.00 27.61 C

ATOM 6995 CG2 VAL C 71 18.537 22.015 -75.181 1.00 27.61 C

ATOM 6996 C VAL C 71 21.589 22.098 -72.985 1.00 27.61 C

ATOM 6997 O VAL C 71 21.555 22.381 -71.789 1.00 27.61 O

ATOM 6998 N ALA C 72 22.534 21.303 -73.514 1.00 43.58 N

ATOM 6999 CA ALA C 72 23.543 20.719 -72.689 1.00 43.58 C

ATOM 7000 CB ALA C 72 24.508 19.805 -73.465 1.00 43.58 C

ATOM 7001 C ALA C 72 24.346 21.817 -72.050 1.00 43.58 C

ATOM 7002 O ALA C 72 24.686 21.717 -70.871 1.00 43.58 O

ATOM 7003 N LEU C 73 24.678 22.899 -72.794 1.00134.12 N

ATOM 7004 CA LEU C 73 25.473 23.923 -72.166 1.00134.12 C

ATOM 7005 CB LEU C 73 25.823 25.191 -72.976 1.00134.12 C

ATOM 7006 CG LEU C 73 26.702 25.033 -74.215 1.00134.12 C

ATOM 7007 CD1 LEU C 73 25.852 24.544 -75.382 1.00134.12 C

ATOM 7008 CD2 LEU C 73 27.475 26.323 -74.538 1.00134.12 C

ATOM 7009 C LEU C 73 24.695 24.504 -71.041 1.00134.12 C

ATOM 7010 O LEU C 73 25.236 24.739 -69.963 1.00134.12 O

ATOM 7011 N SER C 74 23.396 24.767 -71.268 1.00 70.22 N

ATOM 7012 CA SER C 74 22.619 25.414 -70.250 1.00 70.22 C

ATOM 7013 CB SER C 74 21.155 25.641 -70.643 1.00 70.22 C

ATOM 7014 OG SER C 74 20.470 26.236 -69.553 1.00 70.22 O

ATOM 7015 C SER C 74 22.597 24.554 -69.037 1.00 70.22 C

ATOM 7016 O SER C 74 22.831 25.013 -67.920 1.00 70.22 O

ATOM 7017 N PHE C 75 22.337 23.257 -69.238 1.00 72.92 N

ATOM 7018 CA PHE C 75 22.259 22.367 -68.130 1.00 72.92 C

ATOM 7019 CB PHE C 75 21.910 20.940 -68.575 1.00 72.92 C

ATOM 7020 CG PHE C 75 20.428 20.902 -68.713 1.00 72.92 C

ATOM 7021 CD1 PHE C 75 19.754 21.964 -69.270 1.00 72.92 C

ATOM 7022 CD2 PHE C 75 19.709 19.840 -68.213 1.00 72.92 C

ATOM 7023 CE1 PHE C 75 18.385 21.946 -69.378 1.00 72.92 C

ATOM 7024 CE2 PHE C 75 18.340 19.812 -68.327 1.00 72.92 C

ATOM 7025 CZ PHE C 75 17.677 20.862 -68.913 1.00 72.92 C

ATOM 7026 C PHE C 75 23.562 22.367 -67.412 1.00 72.92 C

ATOM 7027 O PHE C 75 23.588 22.441 -66.185 1.00 72.92 O

ATOM 7028 N LEU C 76 24.695 22.300 -68.128 1.00194.86 N

ATOM 7029 CA LEU C 76 25.871 22.181 -67.331 1.00194.86 C

ATOM 7030 CB LEU C 76 26.974 21.284 -67.939 1.00194.86 C

ATOM 7031 CG LEU C 76 28.084 21.871 -68.788 1.00194.86 C

ATOM 7032 CD1 LEU C 76 29.040 22.647 -67.885 1.00194.86 C

ATOM 7033 CD2 LEU C 76 28.785 20.768 -69.601 1.00194.86 C

ATOM 7034 C LEU C 76 26.207 23.454 -66.596 1.00194.86 C

ATOM 7035 O LEU C 76 26.840 23.433 -65.542 1.00194.86 O

ATOM 7036 N LEU C 77 25.776 24.609 -67.130 1.00116.65 N

ATOM 7037 CA LEU C 77 25.954 25.881 -66.488 1.00116.65 C

ATOM 7038 CB LEU C 77 25.490 26.968 -67.468 1.00116.65 C

ATOM 7039 CG LEU C 77 25.918 28.396 -67.136 1.00116.65 C

ATOM 7040 CD1 LEU C 77 25.362 29.372 -68.185 1.00116.65 C

ATOM 7041 CD2 LEU C 77 25.562 28.768 -65.693 1.00116.65 C

ATOM 7042 C LEU C 77 25.126 25.884 -65.214 1.00116.65 C

ATOM 7043 O LEU C 77 25.587 26.326 -64.161 1.00116.65 O

ATOM 7044 N LEU C 78 23.886 25.348 -65.270 1.00 55.86 N

ATOM 7045 CA LEU C 78 23.023 25.275 -64.117 1.00 55.86 C

ATOM 7046 CB LEU C 78 21.643 24.651 -64.417 1.00 55.86 C

ATOM 7047 CG LEU C 78 20.734 24.524 -63.174 1.00 55.86 C

ATOM 7048 CD1 LEU C 78 20.442 25.895 -62.543 1.00 55.86 C

ATOM 7049 CD2 LEU C 78 19.453 23.727 -63.490 1.00 55.86 C

ATOM 7050 C LEU C 78 23.710 24.407 -63.111 1.00 55.86 C

ATOM 7051 O LEU C 78 23.607 24.626 -61.906 1.00 55.86 O

ATOM 7052 N HIS C 79 24.420 23.369 -63.582 1.00 50.02 N

ATOM 7053 CA HIS C 79 25.140 22.515 -62.683 1.00 50.02 C

ATOM 7054 ND1 HIS C 79 26.264 19.740 -61.477 1.00 50.02 N

ATOM 7055 CG HIS C 79 26.722 20.570 -62.476 1.00 50.02 C

ATOM 7056 CB HIS C 79 25.844 21.353 -63.401 1.00 50.02 C

ATOM 7057 NE2 HIS C 79 28.486 19.683 -61.386 1.00 50.02 N

ATOM 7058 CD2 HIS C 79 28.080 20.523 -62.406 1.00 50.02 C

ATOM 7059 CE1 HIS C 79 27.361 19.236 -60.857 1.00 50.02 C

ATOM 7060 C HIS C 79 26.202 23.306 -61.982 1.00 50.02 C

ATOM 7061 O HIS C 79 26.398 23.160 -60.777 1.00 50.02 O

ATOM 7062 N ALA C 80 26.931 24.161 -62.722 1.00 35.19 N

ATOM 7063 CA ALA C 80 27.999 24.916 -62.128 1.00 35.19 C

ATOM 7064 CB ALA C 80 28.763 25.774 -63.152 1.00 35.19 C

ATOM 7065 C ALA C 80 27.437 25.846 -61.101 1.00 35.19 C

ATOM 7066 O ALA C 80 27.982 25.975 -60.004 1.00 35.19 O

ATOM 7067 N VAL C 81 26.315 26.515 -61.424 1.00 96.01 N

ATOM 7068 CA VAL C 81 25.791 27.471 -60.497 1.00 96.01 C

ATOM 7069 CB VAL C 81 24.609 28.247 -61.020 1.00 96.01 C

ATOM 7070 CG1 VAL C 81 23.445 27.292 -61.318 1.00 96.01 C

ATOM 7071 CG2 VAL C 81 24.254 29.330 -59.987 1.00 96.01 C

ATOM 7072 C VAL C 81 25.393 26.785 -59.229 1.00 96.01 C

ATOM 7073 O VAL C 81 25.758 27.220 -58.138 1.00 96.01 O

ATOM 7074 N THR C 82 24.649 25.674 -59.343 1.00108.98 N

ATOM 7075 CA THR C 82 24.132 24.973 -58.205 1.00108.98 C

ATOM 7076 CB THR C 82 23.185 23.897 -58.627 1.00108.98 C

ATOM 7077 OG1 THR C 82 22.178 24.450 -59.456 1.00108.98 O

ATOM 7078 CG2 THR C 82 22.534 23.302 -57.373 1.00108.98 C

ATOM 7079 C THR C 82 25.240 24.321 -57.442 1.00108.98 C

ATOM 7080 O THR C 82 25.235 24.314 -56.212 1.00108.98 O

ATOM 7081 N TRP C 83 26.218 23.747 -58.167 1.00 67.35 N

ATOM 7082 CA TRP C 83 27.276 22.999 -57.552 1.00 67.35 C

ATOM 7083 CB TRP C 83 28.204 22.348 -58.589 1.00 67.35 C

ATOM 7084 CG TRP C 83 29.275 21.471 -57.995 1.00 67.35 C

ATOM 7085 CD2 TRP C 83 29.042 20.135 -57.523 1.00 67.35 C

ATOM 7086 CD1 TRP C 83 30.596 21.733 -57.779 1.00 67.35 C

ATOM 7087 NE1 TRP C 83 31.206 20.637 -57.217 1.00 67.35 N

ATOM 7088 CE2 TRP C 83 30.259 19.648 -57.048 1.00 67.35 C

ATOM 7089 CE3 TRP C 83 27.904 19.380 -57.483 1.00 67.35 C

ATOM 7090 CZ2 TRP C 83 30.359 18.391 -56.528 1.00 67.35 C

ATOM 7091 CZ3 TRP C 83 28.008 18.111 -56.960 1.00 67.35 C

ATOM 7092 CH2 TRP C 83 29.213 17.628 -56.492 1.00 67.35 C

ATOM 7093 C TRP C 83 28.088 23.898 -56.678 1.00 67.35 C

ATOM 7094 O TRP C 83 28.423 23.540 -55.549 1.00 67.35 O

ATOM 7095 N PHE C 84 28.418 25.105 -57.165 1.00120.73 N

ATOM 7096 CA PHE C 84 29.238 25.975 -56.375 1.00120.73 C

ATOM 7097 CB PHE C 84 29.750 27.233 -57.092 1.00120.73 C

ATOM 7098 CG PHE C 84 30.832 26.761 -58.002 1.00120.73 C

ATOM 7099 CD1 PHE C 84 31.881 26.042 -57.479 1.00120.73 C

ATOM 7100 CD2 PHE C 84 30.781 26.967 -59.360 1.00120.73 C

ATOM 7101 CE1 PHE C 84 32.896 25.582 -58.285 1.00120.73 C

ATOM 7102 CE2 PHE C 84 31.795 26.511 -60.172 1.00120.73 C

ATOM 7103 CZ PHE C 84 32.857 25.823 -59.636 1.00120.73 C

ATOM 7104 C PHE C 84 28.539 26.380 -55.124 1.00120.73 C

ATOM 7105 O PHE C 84 29.186 26.559 -54.097 1.00120.73 O

ATOM 7106 N GLY C 85 27.214 26.587 -55.162 1.00 27.54 N

ATOM 7107 CA GLY C 85 26.558 26.985 -53.950 1.00 27.54 C

ATOM 7108 C GLY C 85 26.661 25.894 -52.918 1.00 27.54 C

ATOM 7109 O GLY C 85 26.906 26.164 -51.744 1.00 27.54 O

ATOM 7110 N SER C 86 26.458 24.629 -53.337 1.00 49.57 N

ATOM 7111 CA SER C 86 26.394 23.491 -52.455 1.00 49.57 C

ATOM 7112 CB SER C 86 25.841 22.239 -53.156 1.00 49.57 C

ATOM 7113 OG SER C 86 26.749 21.799 -54.156 1.00 49.57 O

ATOM 7114 C SER C 86 27.714 23.095 -51.857 1.00 49.57 C

ATOM 7115 O SER C 86 27.772 22.686 -50.700 1.00 49.57 O

ATOM 7116 N ALA C 87 28.816 23.221 -52.614 1.00 55.25 N

ATOM 7117 CA ALA C 87 30.101 22.717 -52.195 1.00 55.25 C

ATOM 7118 CB ALA C 87 31.199 22.968 -53.244 1.00 55.25 C

ATOM 7119 C ALA C 87 30.588 23.307 -50.895 1.00 55.25 C

ATOM 7120 O ALA C 87 31.151 22.576 -50.081 1.00 55.25 O

ATOM 7121 N PRO C 88 30.407 24.570 -50.641 1.00 87.64 N

ATOM 7122 CA PRO C 88 30.948 25.182 -49.447 1.00 87.64 C

ATOM 7123 CD PRO C 88 30.283 25.535 -51.717 1.00 87.64 C

ATOM 7124 CB PRO C 88 30.699 26.678 -49.615 1.00 87.64 C

ATOM 7125 CG PRO C 88 30.728 26.880 -51.140 1.00 87.64 C

ATOM 7126 C PRO C 88 30.419 24.633 -48.159 1.00 87.64 C

ATOM 7127 O PRO C 88 31.047 24.852 -47.123 1.00 87.64 O

ATOM 7128 N ARG C 89 29.266 23.947 -48.198 1.00 76.49 N

ATOM 7129 CA ARG C 89 28.623 23.409 -47.032 1.00 76.49 C

ATOM 7130 CB ARG C 89 27.249 22.799 -47.344 1.00 76.49 C

ATOM 7131 CG ARG C 89 26.222 23.828 -47.811 1.00 76.49 C

ATOM 7132 CD ARG C 89 24.829 23.235 -48.022 1.00 76.49 C

ATOM 7133 NE ARG C 89 23.938 24.343 -48.466 1.00 76.49 N

ATOM 7134 CZ ARG C 89 22.617 24.103 -48.709 1.00 76.49 C

ATOM 7135 NH1 ARG C 89 22.110 22.844 -48.562 1.00 76.49 N

ATOM 7136 NH2 ARG C 89 21.802 25.126 -49.098 1.00 76.49 N

ATOM 7137 C ARG C 89 29.454 22.315 -46.433 1.00 76.49 C

ATOM 7138 O ARG C 89 29.387 22.055 -45.231 1.00 76.49 O

ATOM 7139 N ALA C 90 30.216 21.606 -47.281 1.00 48.06 N

ATOM 7140 CA ALA C 90 31.025 20.497 -46.869 1.00 48.06 C

ATOM 7141 CB ALA C 90 31.738 19.815 -48.049 1.00 48.06 C

ATOM 7142 C ALA C 90 32.083 20.938 -45.902 1.00 48.06 C

ATOM 7143 O ALA C 90 32.471 20.180 -45.014 1.00 48.06 O

ATOM 7144 N MET C 91 32.614 22.164 -46.059 1.00105.05 N

ATOM 7145 CA MET C 91 33.704 22.571 -45.216 1.00105.05 C

ATOM 7146 CB MET C 91 34.796 23.338 -45.982 1.00105.05 C

ATOM 7147 CG MET C 91 36.048 23.622 -45.153 1.00105.05 C

ATOM 7148 SD MET C 91 37.509 24.056 -46.143 1.00105.05 S

ATOM 7149 CE MET C 91 37.933 22.332 -46.533 1.00105.05 C

ATOM 7150 C MET C 91 33.241 23.416 -44.069 1.00105.05 C

ATOM 7151 O MET C 91 32.335 24.237 -44.198 1.00105.05 O

ATOM 7152 N VAL C 92 33.843 23.184 -42.880 1.00 62.55 N

ATOM 7153 CA VAL C 92 33.550 24.000 -41.736 1.00 62.55 C

ATOM 7154 CB VAL C 92 33.233 23.211 -40.503 1.00 62.55 C

ATOM 7155 CG1 VAL C 92 32.997 24.192 -39.344 1.00 62.55 C

ATOM 7156 CG2 VAL C 92 32.048 22.285 -40.792 1.00 62.55 C

ATOM 7157 C VAL C 92 34.831 24.712 -41.431 1.00 62.55 C

ATOM 7158 O VAL C 92 35.757 24.116 -40.884 1.00 62.55 O

ATOM 7159 N ILE C 93 34.915 26.018 -41.744 1.00 97.94 N

ATOM 7160 CA ILE C 93 36.156 26.714 -41.550 1.00 97.94 C

ATOM 7161 CB ILE C 93 36.710 27.301 -42.818 1.00 97.94 C

ATOM 7162 CG1 ILE C 93 37.044 26.195 -43.837 1.00 97.94 C

ATOM 7163 CG2 ILE C 93 37.914 28.182 -42.445 1.00 97.94 C

ATOM 7164 CD1 ILE C 93 38.148 25.246 -43.375 1.00 97.94 C

ATOM 7165 C ILE C 93 35.937 27.850 -40.606 1.00 97.94 C

ATOM 7166 O ILE C 93 35.001 28.633 -40.762 1.00 97.94 O

ATOM 7167 N GLN C 94 36.821 27.974 -39.594 1.00122.90 N

ATOM 7168 CA GLN C 94 36.654 29.035 -38.650 1.00122.90 C

ATOM 7169 CB GLN C 94 36.831 28.583 -37.189 1.00122.90 C

ATOM 7170 CG GLN C 94 38.253 28.116 -36.859 1.00122.90 C

ATOM 7171 CD GLN C 94 38.509 26.791 -37.565 1.00122.90 C

ATOM 7172 OE1 GLN C 94 37.579 26.081 -37.944 1.00122.90 O

ATOM 7173 NE2 GLN C 94 39.812 26.443 -37.743 1.00122.90 N

ATOM 7174 C GLN C 94 37.698 30.068 -38.906 1.00122.90 C

ATOM 7175 O GLN C 94 38.885 29.761 -39.008 1.00122.90 O

ATOM 7176 N VAL C 95 37.266 31.334 -39.053 1.00 56.94 N

ATOM 7177 CA VAL C 95 38.216 32.390 -39.190 1.00 56.94 C

ATOM 7178 CB VAL C 95 38.206 33.053 -40.542 1.00 56.94 C

ATOM 7179 CG1 VAL C 95 38.665 32.013 -41.578 1.00 56.94 C

ATOM 7180 CG2 VAL C 95 36.808 33.623 -40.836 1.00 56.94 C

ATOM 7181 C VAL C 95 37.894 33.394 -38.127 1.00 56.94 C

ATOM 7182 O VAL C 95 36.839 34.027 -38.133 1.00 56.94 O

ATOM 7183 N ARG C 96 38.828 33.571 -37.176 1.00114.81 N

ATOM 7184 CA ARG C 96 38.658 34.516 -36.111 1.00114.81 C

ATOM 7185 CB ARG C 96 38.625 35.973 -36.604 1.00114.81 C

ATOM 7186 CG ARG C 96 39.951 36.435 -37.212 1.00114.81 C

ATOM 7187 CD ARG C 96 40.964 36.920 -36.174 1.00114.81 C

ATOM 7188 NE ARG C 96 41.208 35.799 -35.223 1.00114.81 N

ATOM 7189 CZ ARG C 96 40.467 35.701 -34.080 1.00114.81 C

ATOM 7190 NH1 ARG C 96 39.518 36.642 -33.801 1.00114.81 N

ATOM 7191 NH2 ARG C 96 40.680 34.667 -33.215 1.00114.81 N

ATOM 7192 C ARG C 96 37.406 34.256 -35.324 1.00114.81 C

ATOM 7193 O ARG C 96 36.604 35.163 -35.109 1.00114.81 O

ATOM 7194 N GLY C 97 37.188 33.003 -34.883 1.00 28.03 N

ATOM 7195 CA GLY C 97 36.085 32.731 -34.003 1.00 28.03 C

ATOM 7196 C GLY C 97 34.792 32.755 -34.754 1.00 28.03 C

ATOM 7197 O GLY C 97 33.724 32.791 -34.143 1.00 28.03 O

ATOM 7198 N ARG C 98 34.831 32.740 -36.098 1.00125.61 N

ATOM 7199 CA ARG C 98 33.576 32.770 -36.791 1.00125.61 C

ATOM 7200 CB ARG C 98 33.287 34.116 -37.475 1.00125.61 C

ATOM 7201 CG ARG C 98 31.956 34.133 -38.229 1.00125.61 C

ATOM 7202 CD ARG C 98 30.731 33.994 -37.323 1.00125.61 C

ATOM 7203 NE ARG C 98 29.525 34.027 -38.198 1.00125.61 N

ATOM 7204 CZ ARG C 98 28.302 33.705 -37.686 1.00125.61 C

ATOM 7205 NH1 ARG C 98 28.180 33.374 -36.367 1.00125.61 N

ATOM 7206 NH2 ARG C 98 27.199 33.720 -38.491 1.00125.61 N

ATOM 7207 C ARG C 98 33.584 31.734 -37.867 1.00125.61 C

ATOM 7208 O ARG C 98 34.599 31.516 -38.527 1.00125.61 O

ATOM 7209 N ARG C 99 32.431 31.060 -38.063 1.00 92.02 N

ATOM 7210 CA ARG C 99 32.314 30.099 -39.115 1.00 92.02 C

ATOM 7211 CB ARG C 99 31.117 29.144 -38.948 1.00 92.02 C

ATOM 7212 CG ARG C 99 30.985 28.110 -40.069 1.00 92.02 C

ATOM 7213 CD ARG C 99 29.784 27.178 -39.900 1.00 92.02 C

ATOM 7214 NE ARG C 99 28.580 28.042 -39.747 1.00 92.02 N

ATOM 7215 CZ ARG C 99 28.190 28.443 -38.501 1.00 92.02 C

ATOM 7216 NH1 ARG C 99 28.875 28.012 -37.402 1.00 92.02 N

ATOM 7217 NH2 ARG C 99 27.120 29.277 -38.354 1.00 92.02 N

ATOM 7218 C ARG C 99 32.093 30.924 -40.337 1.00 92.02 C

ATOM 7219 O ARG C 99 31.184 31.751 -40.364 1.00 92.02 O

ATOM 7220 N VAL C 100 32.938 30.713 -41.368 1.00124.11 N

ATOM 7221 CA VAL C 100 32.945 31.474 -42.585 1.00124.11 C

ATOM 7222 CB VAL C 100 34.183 31.263 -43.404 1.00124.11 C

ATOM 7223 CG1 VAL C 100 35.400 31.720 -42.588 1.00124.11 C

ATOM 7224 CG2 VAL C 100 34.238 29.787 -43.833 1.00124.11 C

ATOM 7225 C VAL C 100 31.792 31.078 -43.447 1.00124.11 C

ATOM 7226 O VAL C 100 31.168 30.031 -43.298 1.00124.11 O

ATOM 7227 N PRO C 101 31.469 31.987 -44.312 1.00163.97 N

ATOM 7228 CA PRO C 101 30.397 31.775 -45.237 1.00163.97 C

ATOM 7229 CD PRO C 101 31.607 33.390 -43.958 1.00163.97 C

ATOM 7230 CB PRO C 101 29.791 33.151 -45.522 1.00163.97 C

ATOM 7231 CG PRO C 101 30.871 34.147 -45.072 1.00163.97 C

ATOM 7232 C PRO C 101 30.905 31.138 -46.401 1.00163.97 C

ATOM 7233 O PRO C 101 32.124 31.098 -46.516 1.00163.97 O

ATOM 7234 N ALA C 102 29.948 30.626 -47.185 1.00 63.44 N

ATOM 7235 CA ALA C 102 30.091 30.010 -48.453 1.00 63.44 C

ATOM 7236 CB ALA C 102 28.782 29.384 -48.963 1.00 63.44 C

ATOM 7237 C ALA C 102 30.503 31.052 -49.442 1.00 63.44 C

ATOM 7238 O ALA C 102 31.249 30.770 -50.373 1.00 63.44 O

ATOM 7239 N ARG C 103 30.047 32.302 -49.249 1.00 81.22 N

ATOM 7240 CA ARG C 103 30.208 33.338 -50.227 1.00 81.22 C

ATOM 7241 CB ARG C 103 29.668 34.679 -49.705 1.00 81.22 C

ATOM 7242 CG ARG C 103 28.185 34.551 -49.351 1.00 81.22 C

ATOM 7243 CD ARG C 103 27.543 35.781 -48.709 1.00 81.22 C

ATOM 7244 NE ARG C 103 26.120 35.420 -48.452 1.00 81.22 N

ATOM 7245 CZ ARG C 103 25.379 36.115 -47.541 1.00 81.22 C

ATOM 7246 NH1 ARG C 103 25.922 37.183 -46.888 1.00 81.22 N

ATOM 7247 NH2 ARG C 103 24.093 35.737 -47.281 1.00 81.22 N

ATOM 7248 C ARG C 103 31.648 33.483 -50.612 1.00 81.22 C

ATOM 7249 O ARG C 103 31.956 33.634 -51.793 1.00 81.22 O

ATOM 7250 N ALA C 104 32.581 33.425 -49.651 1.00 39.65 N

ATOM 7251 CA ALA C 104 33.958 33.586 -50.008 1.00 39.65 C

ATOM 7252 CB ALA C 104 34.899 33.509 -48.794 1.00 39.65 C

ATOM 7253 C ALA C 104 34.369 32.500 -50.967 1.00 39.65 C

ATOM 7254 O ALA C 104 35.017 32.780 -51.973 1.00 39.65 O

ATOM 7255 N VAL C 105 34.007 31.232 -50.683 1.00119.36 N

ATOM 7256 CA VAL C 105 34.373 30.116 -51.508 1.00119.36 C

ATOM 7257 CB VAL C 105 34.298 28.784 -50.811 1.00119.36 C

ATOM 7258 CG1 VAL C 105 35.455 28.720 -49.799 1.00119.36 C

ATOM 7259 CG2 VAL C 105 32.946 28.648 -50.112 1.00119.36 C

ATOM 7260 C VAL C 105 33.637 30.114 -52.818 1.00119.36 C

ATOM 7261 O VAL C 105 34.197 29.724 -53.841 1.00119.36 O

ATOM 7262 N LEU C 106 32.366 30.555 -52.835 1.00123.73 N

ATOM 7263 CA LEU C 106 31.584 30.574 -54.036 1.00123.73 C

ATOM 7264 CB LEU C 106 30.152 31.092 -53.785 1.00123.73 C

ATOM 7265 CG LEU C 106 29.229 31.155 -55.023 1.00123.73 C

ATOM 7266 CD1 LEU C 106 29.610 32.286 -55.993 1.00123.73 C

ATOM 7267 CD2 LEU C 106 29.138 29.784 -55.711 1.00123.73 C

ATOM 7268 C LEU C 106 32.262 31.496 -55.002 1.00123.73 C

ATOM 7269 O LEU C 106 32.336 31.212 -56.197 1.00123.73 O

ATOM 7270 N ALA C 107 32.765 32.640 -54.503 1.00 29.88 N

ATOM 7271 CA ALA C 107 33.405 33.615 -55.330 1.00 29.88 C

ATOM 7272 CB ALA C 107 33.823 34.876 -54.551 1.00 29.88 C

ATOM 7273 C ALA C 107 34.649 33.040 -55.937 1.00 29.88 C

ATOM 7274 O ALA C 107 34.906 33.221 -57.126 1.00 29.88 O

ATOM 7275 N GLY C 108 35.449 32.311 -55.134 1.00 20.69 N

ATOM 7276 CA GLY C 108 36.701 31.787 -55.604 1.00 20.69 C

ATOM 7277 C GLY C 108 36.484 30.798 -56.709 1.00 20.69 C

ATOM 7278 O GLY C 108 37.211 30.793 -57.701 1.00 20.69 O

ATOM 7279 N HIS C 109 35.469 29.929 -56.557 1.00 62.19 N

ATOM 7280 CA HIS C 109 35.200 28.882 -57.500 1.00 62.19 C

ATOM 7281 ND1 HIS C 109 33.216 26.831 -54.946 1.00 62.19 N

ATOM 7282 CG HIS C 109 34.224 27.367 -55.719 1.00 62.19 C

ATOM 7283 CB HIS C 109 34.008 28.024 -57.053 1.00 62.19 C

ATOM 7284 NE2 HIS C 109 35.108 26.537 -53.815 1.00 62.19 N

ATOM 7285 CD2 HIS C 109 35.372 27.177 -55.013 1.00 62.19 C

ATOM 7286 CE1 HIS C 109 33.800 26.349 -53.819 1.00 62.19 C

ATOM 7287 C HIS C 109 34.860 29.498 -58.823 1.00 62.19 C

ATOM 7288 O HIS C 109 35.327 29.054 -59.871 1.00 62.19 O

ATOM 7289 N TYR C 110 34.029 30.553 -58.792 1.00 95.00 N

ATOM 7290 CA TYR C 110 33.584 31.268 -59.954 1.00 95.00 C

ATOM 7291 CB TYR C 110 32.609 32.388 -59.542 1.00 95.00 C

ATOM 7292 CG TYR C 110 32.174 33.190 -60.720 1.00 95.00 C

ATOM 7293 CD1 TYR C 110 32.886 34.303 -61.099 1.00 95.00 C

ATOM 7294 CD2 TYR C 110 31.052 32.841 -61.438 1.00 95.00 C

ATOM 7295 CE1 TYR C 110 32.491 35.057 -62.178 1.00 95.00 C

ATOM 7296 CE2 TYR C 110 30.652 33.591 -62.520 1.00 95.00 C

ATOM 7297 CZ TYR C 110 31.372 34.701 -62.891 1.00 95.00 C

ATOM 7298 OH TYR C 110 30.964 35.474 -63.999 1.00 95.00 O

ATOM 7299 C TYR C 110 34.779 31.903 -60.594 1.00 95.00 C

ATOM 7300 O TYR C 110 34.914 31.922 -61.816 1.00 95.00 O

ATOM 7301 N ALA C 111 35.687 32.455 -59.773 1.00 20.16 N

ATOM 7302 CA ALA C 111 36.833 33.119 -60.317 1.00 20.16 C

ATOM 7303 CB ALA C 111 37.745 33.715 -59.231 1.00 20.16 C

ATOM 7304 C ALA C 111 37.640 32.121 -61.082 1.00 20.16 C

ATOM 7305 O ALA C 111 38.096 32.393 -62.192 1.00 20.16 O

ATOM 7306 N ALA C 112 37.818 30.920 -60.505 1.00 27.26 N

ATOM 7307 CA ALA C 112 38.622 29.906 -61.118 1.00 27.26 C

ATOM 7308 CB ALA C 112 38.760 28.648 -60.244 1.00 27.26 C

ATOM 7309 C ALA C 112 38.018 29.483 -62.421 1.00 27.26 C

ATOM 7310 O ALA C 112 38.734 29.295 -63.403 1.00 27.26 O

ATOM 7311 N TRP C 113 36.681 29.321 -62.481 1.00168.14 N

ATOM 7312 CA TRP C 113 36.133 28.821 -63.709 1.00168.14 C

ATOM 7313 CB TRP C 113 34.642 28.425 -63.684 1.00168.14 C

ATOM 7314 CG TRP C 113 33.633 29.540 -63.778 1.00168.14 C

ATOM 7315 CD2 TRP C 113 33.140 30.039 -65.031 1.00168.14 C

ATOM 7316 CD1 TRP C 113 32.990 30.241 -62.802 1.00168.14 C

ATOM 7317 NE1 TRP C 113 32.130 31.151 -63.370 1.00168.14 N

ATOM 7318 CE2 TRP C 113 32.211 31.035 -64.741 1.00168.14 C

ATOM 7319 CE3 TRP C 113 33.435 29.689 -66.318 1.00168.14 C

ATOM 7320 CZ2 TRP C 113 31.559 31.702 -65.740 1.00168.14 C

ATOM 7321 CZ3 TRP C 113 32.780 30.365 -67.323 1.00168.14 C

ATOM 7322 CH2 TRP C 113 31.859 31.352 -67.038 1.00168.14 C

ATOM 7323 C TRP C 113 36.332 29.845 -64.784 1.00168.14 C

ATOM 7324 O TRP C 113 36.576 29.503 -65.940 1.00168.14 O

ATOM 7325 N LEU C 114 36.224 31.140 -64.435 1.00 87.02 N

ATOM 7326 CA LEU C 114 36.367 32.163 -65.430 1.00 87.02 C

ATOM 7327 CB LEU C 114 36.165 33.582 -64.854 1.00 87.02 C

ATOM 7328 CG LEU C 114 36.290 34.744 -65.869 1.00 87.02 C

ATOM 7329 CD1 LEU C 114 37.742 34.997 -66.309 1.00 87.02 C

ATOM 7330 CD2 LEU C 114 35.338 34.544 -67.059 1.00 87.02 C

ATOM 7331 C LEU C 114 37.750 32.075 -66.000 1.00 87.02 C

ATOM 7332 O LEU C 114 37.931 32.138 -67.214 1.00 87.02 O

ATOM 7333 N VAL C 115 38.765 31.915 -65.131 1.00 25.60 N

ATOM 7334 CA VAL C 115 40.128 31.861 -65.579 1.00 25.60 C

ATOM 7335 CB VAL C 115 41.102 31.723 -64.442 1.00 25.60 C

ATOM 7336 CG1 VAL C 115 42.515 31.558 -65.025 1.00 25.60 C

ATOM 7337 CG2 VAL C 115 40.949 32.937 -63.510 1.00 25.60 C

ATOM 7338 C VAL C 115 40.304 30.664 -66.460 1.00 25.60 C

ATOM 7339 O VAL C 115 40.907 30.752 -67.529 1.00 25.60 O

ATOM 7340 N VAL C 116 39.770 29.502 -66.042 1.00 92.55 N

ATOM 7341 CA VAL C 116 39.972 28.311 -66.817 1.00 92.55 C

ATOM 7342 CB VAL C 116 39.460 27.059 -66.161 1.00 92.55 C

ATOM 7343 CG1 VAL C 116 40.206 26.873 -64.829 1.00 92.55 C

ATOM 7344 CG2 VAL C 116 37.933 27.129 -66.030 1.00 92.55 C

ATOM 7345 C VAL C 116 39.299 28.464 -68.140 1.00 92.55 C

ATOM 7346 O VAL C 116 39.840 28.073 -69.174 1.00 92.55 O

ATOM 7347 N SER C 117 38.094 29.053 -68.146 1.00 73.52 N

ATOM 7348 CA SER C 117 37.368 29.182 -69.372 1.00 73.52 C

ATOM 7349 CB SER C 117 36.001 29.849 -69.161 1.00 73.52 C

ATOM 7350 OG SER C 117 35.318 29.952 -70.399 1.00 73.52 O

ATOM 7351 C SER C 117 38.157 30.031 -70.326 1.00 73.52 C

ATOM 7352 O SER C 117 38.201 29.743 -71.520 1.00 73.52 O

ATOM 7353 N VAL C 118 38.788 31.116 -69.832 1.00 85.80 N

ATOM 7354 CA VAL C 118 39.519 31.986 -70.715 1.00 85.80 C

ATOM 7355 CB VAL C 118 39.935 33.291 -70.093 1.00 85.80 C

ATOM 7356 CG1 VAL C 118 41.014 33.039 -69.030 1.00 85.80 C

ATOM 7357 CG2 VAL C 118 40.398 34.225 -71.224 1.00 85.80 C

ATOM 7358 C VAL C 118 40.749 31.315 -71.244 1.00 85.80 C

ATOM 7359 O VAL C 118 41.056 31.422 -72.430 1.00 85.80 O

ATOM 7360 N ILE C 119 41.492 30.600 -70.379 1.00 73.84 N

ATOM 7361 CA ILE C 119 42.726 30.006 -70.807 1.00 73.84 C

ATOM 7362 CB ILE C 119 43.500 29.376 -69.680 1.00 73.84 C

ATOM 7363 CG1 ILE C 119 44.925 29.029 -70.140 1.00 73.84 C

ATOM 7364 CG2 ILE C 119 42.703 28.180 -69.136 1.00 73.84 C

ATOM 7365 CD1 ILE C 119 45.864 28.653 -68.994 1.00 73.84 C

ATOM 7366 C ILE C 119 42.460 28.969 -71.856 1.00 73.84 C

ATOM 7367 O ILE C 119 43.160 28.906 -72.864 1.00 73.84 O

ATOM 7368 N VAL C 120 41.427 28.130 -71.653 1.00 86.73 N

ATOM 7369 CA VAL C 120 41.143 27.070 -72.578 1.00 86.73 C

ATOM 7370 CB VAL C 120 40.003 26.205 -72.125 1.00 86.73 C

ATOM 7371 CG1 VAL C 120 40.416 25.502 -70.820 1.00 86.73 C

ATOM 7372 CG2 VAL C 120 38.750 27.082 -71.975 1.00 86.73 C

ATOM 7373 C VAL C 120 40.798 27.647 -73.917 1.00 86.73 C

ATOM 7374 O VAL C 120 41.266 27.164 -74.946 1.00 86.73 O

ATOM 7375 N ALA C 121 39.972 28.708 -73.936 1.00 30.91 N

ATOM 7376 CA ALA C 121 39.530 29.292 -75.170 1.00 30.91 C

ATOM 7377 CB ALA C 121 38.535 30.445 -74.956 1.00 30.91 C

ATOM 7378 C ALA C 121 40.699 29.847 -75.916 1.00 30.91 C

ATOM 7379 O ALA C 121 40.792 29.706 -77.134 1.00 30.91 O

ATOM 7380 N TRP C 122 41.626 30.505 -75.199 1.00 42.77 N

ATOM 7381 CA TRP C 122 42.730 31.122 -75.870 1.00 42.77 C

ATOM 7382 CB TRP C 122 43.642 31.918 -74.921 1.00 42.77 C

ATOM 7383 CG TRP C 122 44.665 32.774 -75.631 1.00 42.77 C

ATOM 7384 CD2 TRP C 122 45.945 32.313 -76.092 1.00 42.77 C

ATOM 7385 CD1 TRP C 122 44.584 34.092 -75.973 1.00 42.77 C

ATOM 7386 NE1 TRP C 122 45.733 34.483 -76.617 1.00 42.77 N

ATOM 7387 CE2 TRP C 122 46.580 33.398 -76.698 1.00 42.77 C

ATOM 7388 CE3 TRP C 122 46.542 31.088 -76.018 1.00 42.77 C

ATOM 7389 CZ2 TRP C 122 47.826 33.273 -77.241 1.00 42.77 C

ATOM 7390 CZ3 TRP C 122 47.800 30.965 -76.566 1.00 42.77 C

ATOM 7391 CH2 TRP C 122 48.429 32.037 -77.165 1.00 42.77 C

ATOM 7392 C TRP C 122 43.559 30.057 -76.516 1.00 42.77 C

ATOM 7393 O TRP C 122 43.919 30.162 -77.687 1.00 42.77 O

ATOM 7394 N MET C 123 43.866 28.983 -75.765 1.00100.87 N

ATOM 7395 CA MET C 123 44.721 27.941 -76.260 1.00100.87 C

ATOM 7396 CB MET C 123 44.970 26.848 -75.208 1.00100.87 C

ATOM 7397 CG MET C 123 45.876 25.713 -75.690 1.00100.87 C

ATOM 7398 SD MET C 123 46.168 24.419 -74.446 1.00100.87 S

ATOM 7399 CE MET C 123 47.075 23.314 -75.566 1.00100.87 C

ATOM 7400 C MET C 123 44.103 27.278 -77.451 1.00100.87 C

ATOM 7401 O MET C 123 44.770 27.052 -78.460 1.00100.87 O

ATOM 7402 N VAL C 124 42.802 26.946 -77.369 1.00102.71 N

ATOM 7403 CA VAL C 124 42.166 26.240 -78.444 1.00102.71 C

ATOM 7404 CB VAL C 124 40.769 25.798 -78.100 1.00102.71 C

ATOM 7405 CG1 VAL C 124 40.853 24.777 -76.954 1.00102.71 C

ATOM 7406 CG2 VAL C 124 39.917 27.033 -77.762 1.00102.71 C

ATOM 7407 C VAL C 124 42.091 27.094 -79.671 1.00102.71 C

ATOM 7408 O VAL C 124 42.339 26.622 -80.778 1.00102.71 O

ATOM 7409 N LEU C 125 41.690 28.367 -79.510 1.00 66.53 N

ATOM 7410 CA LEU C 125 41.520 29.271 -80.610 1.00 66.53 C

ATOM 7411 CB LEU C 125 40.709 30.509 -80.195 1.00 66.53 C

ATOM 7412 CG LEU C 125 39.324 30.119 -79.634 1.00 66.53 C

ATOM 7413 CD1 LEU C 125 38.461 31.353 -79.326 1.00 66.53 C

ATOM 7414 CD2 LEU C 125 38.616 29.101 -80.543 1.00 66.53 C

ATOM 7415 C LEU C 125 42.836 29.702 -81.176 1.00 66.53 C

ATOM 7416 O LEU C 125 42.993 29.788 -82.392 1.00 66.53 O

ATOM 7417 N SER C 126 43.820 29.984 -80.300 1.00 60.24 N

ATOM 7418 CA SER C 126 45.097 30.474 -80.735 1.00 60.24 C

ATOM 7419 CB SER C 126 45.982 30.957 -79.572 1.00 60.24 C

ATOM 7420 OG SER C 126 47.227 31.430 -80.065 1.00 60.24 O

ATOM 7421 C SER C 126 45.847 29.346 -81.436 1.00 60.24 C

ATOM 7422 O SER C 126 46.947 28.979 -80.941 1.00 60.24 O

ATOM 7423 OXT SER C 126 45.347 28.846 -82.478 1.00 60.24 O

TER 7423 SER C 126

ATOM 7425 N MET D 1 14.061 -1.809 -33.118 1.00 96.63 N

ATOM 7426 CA MET D 1 13.924 -0.352 -32.953 1.00 96.63 C

ATOM 7427 CB MET D 1 14.499 0.087 -31.594 1.00 96.63 C

ATOM 7428 CG MET D 1 13.723 -0.454 -30.391 1.00 96.63 C

ATOM 7429 SD MET D 1 12.049 0.228 -30.194 1.00 96.63 S

ATOM 7430 CE MET D 1 12.608 1.811 -29.501 1.00 96.63 C

ATOM 7431 C MET D 1 14.673 0.350 -34.024 1.00 96.63 C

ATOM 7432 O MET D 1 14.199 0.488 -35.152 1.00 96.63 O

ATOM 7433 N THR D 2 15.906 0.757 -33.691 1.00176.50 N

ATOM 7434 CA THR D 2 16.661 1.595 -34.558 1.00176.50 C

ATOM 7435 CB THR D 2 17.985 2.023 -33.977 1.00176.50 C

ATOM 7436 OG1 THR D 2 18.679 2.856 -34.895 1.00176.50 O

ATOM 7437 CG2 THR D 2 18.822 0.785 -33.607 1.00176.50 C

ATOM 7438 C THR D 2 16.877 0.993 -35.869 1.00176.50 C

ATOM 7439 O THR D 2 17.667 0.067 -35.983 1.00176.50 O

ATOM 7440 N PRO D 3 16.175 1.551 -36.835 1.00 96.91 N

ATOM 7441 CA PRO D 3 16.355 1.196 -38.215 1.00 96.91 C

ATOM 7442 CD PRO D 3 15.628 2.888 -36.688 1.00 96.91 C

ATOM 7443 CB PRO D 3 15.541 2.206 -39.014 1.00 96.91 C

ATOM 7444 CG PRO D 3 15.575 3.457 -38.117 1.00 96.91 C

ATOM 7445 C PRO D 3 17.805 1.467 -38.307 1.00 96.91 C

ATOM 7446 O PRO D 3 18.228 2.476 -37.745 1.00 96.91 O

ATOM 7447 N SER D 4 18.572 0.625 -39.014 1.00211.87 N

ATOM 7448 CA SER D 4 19.974 0.716 -38.778 1.00211.87 C

ATOM 7449 CB SER D 4 20.572 2.099 -39.106 1.00211.87 C

ATOM 7450 OG SER D 4 21.957 2.129 -38.803 1.00211.87 O

ATOM 7451 C SER D 4 20.041 0.470 -37.301 1.00211.87 C

ATOM 7452 O SER D 4 20.132 1.387 -36.485 1.00211.87 O

ATOM 7453 N THR D 5 20.020 -0.829 -36.951 1.00158.05 N

ATOM 7454 CA THR D 5 19.958 -1.362 -35.624 1.00158.05 C

ATOM 7455 CB THR D 5 19.903 -2.863 -35.588 1.00158.05 C

ATOM 7456 OG1 THR D 5 21.094 -3.412 -36.134 1.00158.05 O

ATOM 7457 CG2 THR D 5 18.686 -3.335 -36.402 1.00158.05 C

ATOM 7458 C THR D 5 21.176 -0.953 -34.882 1.00158.05 C

ATOM 7459 O THR D 5 21.859 0.000 -35.249 1.00158.05 O

ATOM 7460 N SER D 6 21.444 -1.665 -33.777 1.00 56.87 N

ATOM 7461 CA SER D 6 22.558 -1.345 -32.944 1.00 56.87 C

ATOM 7462 CB SER D 6 22.667 -2.267 -31.718 1.00 56.87 C

ATOM 7463 OG SER D 6 22.877 -3.608 -32.137 1.00 56.87 O

ATOM 7464 C SER D 6 23.805 -1.515 -33.746 1.00 56.87 C

ATOM 7465 O SER D 6 24.799 -0.841 -33.491 1.00 56.87 O

ATOM 7466 N ASP D 7 23.802 -2.444 -34.720 1.00173.82 N

ATOM 7467 CA ASP D 7 24.963 -2.626 -35.542 1.00173.82 C

ATOM 7468 CB ASP D 7 25.629 -4.001 -35.374 1.00173.82 C

ATOM 7469 CG ASP D 7 26.311 -4.024 -34.018 1.00173.82 C

ATOM 7470 OD1 ASP D 7 26.440 -2.929 -33.409 1.00173.82 O

ATOM 7471 OD2 ASP D 7 26.719 -5.131 -33.573 1.00173.82 O

ATOM 7472 C ASP D 7 24.516 -2.541 -36.961 1.00173.82 C

ATOM 7473 O ASP D 7 24.076 -3.528 -37.547 1.00173.82 O

ATOM 7474 N ALA D 8 24.628 -1.344 -37.556 1.00 76.39 N

ATOM 7475 CA ALA D 8 24.206 -1.154 -38.908 1.00 76.39 C

ATOM 7476 CB ALA D 8 24.010 0.319 -39.295 1.00 76.39 C

ATOM 7477 C ALA D 8 25.241 -1.690 -39.817 1.00 76.39 C

ATOM 7478 O ALA D 8 26.413 -1.801 -39.462 1.00 76.39 O

ATOM 7479 N ARG D 9 24.809 -1.973 -41.057 1.00204.20 N

ATOM 7480 CA ARG D 9 25.646 -2.668 -41.970 1.00204.20 C

ATOM 7481 CB ARG D 9 25.009 -3.980 -42.452 1.00204.20 C

ATOM 7482 CG ARG D 9 23.737 -3.732 -43.270 1.00204.20 C

ATOM 7483 CD ARG D 9 22.850 -4.961 -43.475 1.00204.20 C

ATOM 7484 NE ARG D 9 21.665 -4.799 -42.585 1.00204.20 N

ATOM 7485 CZ ARG D 9 21.370 -5.746 -41.648 1.00204.20 C

ATOM 7486 NH1 ARG D 9 22.156 -6.855 -41.526 1.00204.20 N

ATOM 7487 NH2 ARG D 9 20.286 -5.585 -40.834 1.00204.20 N

ATOM 7488 C ARG D 9 25.830 -1.892 -43.222 1.00204.20 C

ATOM 7489 O ARG D 9 25.711 -0.664 -43.288 1.00204.20 O

ATOM 7490 N SER D 10 26.204 -2.709 -44.225 1.00270.06 N

ATOM 7491 CA SER D 10 26.549 -2.449 -45.582 1.00270.06 C

ATOM 7492 CB SER D 10 26.603 -0.964 -45.960 1.00270.06 C

ATOM 7493 OG SER D 10 25.312 -0.384 -45.844 1.00270.06 O

ATOM 7494 C SER D 10 27.891 -3.095 -45.613 1.00270.06 C

ATOM 7495 O SER D 10 28.072 -4.091 -44.920 1.00270.06 O

ATOM 7496 N ARG D 11 28.865 -2.613 -46.403 1.00264.72 N

ATOM 7497 CA ARG D 11 30.154 -3.225 -46.240 1.00264.72 C

ATOM 7498 CB ARG D 11 31.143 -2.876 -47.367 1.00264.72 C

ATOM 7499 CG ARG D 11 32.550 -3.436 -47.142 1.00264.72 C

ATOM 7500 CD ARG D 11 33.548 -3.083 -48.248 1.00264.72 C

ATOM 7501 NE ARG D 11 33.178 -3.862 -49.463 1.00264.72 N

ATOM 7502 CZ ARG D 11 33.777 -3.584 -50.659 1.00264.72 C

ATOM 7503 NH1 ARG D 11 34.693 -2.576 -50.745 1.00264.72 N

ATOM 7504 NH2 ARG D 11 33.462 -4.314 -51.768 1.00264.72 N

ATOM 7505 C ARG D 11 30.723 -2.661 -44.968 1.00264.72 C

ATOM 7506 O ARG D 11 31.590 -1.790 -44.995 1.00264.72 O

ATOM 7507 N ARG D 12 30.229 -3.160 -43.811 1.00294.11 N

ATOM 7508 CA ARG D 12 30.607 -2.747 -42.485 1.00294.11 C

ATOM 7509 CB ARG D 12 32.127 -2.847 -42.256 1.00294.11 C

ATOM 7510 CG ARG D 12 32.588 -2.289 -40.910 1.00294.11 C

ATOM 7511 CD ARG D 12 34.090 -2.415 -40.661 1.00294.11 C

ATOM 7512 NE ARG D 12 34.414 -1.508 -39.526 1.00294.11 N

ATOM 7513 CZ ARG D 12 34.205 -1.901 -38.236 1.00294.11 C

ATOM 7514 NH1 ARG D 12 33.703 -3.142 -37.967 1.00294.11 N

ATOM 7515 NH2 ARG D 12 34.489 -1.041 -37.215 1.00294.11 N

ATOM 7516 C ARG D 12 30.169 -1.327 -42.269 1.00294.11 C

ATOM 7517 O ARG D 12 30.331 -0.767 -41.186 1.00294.11 O

ATOM 7518 N ARG D 13 29.530 -0.751 -43.297 1.00270.91 N

ATOM 7519 CA ARG D 13 29.098 0.599 -43.389 1.00270.91 C

ATOM 7520 CB ARG D 13 30.216 1.653 -43.284 1.00270.91 C

ATOM 7521 CG ARG D 13 30.947 1.785 -41.950 1.00270.91 C

ATOM 7522 CD ARG D 13 32.121 2.764 -42.031 1.00270.91 C

ATOM 7523 NE ARG D 13 32.761 2.838 -40.688 1.00270.91 N

ATOM 7524 CZ ARG D 13 33.989 3.418 -40.557 1.00270.91 C

ATOM 7525 NH1 ARG D 13 34.634 3.897 -41.660 1.00270.91 N

ATOM 7526 NH2 ARG D 13 34.570 3.526 -39.327 1.00270.91 N

ATOM 7527 C ARG D 13 28.756 0.688 -44.828 1.00270.91 C

ATOM 7528 O ARG D 13 29.185 -0.155 -45.608 1.00270.91 O

ATOM 7529 N SER D 14 28.023 1.723 -45.249 1.00 58.67 N

ATOM 7530 CA SER D 14 27.663 1.759 -46.631 1.00 58.67 C

ATOM 7531 CB SER D 14 26.667 2.877 -46.983 1.00 58.67 C

ATOM 7532 OG SER D 14 26.366 2.841 -48.370 1.00 58.67 O

ATOM 7533 C SER D 14 28.893 1.966 -47.467 1.00 58.67 C

ATOM 7534 O SER D 14 29.923 2.462 -47.014 1.00 58.67 O

ATOM 7535 N ALA D 15 28.796 1.474 -48.713 1.00 68.02 N

ATOM 7536 CA ALA D 15 29.729 1.538 -49.803 1.00 68.02 C

ATOM 7537 CB ALA D 15 29.371 0.578 -50.951 1.00 68.02 C

ATOM 7538 C ALA D 15 29.794 2.918 -50.384 1.00 68.02 C

ATOM 7539 O ALA D 15 30.777 3.284 -51.028 1.00 68.02 O

ATOM 7540 N GLU D 16 28.730 3.713 -50.177 1.00117.13 N

ATOM 7541 CA GLU D 16 28.500 4.962 -50.850 1.00117.13 C

ATOM 7542 CB GLU D 16 27.313 5.748 -50.270 1.00117.13 C

ATOM 7543 CG GLU D 16 25.954 5.158 -50.648 1.00117.13 C

ATOM 7544 CD GLU D 16 25.730 5.446 -52.127 1.00117.13 C

ATOM 7545 OE1 GLU D 16 26.246 4.663 -52.969 1.00117.13 O

ATOM 7546 OE2 GLU D 16 25.044 6.457 -52.433 1.00117.13 O

ATOM 7547 C GLU D 16 29.692 5.871 -50.842 1.00117.13 C

ATOM 7548 O GLU D 16 29.845 6.610 -51.815 1.00117.13 O

ATOM 7549 N PRO D 17 30.555 5.904 -49.867 1.00135.78 N

ATOM 7550 CA PRO D 17 31.647 6.833 -49.937 1.00135.78 C

ATOM 7551 CD PRO D 17 30.256 5.511 -48.499 1.00135.78 C

ATOM 7552 CB PRO D 17 32.401 6.668 -48.620 1.00135.78 C

ATOM 7553 CG PRO D 17 31.304 6.230 -47.631 1.00135.78 C

ATOM 7554 C PRO D 17 32.497 6.649 -51.160 1.00135.78 C

ATOM 7555 O PRO D 17 33.183 7.596 -51.541 1.00135.78 O

ATOM 7556 N PHE D 18 32.517 5.447 -51.766 1.00 51.46 N

ATOM 7557 CA PHE D 18 33.315 5.258 -52.942 1.00 51.46 C

ATOM 7558 CB PHE D 18 33.370 3.785 -53.377 1.00 51.46 C

ATOM 7559 CG PHE D 18 34.275 3.693 -54.555 1.00 51.46 C

ATOM 7560 CD1 PHE D 18 35.627 3.507 -54.381 1.00 51.46 C

ATOM 7561 CD2 PHE D 18 33.775 3.795 -55.832 1.00 51.46 C

ATOM 7562 CE1 PHE D 18 36.467 3.419 -55.465 1.00 51.46 C

ATOM 7563 CE2 PHE D 18 34.611 3.708 -56.920 1.00 51.46 C

ATOM 7564 CZ PHE D 18 35.960 3.519 -56.739 1.00 51.46 C

ATOM 7565 C PHE D 18 32.742 6.036 -54.083 1.00 51.46 C

ATOM 7566 O PHE D 18 33.437 6.828 -54.715 1.00 51.46 O

ATOM 7567 N LEU D 19 31.439 5.837 -54.362 1.00 86.15 N

ATOM 7568 CA LEU D 19 30.798 6.464 -55.485 1.00 86.15 C

ATOM 7569 CB LEU D 19 29.392 5.912 -55.762 1.00 86.15 C

ATOM 7570 CG LEU D 19 29.402 4.408 -56.092 1.00 86.15 C

ATOM 7571 CD1 LEU D 19 30.259 4.105 -57.333 1.00 86.15 C

ATOM 7572 CD2 LEU D 19 29.806 3.574 -54.867 1.00 86.15 C

ATOM 7573 C LEU D 19 30.686 7.932 -55.250 1.00 86.15 C

ATOM 7574 O LEU D 19 30.819 8.732 -56.176 1.00 86.15 O

ATOM 7575 N TRP D 20 30.425 8.318 -53.991 1.00 52.99 N

ATOM 7576 CA TRP D 20 30.245 9.698 -53.662 1.00 52.99 C

ATOM 7577 CB TRP D 20 29.896 9.913 -52.177 1.00 52.99 C

ATOM 7578 CG TRP D 20 29.595 11.347 -51.804 1.00 52.99 C

ATOM 7579 CD2 TRP D 20 30.463 12.183 -51.024 1.00 52.99 C

ATOM 7580 CD1 TRP D 20 28.487 12.093 -52.080 1.00 52.99 C

ATOM 7581 NE1 TRP D 20 28.612 13.345 -51.528 1.00 52.99 N

ATOM 7582 CE2 TRP D 20 29.823 13.413 -50.871 1.00 52.99 C

ATOM 7583 CE3 TRP D 20 31.690 11.945 -50.475 1.00 52.99 C

ATOM 7584 CZ2 TRP D 20 30.404 14.428 -50.166 1.00 52.99 C

ATOM 7585 CZ3 TRP D 20 32.274 12.972 -49.767 1.00 52.99 C

ATOM 7586 CH2 TRP D 20 31.643 14.189 -49.616 1.00 52.99 C

ATOM 7587 C TRP D 20 31.522 10.415 -53.968 1.00 52.99 C

ATOM 7588 O TRP D 20 31.504 11.509 -54.530 1.00 52.99 O

ATOM 7589 N LEU D 21 32.675 9.815 -53.618 1.00 45.52 N

ATOM 7590 CA LEU D 21 33.920 10.484 -53.863 1.00 45.52 C

ATOM 7591 CB LEU D 21 35.145 9.735 -53.313 1.00 45.52 C

ATOM 7592 CG LEU D 21 35.192 9.681 -51.774 1.00 45.52 C

ATOM 7593 CD1 LEU D 21 36.475 8.995 -51.280 1.00 45.52 C

ATOM 7594 CD2 LEU D 21 34.992 11.076 -51.159 1.00 45.52 C

ATOM 7595 C LEU D 21 34.131 10.682 -55.338 1.00 45.52 C

ATOM 7596 O LEU D 21 34.490 11.777 -55.768 1.00 45.52 O

ATOM 7597 N LEU D 22 33.895 9.649 -56.172 1.00 86.73 N

ATOM 7598 CA LEU D 22 34.146 9.848 -57.576 1.00 86.73 C

ATOM 7599 CB LEU D 22 33.920 8.599 -58.444 1.00 86.73 C

ATOM 7600 CG LEU D 22 35.038 7.550 -58.302 1.00 86.73 C

ATOM 7601 CD1 LEU D 22 35.099 6.965 -56.887 1.00 86.73 C

ATOM 7602 CD2 LEU D 22 34.932 6.477 -59.394 1.00 86.73 C

ATOM 7603 C LEU D 22 33.249 10.929 -58.081 1.00 86.73 C

ATOM 7604 O LEU D 22 33.688 11.842 -58.777 1.00 86.73 O

ATOM 7605 N PHE D 23 31.975 10.879 -57.665 1.00 83.33 N

ATOM 7606 CA PHE D 23 30.941 11.804 -58.021 1.00 83.33 C

ATOM 7607 CB PHE D 23 29.652 11.419 -57.266 1.00 83.33 C

ATOM 7608 CG PHE D 23 28.815 12.611 -56.960 1.00 83.33 C

ATOM 7609 CD1 PHE D 23 27.920 13.145 -57.856 1.00 83.33 C

ATOM 7610 CD2 PHE D 23 28.936 13.183 -55.715 1.00 83.33 C

ATOM 7611 CE1 PHE D 23 27.169 14.245 -57.505 1.00 83.33 C

ATOM 7612 CE2 PHE D 23 28.191 14.280 -55.360 1.00 83.33 C

ATOM 7613 CZ PHE D 23 27.303 14.814 -56.261 1.00 83.33 C

ATOM 7614 C PHE D 23 31.372 13.183 -57.623 1.00 83.33 C

ATOM 7615 O PHE D 23 31.090 14.155 -58.323 1.00 83.33 O

ATOM 7616 N SER D 24 32.042 13.313 -56.466 1.00 37.06 N

ATOM 7617 CA SER D 24 32.468 14.603 -56.003 1.00 37.06 C

ATOM 7618 CB SER D 24 33.113 14.543 -54.608 1.00 37.06 C

ATOM 7619 OG SER D 24 32.157 14.112 -53.651 1.00 37.06 O

ATOM 7620 C SER D 24 33.477 15.200 -56.939 1.00 37.06 C

ATOM 7621 O SER D 24 33.331 16.345 -57.368 1.00 37.06 O

ATOM 7622 N ALA D 25 34.534 14.435 -57.277 1.00 25.15 N

ATOM 7623 CA ALA D 25 35.577 14.934 -58.128 1.00 25.15 C

ATOM 7624 CB ALA D 25 36.722 13.926 -58.327 1.00 25.15 C

ATOM 7625 C ALA D 25 35.002 15.227 -59.475 1.00 25.15 C

ATOM 7626 O ALA D 25 35.327 16.237 -60.098 1.00 25.15 O

ATOM 7627 N GLY D 26 34.129 14.334 -59.968 1.00 21.31 N

ATOM 7628 CA GLY D 26 33.565 14.523 -61.272 1.00 21.31 C

ATOM 7629 C GLY D 26 32.721 15.761 -61.278 1.00 21.31 C

ATOM 7630 O GLY D 26 32.750 16.545 -62.226 1.00 21.31 O

ATOM 7631 N GLY D 27 31.936 15.968 -60.207 1.00 26.13 N

ATOM 7632 CA GLY D 27 31.057 17.099 -60.159 1.00 26.13 C

ATOM 7633 C GLY D 27 31.870 18.357 -60.201 1.00 26.13 C

ATOM 7634 O GLY D 27 31.461 19.349 -60.802 1.00 26.13 O

ATOM 7635 N MET D 28 33.022 18.368 -59.505 1.00 83.54 N

ATOM 7636 CA MET D 28 33.858 19.536 -59.481 1.00 83.54 C

ATOM 7637 CB MET D 28 35.036 19.398 -58.500 1.00 83.54 C

ATOM 7638 CG MET D 28 35.870 20.672 -58.339 1.00 83.54 C

ATOM 7639 SD MET D 28 35.066 21.995 -57.384 1.00 83.54 S

ATOM 7640 CE MET D 28 33.981 22.543 -58.731 1.00 83.54 C

ATOM 7641 C MET D 28 34.441 19.793 -60.840 1.00 83.54 C

ATOM 7642 O MET D 28 34.471 20.933 -61.302 1.00 83.54 O

ATOM 7643 N VAL D 29 34.920 18.734 -61.524 1.00 97.27 N

ATOM 7644 CA VAL D 29 35.578 18.917 -62.789 1.00 97.27 C

ATOM 7645 CB VAL D 29 36.193 17.659 -63.334 1.00 97.27 C

ATOM 7646 CG1 VAL D 29 37.239 17.171 -62.318 1.00 97.27 C

ATOM 7647 CG2 VAL D 29 35.092 16.636 -63.651 1.00 97.27 C

ATOM 7648 C VAL D 29 34.613 19.448 -63.802 1.00 97.27 C

ATOM 7649 O VAL D 29 34.930 20.375 -64.542 1.00 97.27 O

ATOM 7650 N THR D 30 33.395 18.885 -63.855 1.00124.34 N

ATOM 7651 CA THR D 30 32.420 19.304 -64.824 1.00124.34 C

ATOM 7652 CB THR D 30 31.194 18.444 -64.832 1.00124.34 C

ATOM 7653 OG1 THR D 30 30.330 18.843 -65.887 1.00124.34 O

ATOM 7654 CG2 THR D 30 30.485 18.586 -63.475 1.00124.34 C

ATOM 7655 C THR D 30 31.969 20.702 -64.529 1.00124.34 C

ATOM 7656 O THR D 30 31.460 21.395 -65.409 1.00124.34 O

ATOM 7657 N ALA D 31 32.017 21.118 -63.254 1.00 47.72 N

ATOM 7658 CA ALA D 31 31.588 22.451 -62.946 1.00 47.72 C

ATOM 7659 CB ALA D 31 31.512 22.712 -61.432 1.00 47.72 C

ATOM 7660 C ALA D 31 32.533 23.454 -63.538 1.00 47.72 C

ATOM 7661 O ALA D 31 32.110 24.447 -64.128 1.00 47.72 O

ATOM 7662 N LEU D 32 33.848 23.239 -63.345 1.00153.00 N

ATOM 7663 CA LEU D 32 34.842 24.159 -63.821 1.00153.00 C

ATOM 7664 CB LEU D 32 36.207 23.851 -63.177 1.00153.00 C

ATOM 7665 CG LEU D 32 37.308 24.894 -63.428 1.00153.00 C

ATOM 7666 CD1 LEU D 32 37.003 26.201 -62.681 1.00153.00 C

ATOM 7667 CD2 LEU D 32 38.697 24.329 -63.088 1.00153.00 C

ATOM 7668 C LEU D 32 35.027 24.107 -65.322 1.00153.00 C

ATOM 7669 O LEU D 32 34.975 25.138 -65.994 1.00153.00 O

ATOM 7670 N VAL D 33 35.323 22.896 -65.856 1.00146.67 N

ATOM 7671 CA VAL D 33 35.670 22.648 -67.242 1.00146.67 C

ATOM 7672 CB VAL D 33 36.620 21.493 -67.412 1.00146.67 C

ATOM 7673 CG1 VAL D 33 37.874 21.788 -66.574 1.00146.67 C

ATOM 7674 CG2 VAL D 33 35.913 20.166 -67.088 1.00146.67 C

ATOM 7675 C VAL D 33 34.544 22.432 -68.216 1.00146.67 C

ATOM 7676 O VAL D 33 34.669 22.771 -69.391 1.00146.67 O

ATOM 7677 N ALA D 34 33.425 21.829 -67.790 1.00 72.06 N

ATOM 7678 CA ALA D 34 32.438 21.391 -68.743 1.00 72.06 C

ATOM 7679 CB ALA D 34 31.283 20.640 -68.055 1.00 72.06 C

ATOM 7680 C ALA D 34 31.867 22.500 -69.592 1.00 72.06 C

ATOM 7681 O ALA D 34 31.770 22.300 -70.804 1.00 72.06 O

ATOM 7682 N PRO D 35 31.473 23.652 -69.112 1.00170.77 N

ATOM 7683 CA PRO D 35 30.875 24.638 -69.969 1.00170.77 C

ATOM 7684 CD PRO D 35 31.657 24.127 -67.748 1.00170.77 C

ATOM 7685 CB PRO D 35 30.444 25.786 -69.052 1.00170.77 C

ATOM 7686 CG PRO D 35 31.359 25.637 -67.823 1.00170.77 C

ATOM 7687 C PRO D 35 31.732 25.062 -71.129 1.00170.77 C

ATOM 7688 O PRO D 35 31.202 25.191 -72.232 1.00170.77 O

ATOM 7689 N VAL D 36 33.047 25.278 -70.934 1.00 99.06 N

ATOM 7690 CA VAL D 36 33.870 25.727 -72.024 1.00 99.06 C

ATOM 7691 CB VAL D 36 35.266 26.095 -71.613 1.00 99.06 C

ATOM 7692 CG1 VAL D 36 35.981 24.856 -71.050 1.00 99.06 C

ATOM 7693 CG2 VAL D 36 35.958 26.713 -72.840 1.00 99.06 C

ATOM 7694 C VAL D 36 33.968 24.667 -73.075 1.00 99.06 C

ATOM 7695 O VAL D 36 33.869 24.960 -74.264 1.00 99.06 O

ATOM 7696 N LEU D 37 34.156 23.398 -72.667 1.00 89.74 N

ATOM 7697 CA LEU D 37 34.330 22.339 -73.621 1.00 89.74 C

ATOM 7698 CB LEU D 37 34.688 20.982 -72.984 1.00 89.74 C

ATOM 7699 CG LEU D 37 36.132 20.903 -72.439 1.00 89.74 C

ATOM 7700 CD1 LEU D 37 37.160 20.978 -73.579 1.00 89.74 C

ATOM 7701 CD2 LEU D 37 36.402 21.951 -71.351 1.00 89.74 C

ATOM 7702 C LEU D 37 33.099 22.168 -74.452 1.00 89.74 C

ATOM 7703 O LEU D 37 33.196 21.914 -75.650 1.00 89.74 O

ATOM 7704 N LEU D 38 31.903 22.286 -73.850 1.00135.76 N

ATOM 7705 CA LEU D 38 30.711 22.097 -74.625 1.00135.76 C

ATOM 7706 CB LEU D 38 29.431 22.118 -73.764 1.00135.76 C

ATOM 7707 CG LEU D 38 28.164 21.567 -74.457 1.00135.76 C

ATOM 7708 CD1 LEU D 38 27.791 22.353 -75.715 1.00135.76 C

ATOM 7709 CD2 LEU D 38 28.305 20.068 -74.753 1.00135.76 C

ATOM 7710 C LEU D 38 30.660 23.210 -75.625 1.00135.76 C

ATOM 7711 O LEU D 38 30.228 23.019 -76.758 1.00135.76 O

ATOM 7712 N LEU D 39 31.103 24.419 -75.231 1.00113.93 N

ATOM 7713 CA LEU D 39 31.026 25.518 -76.147 1.00113.93 C

ATOM 7714 CB LEU D 39 31.501 26.843 -75.521 1.00113.93 C

ATOM 7715 CG LEU D 39 31.145 28.123 -76.318 1.00113.93 C

ATOM 7716 CD1 LEU D 39 31.674 29.363 -75.592 1.00113.93 C

ATOM 7717 CD2 LEU D 39 31.587 28.103 -77.792 1.00113.93 C

ATOM 7718 C LEU D 39 31.892 25.224 -77.340 1.00113.93 C

ATOM 7719 O LEU D 39 31.430 25.315 -78.478 1.00113.93 O

ATOM 7720 N LEU D 40 33.170 24.848 -77.121 1.00115.58 N

ATOM 7721 CA LEU D 40 34.019 24.643 -78.258 1.00115.58 C

ATOM 7722 CB LEU D 40 35.486 24.342 -77.885 1.00115.58 C

ATOM 7723 CG LEU D 40 36.309 25.568 -77.424 1.00115.58 C

ATOM 7724 CD1 LEU D 40 36.555 26.544 -78.586 1.00115.58 C

ATOM 7725 CD2 LEU D 40 35.692 26.266 -76.205 1.00115.58 C

ATOM 7726 C LEU D 40 33.519 23.507 -79.099 1.00115.58 C

ATOM 7727 O LEU D 40 33.216 23.694 -80.276 1.00115.58 O

ATOM 7728 N PHE D 41 33.384 22.301 -78.513 1.00130.59 N

ATOM 7729 CA PHE D 41 32.978 21.171 -79.302 1.00130.59 C

ATOM 7730 CB PHE D 41 33.075 19.827 -78.566 1.00130.59 C

ATOM 7731 CG PHE D 41 32.197 18.955 -79.390 1.00130.59 C

ATOM 7732 CD1 PHE D 41 32.555 18.612 -80.671 1.00130.59 C

ATOM 7733 CD2 PHE D 41 31.005 18.493 -78.880 1.00130.59 C

ATOM 7734 CE1 PHE D 41 31.728 17.817 -81.424 1.00130.59 C

ATOM 7735 CE2 PHE D 41 30.177 17.695 -79.631 1.00130.59 C

ATOM 7736 CZ PHE D 41 30.539 17.358 -80.912 1.00130.59 C

ATOM 7737 C PHE D 41 31.570 21.255 -79.808 1.00130.59 C

ATOM 7738 O PHE D 41 31.326 21.144 -81.007 1.00130.59 O

ATOM 7739 N GLY D 42 30.595 21.439 -78.902 1.00 71.79 N

ATOM 7740 CA GLY D 42 29.218 21.393 -79.301 1.00 71.79 C

ATOM 7741 C GLY D 42 28.808 22.541 -80.178 1.00 71.79 C

ATOM 7742 O GLY D 42 27.969 22.348 -81.053 1.00 71.79 O

ATOM 7743 N LEU D 43 29.189 23.789 -79.820 1.00145.74 N

ATOM 7744 CA LEU D 43 28.853 24.949 -80.610 1.00145.74 C

ATOM 7745 CB LEU D 43 28.561 26.180 -79.736 1.00145.74 C

ATOM 7746 CG LEU D 43 27.311 26.011 -78.854 1.00145.74 C

ATOM 7747 CD1 LEU D 43 27.491 24.873 -77.838 1.00145.74 C

ATOM 7748 CD2 LEU D 43 26.910 27.336 -78.187 1.00145.74 C

ATOM 7749 C LEU D 43 29.847 25.407 -81.642 1.00145.74 C

ATOM 7750 O LEU D 43 29.540 25.503 -82.829 1.00145.74 O

ATOM 7751 N ALA D 44 31.084 25.710 -81.184 1.00 61.31 N

ATOM 7752 CA ALA D 44 32.064 26.387 -81.996 1.00 61.31 C

ATOM 7753 CB ALA D 44 33.322 26.785 -81.203 1.00 61.31 C

ATOM 7754 C ALA D 44 32.517 25.552 -83.141 1.00 61.31 C

ATOM 7755 O ALA D 44 32.554 26.022 -84.278 1.00 61.31 O

ATOM 7756 N PHE D 45 32.854 24.280 -82.869 1.00115.72 N

ATOM 7757 CA PHE D 45 33.378 23.438 -83.902 1.00115.72 C

ATOM 7758 CB PHE D 45 33.711 22.010 -83.416 1.00115.72 C

ATOM 7759 CG PHE D 45 35.060 21.942 -82.770 1.00115.72 C

ATOM 7760 CD1 PHE D 45 35.341 22.588 -81.590 1.00115.72 C

ATOM 7761 CD2 PHE D 45 36.050 21.172 -83.340 1.00115.72 C

ATOM 7762 CE1 PHE D 45 36.584 22.498 -81.007 1.00115.72 C

ATOM 7763 CE2 PHE D 45 37.296 21.073 -82.765 1.00115.72 C

ATOM 7764 CZ PHE D 45 37.567 21.740 -81.596 1.00115.72 C

ATOM 7765 C PHE D 45 32.396 23.296 -85.022 1.00115.72 C

ATOM 7766 O PHE D 45 32.728 23.623 -86.156 1.00115.72 O

ATOM 7767 N PRO D 46 31.194 22.857 -84.799 1.00 82.37 N

ATOM 7768 CA PRO D 46 30.250 22.666 -85.864 1.00 82.37 C

ATOM 7769 CD PRO D 46 30.679 22.465 -83.500 1.00 82.37 C

ATOM 7770 CB PRO D 46 29.037 21.993 -85.217 1.00 82.37 C

ATOM 7771 CG PRO D 46 29.162 22.341 -83.721 1.00 82.37 C

ATOM 7772 C PRO D 46 29.915 23.948 -86.559 1.00 82.37 C

ATOM 7773 O PRO D 46 29.525 23.892 -87.723 1.00 82.37 O

ATOM 7774 N LEU D 47 29.972 25.089 -85.844 1.00 75.73 N

ATOM 7775 CA LEU D 47 29.668 26.389 -86.376 1.00 75.73 C

ATOM 7776 CB LEU D 47 29.338 27.422 -85.285 1.00 75.73 C

ATOM 7777 CG LEU D 47 28.042 27.070 -84.528 1.00 75.73 C

ATOM 7778 CD1 LEU D 47 27.640 28.180 -83.544 1.00 75.73 C

ATOM 7779 CD2 LEU D 47 26.913 26.697 -85.505 1.00 75.73 C

ATOM 7780 C LEU D 47 30.759 26.936 -87.254 1.00 75.73 C

ATOM 7781 O LEU D 47 30.472 27.609 -88.243 1.00 75.73 O

ATOM 7782 N GLY D 48 32.042 26.661 -86.939 1.00 88.68 N

ATOM 7783 CA GLY D 48 33.119 27.258 -87.687 1.00 88.68 C

ATOM 7784 C GLY D 48 34.062 27.910 -86.711 1.00 88.68 C

ATOM 7785 O GLY D 48 33.654 28.458 -85.690 1.00 88.68 O

ATOM 7786 N TRP D 49 35.370 27.872 -87.049 1.00168.36 N

ATOM 7787 CA TRP D 49 36.470 28.243 -86.200 1.00168.36 C

ATOM 7788 CB TRP D 49 37.074 26.964 -85.620 1.00168.36 C

ATOM 7789 CG TRP D 49 37.407 25.964 -86.718 1.00168.36 C

ATOM 7790 CD2 TRP D 49 36.440 25.179 -87.442 1.00168.36 C

ATOM 7791 CD1 TRP D 49 38.618 25.656 -87.263 1.00168.36 C

ATOM 7792 NE1 TRP D 49 38.472 24.734 -88.268 1.00168.36 N

ATOM 7793 CE2 TRP D 49 37.137 24.433 -88.394 1.00168.36 C

ATOM 7794 CE3 TRP D 49 35.082 25.084 -87.331 1.00168.36 C

ATOM 7795 CZ2 TRP D 49 36.490 23.584 -89.246 1.00168.36 C

ATOM 7796 CZ3 TRP D 49 34.437 24.231 -88.197 1.00168.36 C

ATOM 7797 CH2 TRP D 49 35.122 23.493 -89.137 1.00168.36 C

ATOM 7798 C TRP D 49 37.526 28.838 -87.085 1.00168.36 C

ATOM 7799 O TRP D 49 37.292 29.061 -88.272 1.00168.36 O

ATOM 7800 N LEU D 50 38.720 29.136 -86.519 1.00163.79 N

ATOM 7801 CA LEU D 50 39.773 29.678 -87.334 1.00163.79 C

ATOM 7802 CB LEU D 50 40.389 30.954 -86.736 1.00163.79 C

ATOM 7803 CG LEU D 50 39.389 32.122 -86.616 1.00163.79 C

ATOM 7804 CD1 LEU D 50 38.901 32.583 -87.999 1.00163.79 C

ATOM 7805 CD2 LEU D 50 38.235 31.784 -85.658 1.00163.79 C

ATOM 7806 C LEU D 50 40.873 28.651 -87.446 1.00163.79 C

ATOM 7807 O LEU D 50 41.986 28.864 -86.969 1.00163.79 O

ATOM 7808 N ASP D 51 40.563 27.509 -88.094 1.00199.19 N

ATOM 7809 CA ASP D 51 41.436 26.402 -88.409 1.00199.19 C

ATOM 7810 CB ASP D 51 42.652 26.818 -89.254 1.00199.19 C

ATOM 7811 CG ASP D 51 42.161 27.157 -90.653 1.00199.19 C

ATOM 7812 OD1 ASP D 51 41.077 26.643 -91.039 1.00199.19 O

ATOM 7813 OD2 ASP D 51 42.861 27.935 -91.355 1.00199.19 O

ATOM 7814 C ASP D 51 41.952 25.739 -87.170 1.00199.19 C

ATOM 7815 O ASP D 51 42.633 24.717 -87.252 1.00199.19 O

ATOM 7816 N ALA D 52 41.631 26.269 -85.978 1.00 72.31 N

ATOM 7817 CA ALA D 52 42.134 25.606 -84.811 1.00 72.31 C

ATOM 7818 CB ALA D 52 42.044 26.445 -83.515 1.00 72.31 C

ATOM 7819 C ALA D 52 41.431 24.282 -84.654 1.00 72.31 C

ATOM 7820 O ALA D 52 42.055 23.279 -84.319 1.00 72.31 O

ATOM 7821 N PRO D 53 40.140 24.268 -84.878 1.00179.28 N

ATOM 7822 CA PRO D 53 39.366 23.049 -84.784 1.00179.28 C

ATOM 7823 CD PRO D 53 39.363 25.387 -84.354 1.00179.28 C

ATOM 7824 CB PRO D 53 37.926 23.466 -84.504 1.00179.28 C

ATOM 7825 CG PRO D 53 38.076 24.797 -83.767 1.00179.28 C

ATOM 7826 C PRO D 53 39.453 22.072 -85.918 1.00179.28 C

ATOM 7827 O PRO D 53 38.618 21.167 -85.948 1.00179.28 O

ATOM 7828 N ASP D 54 40.383 22.237 -86.876 1.00 81.22 N

ATOM 7829 CA ASP D 54 40.436 21.336 -87.995 1.00 81.22 C

ATOM 7830 CB ASP D 54 41.435 21.781 -89.079 1.00 81.22 C

ATOM 7831 CG ASP D 54 41.185 20.969 -90.342 1.00 81.22 C

ATOM 7832 OD1 ASP D 54 40.072 20.392 -90.472 1.00 81.22 O

ATOM 7833 OD2 ASP D 54 42.108 20.915 -91.198 1.00 81.22 O

ATOM 7834 C ASP D 54 40.856 19.979 -87.513 1.00 81.22 C

ATOM 7835 O ASP D 54 41.401 19.829 -86.421 1.00 81.22 O

ATOM 7836 N HIS D 55 40.613 18.952 -88.354 1.00 74.81 N

ATOM 7837 CA HIS D 55 40.888 17.583 -88.028 1.00 74.81 C

ATOM 7838 ND1 HIS D 55 40.231 14.285 -88.302 1.00 74.81 N

ATOM 7839 CG HIS D 55 41.014 15.220 -88.940 1.00 74.81 C

ATOM 7840 CB HIS D 55 40.606 16.637 -89.207 1.00 74.81 C

ATOM 7841 NE2 HIS D 55 42.138 13.266 -88.822 1.00 74.81 N

ATOM 7842 CD2 HIS D 55 42.176 14.581 -89.250 1.00 74.81 C

ATOM 7843 CE1 HIS D 55 40.951 13.135 -88.258 1.00 74.81 C

ATOM 7844 C HIS D 55 42.335 17.432 -87.694 1.00 74.81 C

ATOM 7845 O HIS D 55 42.681 16.823 -86.684 1.00 74.81 O

ATOM 7846 N GLY D 56 43.225 18.012 -88.514 1.00 21.11 N

ATOM 7847 CA GLY D 56 44.626 17.816 -88.292 1.00 21.11 C

ATOM 7848 C GLY D 56 45.012 18.353 -86.950 1.00 21.11 C

ATOM 7849 O GLY D 56 45.806 17.740 -86.239 1.00 21.11 O

ATOM 7850 N HIS D 57 44.481 19.529 -86.576 1.00 76.06 N

ATOM 7851 CA HIS D 57 44.871 20.136 -85.335 1.00 76.06 C

ATOM 7852 ND1 HIS D 57 44.664 22.215 -82.728 1.00 76.06 N

ATOM 7853 CG HIS D 57 45.041 22.300 -84.050 1.00 76.06 C

ATOM 7854 CB HIS D 57 44.359 21.578 -85.175 1.00 76.06 C

ATOM 7855 NE2 HIS D 57 46.425 23.571 -82.800 1.00 76.06 N

ATOM 7856 CD2 HIS D 57 46.118 23.132 -84.076 1.00 76.06 C

ATOM 7857 CE1 HIS D 57 45.525 22.994 -82.025 1.00 76.06 C

ATOM 7858 C HIS D 57 44.381 19.346 -84.161 1.00 76.06 C

ATOM 7859 O HIS D 57 45.105 19.175 -83.182 1.00 76.06 O

ATOM 7860 N LEU D 58 43.128 18.853 -84.215 1.00 95.86 N

ATOM 7861 CA LEU D 58 42.551 18.152 -83.099 1.00 95.86 C

ATOM 7862 CB LEU D 58 41.073 17.785 -83.354 1.00 95.86 C

ATOM 7863 CG LEU D 58 40.264 17.261 -82.141 1.00 95.86 C

ATOM 7864 CD1 LEU D 58 38.821 16.942 -82.560 1.00 95.86 C

ATOM 7865 CD2 LEU D 58 40.924 16.066 -81.433 1.00 95.86 C

ATOM 7866 C LEU D 58 43.335 16.897 -82.886 1.00 95.86 C

ATOM 7867 O LEU D 58 43.663 16.540 -81.756 1.00 95.86 O

ATOM 7868 N LEU D 59 43.667 16.203 -83.986 1.00 38.84 N

ATOM 7869 CA LEU D 59 44.365 14.954 -83.910 1.00 38.84 C

ATOM 7870 CB LEU D 59 44.585 14.337 -85.301 1.00 38.84 C

ATOM 7871 CG LEU D 59 45.249 12.949 -85.283 1.00 38.84 C

ATOM 7872 CD1 LEU D 59 44.339 11.908 -84.610 1.00 38.84 C

ATOM 7873 CD2 LEU D 59 45.687 12.525 -86.694 1.00 38.84 C

ATOM 7874 C LEU D 59 45.698 15.214 -83.297 1.00 38.84 C

ATOM 7875 O LEU D 59 46.190 14.428 -82.489 1.00 38.84 O

ATOM 7876 N ALA D 60 46.324 16.344 -83.665 1.00 26.29 N

ATOM 7877 CA ALA D 60 47.616 16.635 -83.132 1.00 26.29 C

ATOM 7878 CB ALA D 60 48.186 17.968 -83.646 1.00 26.29 C

ATOM 7879 C ALA D 60 47.479 16.742 -81.650 1.00 26.29 C

ATOM 7880 O ALA D 60 48.309 16.217 -80.910 1.00 26.29 O

ATOM 7881 N MET D 61 46.423 17.434 -81.179 1.00121.79 N

ATOM 7882 CA MET D 61 46.242 17.621 -79.770 1.00121.79 C

ATOM 7883 CB MET D 61 45.056 18.550 -79.458 1.00121.79 C

ATOM 7884 CG MET D 61 45.269 19.982 -79.956 1.00121.79 C

ATOM 7885 SD MET D 61 43.869 21.107 -79.673 1.00121.79 S

ATOM 7886 CE MET D 61 42.869 20.479 -81.053 1.00121.79 C

ATOM 7887 C MET D 61 45.996 16.309 -79.087 1.00121.79 C

ATOM 7888 O MET D 61 46.753 15.924 -78.200 1.00121.79 O

ATOM 7889 N VAL D 62 45.014 15.528 -79.580 1.00103.47 N

ATOM 7890 CA VAL D 62 44.561 14.324 -78.932 1.00103.47 C

ATOM 7891 CB VAL D 62 43.499 13.613 -79.718 1.00103.47 C

ATOM 7892 CG1 VAL D 62 44.113 13.127 -81.043 1.00103.47 C

ATOM 7893 CG2 VAL D 62 42.919 12.482 -78.853 1.00103.47 C

ATOM 7894 C VAL D 62 45.710 13.379 -78.777 1.00103.47 C

ATOM 7895 O VAL D 62 45.772 12.619 -77.812 1.00103.47 O

ATOM 7896 N ARG D 63 46.631 13.378 -79.753 1.00106.00 N

ATOM 7897 CA ARG D 63 47.771 12.505 -79.745 1.00106.00 C

ATOM 7898 CB ARG D 63 48.550 12.523 -81.071 1.00106.00 C

ATOM 7899 CG ARG D 63 49.573 11.390 -81.182 1.00106.00 C

ATOM 7900 CD ARG D 63 50.364 11.381 -82.493 1.00106.00 C

ATOM 7901 NE ARG D 63 51.301 10.223 -82.426 1.00106.00 N

ATOM 7902 CZ ARG D 63 52.304 10.089 -83.343 1.00106.00 C

ATOM 7903 NH1 ARG D 63 52.460 11.020 -84.329 1.00106.00 N

ATOM 7904 NH2 ARG D 63 53.154 9.024 -83.270 1.00106.00 N

ATOM 7905 C ARG D 63 48.729 12.872 -78.644 1.00106.00 C

ATOM 7906 O ARG D 63 49.473 12.017 -78.169 1.00106.00 O

ATOM 7907 N ASN D 64 48.780 14.161 -78.247 1.00 95.59 N

ATOM 7908 CA ASN D 64 49.693 14.604 -77.224 1.00 95.59 C

ATOM 7909 CB ASN D 64 49.651 16.128 -77.009 1.00 95.59 C

ATOM 7910 CG ASN D 64 50.758 16.522 -76.044 1.00 95.59 C

ATOM 7911 OD1 ASN D 64 50.539 16.638 -74.840 1.00 95.59 O

ATOM 7912 ND2 ASN D 64 51.988 16.732 -76.585 1.00 95.59 N

ATOM 7913 C ASN D 64 49.318 13.941 -75.936 1.00 95.59 C

ATOM 7914 O ASN D 64 48.161 13.585 -75.718 1.00 95.59 O

ATOM 7915 N PRO D 65 50.288 13.719 -75.086 1.00 79.27 N

ATOM 7916 CA PRO D 65 50.053 13.086 -73.819 1.00 79.27 C

ATOM 7917 CD PRO D 65 51.686 13.687 -75.471 1.00 79.27 C

ATOM 7918 CB PRO D 65 51.435 12.791 -73.230 1.00 79.27 C

ATOM 7919 CG PRO D 65 52.419 13.572 -74.125 1.00 79.27 C

ATOM 7920 C PRO D 65 49.163 13.907 -72.949 1.00 79.27 C

ATOM 7921 O PRO D 65 48.304 13.349 -72.267 1.00 79.27 O

ATOM 7922 N ILE D 66 49.339 15.237 -72.981 1.00129.60 N

ATOM 7923 CA ILE D 66 48.558 16.124 -72.180 1.00129.60 C

ATOM 7924 CB ILE D 66 48.947 17.552 -72.405 1.00129.60 C

ATOM 7925 CG1 ILE D 66 50.410 17.787 -72.000 1.00129.60 C

ATOM 7926 CG2 ILE D 66 47.931 18.433 -71.671 1.00129.60 C

ATOM 7927 CD1 ILE D 66 50.695 17.480 -70.531 1.00129.60 C

ATOM 7928 C ILE D 66 47.140 15.998 -72.621 1.00129.60 C

ATOM 7929 O ILE D 66 46.232 15.886 -71.801 1.00129.60 O

ATOM 7930 N THR D 67 46.916 16.006 -73.948 1.00 50.69 N

ATOM 7931 CA THR D 67 45.571 15.943 -74.430 1.00 50.69 C

ATOM 7932 CB THR D 67 45.391 16.219 -75.881 1.00 50.69 C

ATOM 7933 OG1 THR D 67 45.881 17.513 -76.203 1.00 50.69 O

ATOM 7934 CG2 THR D 67 43.891 16.127 -76.209 1.00 50.69 C

ATOM 7935 C THR D 67 44.981 14.608 -74.137 1.00 50.69 C

ATOM 7936 O THR D 67 43.778 14.500 -73.918 1.00 50.69 O

ATOM 7937 N LYS D 68 45.788 13.535 -74.162 1.00135.59 N

ATOM 7938 CA LYS D 68 45.192 12.260 -73.899 1.00135.59 C

ATOM 7939 CB LYS D 68 46.106 11.070 -74.238 1.00135.59 C

ATOM 7940 CG LYS D 68 47.491 11.038 -73.597 1.00135.59 C

ATOM 7941 CD LYS D 68 48.258 9.789 -74.042 1.00135.59 C

ATOM 7942 CE LYS D 68 48.197 9.584 -75.563 1.00135.59 C

ATOM 7943 NZ LYS D 68 48.812 8.294 -75.953 1.00135.59 N

ATOM 7944 C LYS D 68 44.692 12.219 -72.490 1.00135.59 C

ATOM 7945 O LYS D 68 43.605 11.703 -72.236 1.00135.59 O

ATOM 7946 N LEU D 69 45.455 12.780 -71.530 1.00134.94 N

ATOM 7947 CA LEU D 69 45.005 12.781 -70.167 1.00134.94 C

ATOM 7948 CB LEU D 69 46.001 13.429 -69.190 1.00134.94 C

ATOM 7949 CG LEU D 69 47.322 12.662 -69.018 1.00134.94 C

ATOM 7950 CD1 LEU D 69 48.229 13.363 -67.993 1.00134.94 C

ATOM 7951 CD2 LEU D 69 47.073 11.183 -68.681 1.00134.94 C

ATOM 7952 C LEU D 69 43.761 13.606 -70.087 1.00134.94 C

ATOM 7953 O LEU D 69 42.785 13.218 -69.446 1.00134.94 O

ATOM 7954 N VAL D 70 43.764 14.770 -70.761 1.00 30.35 N

ATOM 7955 CA VAL D 70 42.668 15.692 -70.689 1.00 30.35 C

ATOM 7956 CB VAL D 70 42.918 16.941 -71.484 1.00 30.35 C

ATOM 7957 CG1 VAL D 70 41.652 17.813 -71.442 1.00 30.35 C

ATOM 7958 CG2 VAL D 70 44.176 17.634 -70.933 1.00 30.35 C

ATOM 7959 C VAL D 70 41.424 15.065 -71.233 1.00 30.35 C

ATOM 7960 O VAL D 70 40.357 15.165 -70.630 1.00 30.35 O

ATOM 7961 N VAL D 71 41.526 14.388 -72.389 1.00 88.54 N

ATOM 7962 CA VAL D 71 40.356 13.828 -72.999 1.00 88.54 C

ATOM 7963 CB VAL D 71 40.644 13.181 -74.323 1.00 88.54 C

ATOM 7964 CG1 VAL D 71 41.600 11.997 -74.100 1.00 88.54 C

ATOM 7965 CG2 VAL D 71 39.307 12.789 -74.971 1.00 88.54 C

ATOM 7966 C VAL D 71 39.766 12.787 -72.100 1.00 88.54 C

ATOM 7967 O VAL D 71 38.546 12.689 -71.975 1.00 88.54 O

ATOM 7968 N LEU D 72 40.613 11.965 -71.452 1.00 52.03 N

ATOM 7969 CA LEU D 72 40.067 10.912 -70.647 1.00 52.03 C

ATOM 7970 CB LEU D 72 41.143 9.966 -70.083 1.00 52.03 C

ATOM 7971 CG LEU D 72 40.564 8.743 -69.341 1.00 52.03 C

ATOM 7972 CD1 LEU D 72 39.694 7.885 -70.276 1.00 52.03 C

ATOM 7973 CD2 LEU D 72 41.673 7.918 -68.666 1.00 52.03 C

ATOM 7974 C LEU D 72 39.290 11.484 -69.495 1.00 52.03 C

ATOM 7975 O LEU D 72 38.139 11.116 -69.267 1.00 52.03 O

ATOM 7976 N VAL D 73 39.887 12.444 -68.767 1.00 38.44 N

ATOM 7977 CA VAL D 73 39.297 13.016 -67.586 1.00 38.44 C

ATOM 7978 CB VAL D 73 40.229 13.985 -66.918 1.00 38.44 C

ATOM 7979 CG1 VAL D 73 39.503 14.639 -65.734 1.00 38.44 C

ATOM 7980 CG2 VAL D 73 41.513 13.235 -66.522 1.00 38.44 C

ATOM 7981 C VAL D 73 38.038 13.747 -67.940 1.00 38.44 C

ATOM 7982 O VAL D 73 37.069 13.740 -67.180 1.00 38.44 O

ATOM 7983 N LEU D 74 38.026 14.427 -69.099 1.00154.44 N

ATOM 7984 CA LEU D 74 36.871 15.182 -69.485 1.00154.44 C

ATOM 7985 CB LEU D 74 37.056 16.015 -70.765 1.00154.44 C

ATOM 7986 CG LEU D 74 37.997 17.221 -70.600 1.00154.44 C

ATOM 7987 CD1 LEU D 74 37.990 18.097 -71.861 1.00154.44 C

ATOM 7988 CD2 LEU D 74 37.667 18.027 -69.332 1.00154.44 C

ATOM 7989 C LEU D 74 35.719 14.263 -69.726 1.00154.44 C

ATOM 7990 O LEU D 74 34.570 14.634 -69.496 1.00154.44 O

ATOM 7991 N VAL D 75 35.988 13.088 -70.319 1.00 60.67 N

ATOM 7992 CA VAL D 75 34.948 12.134 -70.583 1.00 60.67 C

ATOM 7993 CB VAL D 75 35.367 11.107 -71.590 1.00 60.67 C

ATOM 7994 CG1 VAL D 75 34.247 10.064 -71.743 1.00 60.67 C

ATOM 7995 CG2 VAL D 75 35.703 11.844 -72.896 1.00 60.67 C

ATOM 7996 C VAL D 75 34.505 11.400 -69.345 1.00 60.67 C

ATOM 7997 O VAL D 75 33.311 11.325 -69.060 1.00 60.67 O

ATOM 7998 N VAL D 76 35.478 10.822 -68.603 1.00132.47 N

ATOM 7999 CA VAL D 76 35.245 9.938 -67.484 1.00132.47 C

ATOM 8000 CB VAL D 76 36.451 9.114 -67.123 1.00132.47 C

ATOM 8001 CG1 VAL D 76 36.753 8.175 -68.298 1.00132.47 C

ATOM 8002 CG2 VAL D 76 37.625 10.039 -66.773 1.00132.47 C

ATOM 8003 C VAL D 76 34.727 10.563 -66.219 1.00132.47 C

ATOM 8004 O VAL D 76 33.781 10.047 -65.627 1.00132.47 O

ATOM 8005 N LEU D 77 35.304 11.688 -65.757 1.00 54.50 N

ATOM 8006 CA LEU D 77 34.901 12.192 -64.470 1.00 54.50 C

ATOM 8007 CB LEU D 77 35.673 13.453 -64.043 1.00 54.50 C

ATOM 8008 CG LEU D 77 37.174 13.199 -63.810 1.00 54.50 C

ATOM 8009 CD1 LEU D 77 37.876 14.459 -63.284 1.00 54.50 C

ATOM 8010 CD2 LEU D 77 37.404 11.975 -62.909 1.00 54.50 C

ATOM 8011 C LEU D 77 33.445 12.529 -64.501 1.00 54.50 C

ATOM 8012 O LEU D 77 32.704 12.224 -63.570 1.00 54.50 O

ATOM 8013 N ALA D 78 32.979 13.146 -65.593 1.00 42.01 N

ATOM 8014 CA ALA D 78 31.604 13.541 -65.675 1.00 42.01 C

ATOM 8015 CB ALA D 78 31.280 14.271 -66.989 1.00 42.01 C

ATOM 8016 C ALA D 78 30.717 12.334 -65.597 1.00 42.01 C

ATOM 8017 O ALA D 78 29.621 12.405 -65.041 1.00 42.01 O

ATOM 8018 N LEU D 79 31.149 11.201 -66.179 1.00100.01 N

ATOM 8019 CA LEU D 79 30.315 10.030 -66.233 1.00100.01 C

ATOM 8020 CB LEU D 79 30.953 8.912 -67.077 1.00100.01 C

ATOM 8021 CG LEU D 79 29.957 7.837 -67.569 1.00100.01 C

ATOM 8022 CD1 LEU D 79 30.681 6.746 -68.371 1.00100.01 C

ATOM 8023 CD2 LEU D 79 29.056 7.275 -66.459 1.00100.01 C

ATOM 8024 C LEU D 79 30.054 9.519 -64.836 1.00100.01 C

ATOM 8025 O LEU D 79 28.920 9.208 -64.472 1.00100.01 O

ATOM 8026 N PHE D 80 31.094 9.465 -63.988 1.00 83.21 N

ATOM 8027 CA PHE D 80 30.964 8.957 -62.651 1.00 83.21 C

ATOM 8028 CB PHE D 80 32.312 8.814 -61.923 1.00 83.21 C

ATOM 8029 CG PHE D 80 32.963 7.641 -62.569 1.00 83.21 C

ATOM 8030 CD1 PHE D 80 33.614 7.774 -63.774 1.00 83.21 C

ATOM 8031 CD2 PHE D 80 32.911 6.403 -61.969 1.00 83.21 C

ATOM 8032 CE1 PHE D 80 34.207 6.685 -64.369 1.00 83.21 C

ATOM 8033 CE2 PHE D 80 33.502 5.312 -62.560 1.00 83.21 C

ATOM 8034 CZ PHE D 80 34.151 5.453 -63.763 1.00 83.21 C

ATOM 8035 C PHE D 80 30.041 9.844 -61.867 1.00 83.21 C

ATOM 8036 O PHE D 80 29.534 9.452 -60.819 1.00 83.21 O

ATOM 8037 N HIS D 81 29.941 11.125 -62.252 1.00 77.88 N

ATOM 8038 CA HIS D 81 29.040 12.047 -61.610 1.00 77.88 C

ATOM 8039 ND1 HIS D 81 29.038 15.023 -59.988 1.00 77.88 N

ATOM 8040 CG HIS D 81 28.711 14.595 -61.256 1.00 77.88 C

ATOM 8041 CB HIS D 81 29.426 13.494 -61.983 1.00 77.88 C

ATOM 8042 NE2 HIS D 81 27.374 16.323 -60.686 1.00 77.88 N

ATOM 8043 CD2 HIS D 81 27.691 15.399 -61.666 1.00 77.88 C

ATOM 8044 CE1 HIS D 81 28.209 16.058 -59.698 1.00 77.88 C

ATOM 8045 C HIS D 81 27.608 11.824 -62.026 1.00 77.88 C

ATOM 8046 O HIS D 81 26.705 11.671 -61.202 1.00 77.88 O

ATOM 8047 N ALA D 82 27.375 11.804 -63.352 1.00 46.36 N

ATOM 8048 CA ALA D 82 26.057 11.726 -63.921 1.00 46.36 C

ATOM 8049 CB ALA D 82 26.070 11.924 -65.447 1.00 46.36 C

ATOM 8050 C ALA D 82 25.397 10.416 -63.647 1.00 46.36 C

ATOM 8051 O ALA D 82 24.210 10.370 -63.333 1.00 46.36 O

ATOM 8052 N ALA D 83 26.144 9.307 -63.776 1.00 34.49 N

ATOM 8053 CA ALA D 83 25.555 8.014 -63.588 1.00 34.49 C

ATOM 8054 CB ALA D 83 26.540 6.864 -63.859 1.00 34.49 C

ATOM 8055 C ALA D 83 25.096 7.903 -62.171 1.00 34.49 C

ATOM 8056 O ALA D 83 24.030 7.360 -61.888 1.00 34.49 O

ATOM 8057 N HIS D 84 25.899 8.441 -61.238 1.00 61.17 N

ATOM 8058 CA HIS D 84 25.597 8.376 -59.839 1.00 61.17 C

ATOM 8059 ND1 HIS D 84 26.742 8.127 -56.674 1.00 61.17 N

ATOM 8060 CG HIS D 84 26.403 9.147 -57.533 1.00 61.17 C

ATOM 8061 CB HIS D 84 26.653 9.135 -59.012 1.00 61.17 C

ATOM 8062 NE2 HIS D 84 25.811 9.720 -55.432 1.00 61.17 N

ATOM 8063 CD2 HIS D 84 25.835 10.112 -56.759 1.00 61.17 C

ATOM 8064 CE1 HIS D 84 26.366 8.521 -55.431 1.00 61.17 C

ATOM 8065 C HIS D 84 24.267 9.029 -59.604 1.00 61.17 C

ATOM 8066 O HIS D 84 23.421 8.488 -58.893 1.00 61.17 O

ATOM 8067 N ARG D 85 24.042 10.217 -60.196 1.00112.78 N

ATOM 8068 CA ARG D 85 22.797 10.908 -59.991 1.00112.78 C

ATOM 8069 CB ARG D 85 22.774 12.363 -60.498 1.00112.78 C

ATOM 8070 CG ARG D 85 23.387 13.370 -59.516 1.00112.78 C

ATOM 8071 CD ARG D 85 23.265 14.819 -59.995 1.00112.78 C

ATOM 8072 NE ARG D 85 23.652 15.723 -58.874 1.00112.78 N

ATOM 8073 CZ ARG D 85 24.133 16.972 -59.146 1.00112.78 C

ATOM 8074 NH1 ARG D 85 24.344 17.359 -60.437 1.00112.78 N

ATOM 8075 NH2 ARG D 85 24.406 17.836 -58.125 1.00112.78 N

ATOM 8076 C ARG D 85 21.643 10.173 -60.607 1.00112.78 C

ATOM 8077 O ARG D 85 20.553 10.138 -60.040 1.00112.78 O

ATOM 8078 N PHE D 86 21.845 9.566 -61.789 1.00 96.69 N

ATOM 8079 CA PHE D 86 20.804 8.854 -62.480 1.00 96.69 C

ATOM 8080 CB PHE D 86 21.322 8.275 -63.814 1.00 96.69 C

ATOM 8081 CG PHE D 86 20.256 7.501 -64.515 1.00 96.69 C

ATOM 8082 CD1 PHE D 86 19.296 8.132 -65.272 1.00 96.69 C

ATOM 8083 CD2 PHE D 86 20.235 6.127 -64.429 1.00 96.69 C

ATOM 8084 CE1 PHE D 86 18.326 7.404 -65.921 1.00 96.69 C

ATOM 8085 CE2 PHE D 86 19.268 5.394 -65.076 1.00 96.69 C

ATOM 8086 CZ PHE D 86 18.310 6.033 -65.823 1.00 96.69 C

ATOM 8087 C PHE D 86 20.379 7.714 -61.616 1.00 96.69 C

ATOM 8088 O PHE D 86 19.191 7.408 -61.504 1.00 96.69 O

ATOM 8089 N ARG D 87 21.352 7.050 -60.971 1.00 47.43 N

ATOM 8090 CA ARG D 87 21.009 5.912 -60.180 1.00 47.43 C

ATOM 8091 CB ARG D 87 22.220 5.291 -59.468 1.00 47.43 C

ATOM 8092 CG ARG D 87 21.886 4.001 -58.719 1.00 47.43 C

ATOM 8093 CD ARG D 87 23.038 3.488 -57.855 1.00 47.43 C

ATOM 8094 NE ARG D 87 22.596 2.212 -57.228 1.00 47.43 N

ATOM 8095 CZ ARG D 87 21.873 2.224 -56.070 1.00 47.43 C

ATOM 8096 NH1 ARG D 87 21.512 3.409 -55.496 1.00 47.43 N

ATOM 8097 NH2 ARG D 87 21.508 1.044 -55.489 1.00 47.43 N

ATOM 8098 C ARG D 87 20.049 6.347 -59.121 1.00 47.43 C

ATOM 8099 O ARG D 87 18.977 5.765 -58.978 1.00 47.43 O

ATOM 8100 N PHE D 88 20.380 7.425 -58.390 1.00101.43 N

ATOM 8101 CA PHE D 88 19.545 7.875 -57.312 1.00101.43 C

ATOM 8102 CB PHE D 88 20.121 9.062 -56.519 1.00101.43 C

ATOM 8103 CG PHE D 88 19.077 9.441 -55.522 1.00101.43 C

ATOM 8104 CD1 PHE D 88 18.953 8.753 -54.336 1.00101.43 C

ATOM 8105 CD2 PHE D 88 18.215 10.485 -55.774 1.00101.43 C

ATOM 8106 CE1 PHE D 88 17.987 9.100 -53.420 1.00101.43 C

ATOM 8107 CE2 PHE D 88 17.248 10.837 -54.861 1.00101.43 C

ATOM 8108 CZ PHE D 88 17.132 10.143 -53.681 1.00101.43 C

ATOM 8109 C PHE D 88 18.229 8.331 -57.844 1.00101.43 C

ATOM 8110 O PHE D 88 17.197 8.149 -57.202 1.00101.43 O

ATOM 8111 N VAL D 89 18.234 8.987 -59.015 1.00 50.52 N

ATOM 8112 CA VAL D 89 17.004 9.535 -59.497 1.00 50.52 C

ATOM 8113 CB VAL D 89 17.176 10.315 -60.767 1.00 50.52 C

ATOM 8114 CG1 VAL D 89 15.790 10.775 -61.253 1.00 50.52 C

ATOM 8115 CG2 VAL D 89 18.159 11.467 -60.502 1.00 50.52 C

ATOM 8116 C VAL D 89 15.970 8.484 -59.778 1.00 50.52 C

ATOM 8117 O VAL D 89 14.874 8.537 -59.222 1.00 50.52 O

ATOM 8118 N LEU D 90 16.285 7.498 -60.643 1.00135.44 N

ATOM 8119 CA LEU D 90 15.239 6.618 -61.087 1.00135.44 C

ATOM 8120 CB LEU D 90 15.642 5.760 -62.297 1.00135.44 C

ATOM 8121 CG LEU D 90 15.714 6.554 -63.613 1.00135.44 C

ATOM 8122 CD1 LEU D 90 14.311 6.972 -64.087 1.00135.44 C

ATOM 8123 CD2 LEU D 90 16.677 7.745 -63.489 1.00135.44 C

ATOM 8124 C LEU D 90 14.684 5.689 -60.088 1.00135.44 C

ATOM 8125 O LEU D 90 13.505 5.778 -59.747 1.00135.44 O

ATOM 8126 N ASP D 91 15.513 4.772 -59.575 1.00 95.76 N

ATOM 8127 CA ASP D 91 14.863 3.830 -58.733 1.00 95.76 C

ATOM 8128 CB ASP D 91 15.717 2.602 -58.358 1.00 95.76 C

ATOM 8129 CG ASP D 91 16.902 3.017 -57.502 1.00 95.76 C

ATOM 8130 OD1 ASP D 91 17.513 4.077 -57.796 1.00 95.76 O

ATOM 8131 OD2 ASP D 91 17.212 2.274 -56.534 1.00 95.76 O

ATOM 8132 C ASP D 91 14.464 4.540 -57.501 1.00 95.76 C

ATOM 8133 O ASP D 91 13.345 4.387 -57.016 1.00 95.76 O

ATOM 8134 N HIS D 92 15.385 5.348 -56.957 1.00 99.75 N

ATOM 8135 CA HIS D 92 15.049 5.912 -55.695 1.00 99.75 C

ATOM 8136 ND1 HIS D 92 15.385 7.665 -52.879 1.00 99.75 N

ATOM 8137 CG HIS D 92 16.049 6.619 -53.481 1.00 99.75 C

ATOM 8138 CB HIS D 92 16.276 6.478 -54.958 1.00 99.75 C

ATOM 8139 NE2 HIS D 92 16.009 6.288 -51.248 1.00 99.75 N

ATOM 8140 CD2 HIS D 92 16.423 5.788 -52.469 1.00 99.75 C

ATOM 8141 CE1 HIS D 92 15.390 7.416 -51.545 1.00 99.75 C

ATOM 8142 C HIS D 92 14.009 6.998 -55.737 1.00 99.75 C

ATOM 8143 O HIS D 92 12.879 6.797 -55.299 1.00 99.75 O

ATOM 8144 N GLY D 93 14.351 8.163 -56.331 1.00 44.06 N

ATOM 8145 CA GLY D 93 13.499 9.324 -56.257 1.00 44.06 C

ATOM 8146 C GLY D 93 12.219 9.102 -56.987 1.00 44.06 C

ATOM 8147 O GLY D 93 11.140 9.419 -56.487 1.00 44.06 O

ATOM 8148 N LEU D 94 12.324 8.569 -58.215 1.00 66.51 N

ATOM 8149 CA LEU D 94 11.177 8.274 -59.020 1.00 66.51 C

ATOM 8150 CB LEU D 94 11.512 7.891 -60.473 1.00 66.51 C

ATOM 8151 CG LEU D 94 12.072 9.069 -61.297 1.00 66.51 C

ATOM 8152 CD1 LEU D 94 12.289 8.668 -62.765 1.00 66.51 C

ATOM 8153 CD2 LEU D 94 11.201 10.330 -61.142 1.00 66.51 C

ATOM 8154 C LEU D 94 10.482 7.134 -58.352 1.00 66.51 C

ATOM 8155 O LEU D 94 9.282 6.924 -58.511 1.00 66.51 O

ATOM 8156 N GLN D 95 11.257 6.346 -57.592 1.00113.39 N

ATOM 8157 CA GLN D 95 10.749 5.232 -56.855 1.00113.39 C

ATOM 8158 CB GLN D 95 9.651 5.621 -55.850 1.00113.39 C

ATOM 8159 CG GLN D 95 9.301 4.498 -54.873 1.00113.39 C

ATOM 8160 CD GLN D 95 8.413 5.084 -53.785 1.00113.39 C

ATOM 8161 OE1 GLN D 95 8.283 4.518 -52.701 1.00113.39 O

ATOM 8162 NE2 GLN D 95 7.792 6.258 -54.077 1.00113.39 N

ATOM 8163 C GLN D 95 10.237 4.187 -57.771 1.00113.39 C

ATOM 8164 O GLN D 95 9.328 3.432 -57.430 1.00113.39 O

ATOM 8165 N LEU D 96 10.824 4.100 -58.972 1.00110.32 N

ATOM 8166 CA LEU D 96 10.422 3.021 -59.805 1.00110.32 C

ATOM 8167 CB LEU D 96 10.109 3.444 -61.252 1.00110.32 C

ATOM 8168 CG LEU D 96 9.393 2.373 -62.104 1.00110.32 C

ATOM 8169 CD1 LEU D 96 9.150 2.885 -63.531 1.00110.32 C

ATOM 8170 CD2 LEU D 96 10.098 1.008 -62.080 1.00110.32 C

ATOM 8171 C LEU D 96 11.610 2.137 -59.822 1.00110.32 C

ATOM 8172 O LEU D 96 12.730 2.602 -60.034 1.00110.32 O

ATOM 8173 N GLY D 97 11.418 0.833 -59.560 1.00 78.40 N

ATOM 8174 CA GLY D 97 12.559 -0.014 -59.695 1.00 78.40 C

ATOM 8175 C GLY D 97 12.590 -0.271 -61.156 1.00 78.40 C

ATOM 8176 O GLY D 97 12.348 -1.384 -61.622 1.00 78.40 O

ATOM 8177 N ARG D 98 12.911 0.791 -61.914 1.00229.22 N

ATOM 8178 CA ARG D 98 12.858 0.727 -63.330 1.00229.22 C

ATOM 8179 CB ARG D 98 13.250 2.050 -64.009 1.00229.22 C

ATOM 8180 CG ARG D 98 13.260 1.994 -65.542 1.00229.22 C

ATOM 8181 CD ARG D 98 11.876 1.947 -66.196 1.00229.22 C

ATOM 8182 NE ARG D 98 11.265 0.620 -65.905 1.00229.22 N

ATOM 8183 CZ ARG D 98 10.673 -0.087 -66.912 1.00229.22 C

ATOM 8184 NH1 ARG D 98 10.699 0.395 -68.189 1.00229.22 N

ATOM 8185 NH2 ARG D 98 10.056 -1.275 -66.644 1.00229.22 N

ATOM 8186 C ARG D 98 13.836 -0.295 -63.776 1.00229.22 C

ATOM 8187 O ARG D 98 13.520 -1.147 -64.604 1.00229.22 O

ATOM 8188 N PHE D 99 15.056 -0.260 -63.214 1.00119.80 N

ATOM 8189 CA PHE D 99 16.001 -1.206 -63.707 1.00119.80 C

ATOM 8190 CB PHE D 99 17.305 -0.570 -64.219 1.00119.80 C

ATOM 8191 CG PHE D 99 16.966 0.197 -65.453 1.00119.80 C

ATOM 8192 CD1 PHE D 99 16.501 1.489 -65.366 1.00119.80 C

ATOM 8193 CD2 PHE D 99 17.107 -0.375 -66.697 1.00119.80 C

ATOM 8194 CE1 PHE D 99 16.187 2.201 -66.499 1.00119.80 C

ATOM 8195 CE2 PHE D 99 16.794 0.332 -67.835 1.00119.80 C

ATOM 8196 CZ PHE D 99 16.333 1.623 -67.737 1.00119.80 C

ATOM 8197 C PHE D 99 16.353 -2.171 -62.633 1.00119.80 C

ATOM 8198 O PHE D 99 16.809 -1.800 -61.552 1.00119.80 O

ATOM 8199 N ASP D 100 16.136 -3.462 -62.938 1.00120.04 N

ATOM 8200 CA ASP D 100 16.463 -4.530 -62.050 1.00120.04 C

ATOM 8201 CB ASP D 100 15.573 -5.773 -62.219 1.00120.04 C

ATOM 8202 CG ASP D 100 15.801 -6.339 -63.615 1.00120.04 C

ATOM 8203 OD1 ASP D 100 16.109 -5.539 -64.538 1.00120.04 O

ATOM 8204 OD2 ASP D 100 15.676 -7.583 -63.775 1.00120.04 O

ATOM 8205 C ASP D 100 17.855 -4.929 -62.389 1.00120.04 C

ATOM 8206 O ASP D 100 18.594 -4.166 -63.011 1.00120.04 O

ATOM 8207 N ARG D 101 18.239 -6.150 -61.973 1.00235.84 N

ATOM 8208 CA ARG D 101 19.564 -6.629 -62.222 1.00235.84 C

ATOM 8209 CB ARG D 101 19.868 -6.833 -63.720 1.00235.84 C

ATOM 8210 CG ARG D 101 21.109 -7.690 -63.990 1.00235.84 C

ATOM 8211 CD ARG D 101 21.370 -7.956 -65.476 1.00235.84 C

ATOM 8212 NE ARG D 101 22.409 -9.022 -65.566 1.00235.84 N

ATOM 8213 CZ ARG D 101 23.734 -8.697 -65.603 1.00235.84 C

ATOM 8214 NH1 ARG D 101 24.117 -7.388 -65.557 1.00235.84 N

ATOM 8215 NH2 ARG D 101 24.675 -9.682 -65.689 1.00235.84 N

ATOM 8216 C ARG D 101 20.485 -5.606 -61.659 1.00235.84 C

ATOM 8217 O ARG D 101 21.442 -5.181 -62.306 1.00235.84 O

ATOM 8218 N VAL D 102 20.198 -5.210 -60.404 1.00128.33 N

ATOM 8219 CA VAL D 102 20.915 -4.206 -59.683 1.00128.33 C

ATOM 8220 CB VAL D 102 22.209 -4.609 -59.062 1.00128.33 C

ATOM 8221 CG1 VAL D 102 22.765 -3.353 -58.369 1.00128.33 C

ATOM 8222 CG2 VAL D 102 21.965 -5.761 -58.087 1.00128.33 C

ATOM 8223 C VAL D 102 21.209 -3.031 -60.535 1.00128.33 C

ATOM 8224 O VAL D 102 22.192 -2.994 -61.273 1.00128.33 O

ATOM 8225 N ILE D 103 20.329 -2.025 -60.429 1.00 96.36 N

ATOM 8226 CA ILE D 103 20.520 -0.807 -61.139 1.00 96.36 C

ATOM 8227 CB ILE D 103 19.509 0.249 -60.791 1.00 96.36 C

ATOM 8228 CG1 ILE D 103 19.575 1.411 -61.797 1.00 96.36 C

ATOM 8229 CG2 ILE D 103 19.732 0.665 -59.327 1.00 96.36 C

ATOM 8230 CD1 ILE D 103 18.375 2.355 -61.721 1.00 96.36 C

ATOM 8231 C ILE D 103 21.870 -0.330 -60.717 1.00 96.36 C

ATOM 8232 O ILE D 103 22.584 0.304 -61.491 1.00 96.36 O

ATOM 8233 N ALA D 104 22.256 -0.631 -59.462 1.00 41.07 N

ATOM 8234 CA ALA D 104 23.543 -0.214 -58.985 1.00 41.07 C

ATOM 8235 CB ALA D 104 23.842 -0.692 -57.554 1.00 41.07 C

ATOM 8236 C ALA D 104 24.587 -0.801 -59.887 1.00 41.07 C

ATOM 8237 O ALA D 104 25.546 -0.121 -60.246 1.00 41.07 O

ATOM 8238 N LEU D 105 24.448 -2.083 -60.278 1.00 93.32 N

ATOM 8239 CA LEU D 105 25.408 -2.655 -61.183 1.00 93.32 C

ATOM 8240 CB LEU D 105 25.267 -4.172 -61.413 1.00 93.32 C

ATOM 8241 CG LEU D 105 25.889 -5.062 -60.315 1.00 93.32 C

ATOM 8242 CD1 LEU D 105 25.218 -4.876 -58.948 1.00 93.32 C

ATOM 8243 CD2 LEU D 105 25.924 -6.532 -60.764 1.00 93.32 C

ATOM 8244 C LEU D 105 25.308 -2.008 -62.527 1.00 93.32 C

ATOM 8245 O LEU D 105 26.323 -1.779 -63.182 1.00 93.32 O

ATOM 8246 N TRP D 106 24.078 -1.711 -62.983 1.00 98.43 N

ATOM 8247 CA TRP D 106 23.904 -1.193 -64.310 1.00 98.43 C

ATOM 8248 CB TRP D 106 22.423 -0.920 -64.626 1.00 98.43 C

ATOM 8249 CG TRP D 106 22.139 -0.446 -66.032 1.00 98.43 C

ATOM 8250 CD2 TRP D 106 21.660 -1.303 -67.080 1.00 98.43 C

ATOM 8251 CD1 TRP D 106 22.218 0.808 -66.564 1.00 98.43 C

ATOM 8252 NE1 TRP D 106 21.824 0.785 -67.882 1.00 98.43 N

ATOM 8253 CE2 TRP D 106 21.474 -0.507 -68.211 1.00 98.43 C

ATOM 8254 CE3 TRP D 106 21.387 -2.641 -67.095 1.00 98.43 C

ATOM 8255 CZ2 TRP D 106 21.013 -1.042 -69.380 1.00 98.43 C

ATOM 8256 CZ3 TRP D 106 20.930 -3.178 -68.277 1.00 98.43 C

ATOM 8257 CH2 TRP D 106 20.746 -2.393 -69.397 1.00 98.43 C

ATOM 8258 C TRP D 106 24.620 0.120 -64.465 1.00 98.43 C

ATOM 8259 O TRP D 106 25.503 0.261 -65.310 1.00 98.43 O

ATOM 8260 N CYS D 107 24.272 1.111 -63.621 1.00 89.44 N

ATOM 8261 CA CYS D 107 24.797 2.443 -63.753 1.00 89.44 C

ATOM 8262 CB CYS D 107 24.137 3.435 -62.778 1.00 89.44 C

ATOM 8263 SG CYS D 107 24.359 2.970 -61.034 1.00 89.44 S

ATOM 8264 C CYS D 107 26.276 2.489 -63.529 1.00 89.44 C

ATOM 8265 O CYS D 107 27.006 3.114 -64.297 1.00 89.44 O

ATOM 8266 N TYR D 108 26.761 1.851 -62.451 1.00122.87 N

ATOM 8267 CA TYR D 108 28.164 1.886 -62.163 1.00122.87 C

ATOM 8268 CB TYR D 108 28.509 1.478 -60.720 1.00122.87 C

ATOM 8269 CG TYR D 108 27.893 2.533 -59.856 1.00122.87 C

ATOM 8270 CD1 TYR D 108 28.469 3.780 -59.762 1.00122.87 C

ATOM 8271 CD2 TYR D 108 26.720 2.300 -59.174 1.00122.87 C

ATOM 8272 CE1 TYR D 108 27.908 4.765 -58.980 1.00122.87 C

ATOM 8273 CE2 TYR D 108 26.155 3.279 -58.388 1.00122.87 C

ATOM 8274 CZ TYR D 108 26.750 4.514 -58.286 1.00122.87 C

ATOM 8275 OH TYR D 108 26.173 5.519 -57.481 1.00122.87 O

ATOM 8276 C TYR D 108 28.912 1.050 -63.155 1.00122.87 C

ATOM 8277 O TYR D 108 30.045 1.367 -63.515 1.00122.87 O

ATOM 8278 N GLY D 109 28.316 -0.073 -63.596 1.00 19.69 N

ATOM 8279 CA GLY D 109 28.975 -0.922 -64.549 1.00 19.69 C

ATOM 8280 C GLY D 109 29.148 -0.173 -65.836 1.00 19.69 C

ATOM 8281 O GLY D 109 30.186 -0.253 -66.492 1.00 19.69 O

ATOM 8282 N MET D 110 28.121 0.591 -66.241 1.00 56.07 N

ATOM 8283 CA MET D 110 28.196 1.311 -67.477 1.00 56.07 C

ATOM 8284 CB MET D 110 26.917 2.110 -67.799 1.00 56.07 C

ATOM 8285 CG MET D 110 27.015 2.879 -69.119 1.00 56.07 C

ATOM 8286 SD MET D 110 25.544 3.846 -69.569 1.00 56.07 S

ATOM 8287 CE MET D 110 26.290 4.506 -71.087 1.00 56.07 C

ATOM 8288 C MET D 110 29.326 2.277 -67.360 1.00 56.07 C

ATOM 8289 O MET D 110 30.074 2.499 -68.311 1.00 56.07 O

ATOM 8290 N ALA D 111 29.483 2.882 -66.172 1.00 29.88 N

ATOM 8291 CA ALA D 111 30.529 3.843 -65.988 1.00 29.88 C

ATOM 8292 CB ALA D 111 30.523 4.472 -64.584 1.00 29.88 C

ATOM 8293 C ALA D 111 31.856 3.175 -66.178 1.00 29.88 C

ATOM 8294 O ALA D 111 32.727 3.699 -66.870 1.00 29.88 O

ATOM 8295 N VAL D 112 32.043 1.985 -65.579 1.00 93.11 N

ATOM 8296 CA VAL D 112 33.314 1.331 -65.673 1.00 93.11 C

ATOM 8297 CB VAL D 112 33.443 0.132 -64.775 1.00 93.11 C

ATOM 8298 CG1 VAL D 112 33.216 0.597 -63.326 1.00 93.11 C

ATOM 8299 CG2 VAL D 112 32.505 -0.984 -65.253 1.00 93.11 C

ATOM 8300 C VAL D 112 33.553 0.910 -67.088 1.00 93.11 C

ATOM 8301 O VAL D 112 34.670 1.013 -67.591 1.00 93.11 O

ATOM 8302 N LEU D 113 32.501 0.420 -67.770 1.00 85.08 N

ATOM 8303 CA LEU D 113 32.652 -0.050 -69.119 1.00 85.08 C

ATOM 8304 CB LEU D 113 31.329 -0.640 -69.661 1.00 85.08 C

ATOM 8305 CG LEU D 113 31.356 -1.298 -71.063 1.00 85.08 C

ATOM 8306 CD1 LEU D 113 29.980 -1.902 -71.386 1.00 85.08 C

ATOM 8307 CD2 LEU D 113 31.812 -0.350 -72.186 1.00 85.08 C

ATOM 8308 C LEU D 113 33.066 1.108 -69.973 1.00 85.08 C

ATOM 8309 O LEU D 113 34.022 1.018 -70.743 1.00 85.08 O

ATOM 8310 N GLY D 114 32.365 2.245 -69.835 1.00 24.44 N

ATOM 8311 CA GLY D 114 32.642 3.399 -70.638 1.00 24.44 C

ATOM 8312 C GLY D 114 34.020 3.883 -70.333 1.00 24.44 C

ATOM 8313 O GLY D 114 34.720 4.376 -71.214 1.00 24.44 O

ATOM 8314 N SER D 115 34.434 3.795 -69.056 1.00 31.11 N

ATOM 8315 CA SER D 115 35.737 4.279 -68.709 1.00 31.11 C

ATOM 8316 CB SER D 115 36.026 4.219 -67.201 1.00 31.11 C

ATOM 8317 OG SER D 115 35.155 5.103 -66.510 1.00 31.11 O

ATOM 8318 C SER D 115 36.763 3.451 -69.418 1.00 31.11 C

ATOM 8319 O SER D 115 37.732 3.981 -69.959 1.00 31.11 O

ATOM 8320 N ALA D 116 36.570 2.119 -69.446 1.00 26.95 N

ATOM 8321 CA ALA D 116 37.534 1.265 -70.077 1.00 26.95 C

ATOM 8322 CB ALA D 116 37.177 -0.226 -69.951 1.00 26.95 C

ATOM 8323 C ALA D 116 37.593 1.602 -71.534 1.00 26.95 C

ATOM 8324 O ALA D 116 38.672 1.744 -72.108 1.00 26.95 O

ATOM 8325 N THR D 117 36.422 1.797 -72.165 1.00103.27 N

ATOM 8326 CA THR D 117 36.388 2.053 -73.574 1.00103.27 C

ATOM 8327 CB THR D 117 35.002 2.225 -74.124 1.00103.27 C

ATOM 8328 OG1 THR D 117 34.386 3.376 -73.568 1.00103.27 O

ATOM 8329 CG2 THR D 117 34.184 0.965 -73.793 1.00103.27 C

ATOM 8330 C THR D 117 37.152 3.306 -73.850 1.00103.27 C

ATOM 8331 O THR D 117 37.854 3.403 -74.855 1.00103.27 O

ATOM 8332 N ALA D 118 37.012 4.313 -72.972 1.00 31.82 N

ATOM 8333 CA ALA D 118 37.699 5.561 -73.140 1.00 31.82 C

ATOM 8334 CB ALA D 118 37.304 6.604 -72.078 1.00 31.82 C

ATOM 8335 C ALA D 118 39.172 5.342 -73.012 1.00 31.82 C

ATOM 8336 O ALA D 118 39.964 5.920 -73.755 1.00 31.82 O

ATOM 8337 N GLY D 119 39.576 4.491 -72.051 1.00 21.19 N

ATOM 8338 CA GLY D 119 40.967 4.260 -71.796 1.00 21.19 C

ATOM 8339 C GLY D 119 41.599 3.679 -73.018 1.00 21.19 C

ATOM 8340 O GLY D 119 42.722 4.040 -73.368 1.00 21.19 O

ATOM 8341 N TRP D 120 40.903 2.752 -73.703 1.00 91.41 N

ATOM 8342 CA TRP D 120 41.515 2.181 -74.865 1.00 91.41 C

ATOM 8343 CB TRP D 120 40.682 1.137 -75.642 1.00 91.41 C

ATOM 8344 CG TRP D 120 40.368 -0.181 -74.969 1.00 91.41 C

ATOM 8345 CD2 TRP D 120 39.030 -0.655 -74.749 1.00 91.41 C

ATOM 8346 CD1 TRP D 120 41.199 -1.192 -74.584 1.00 91.41 C

ATOM 8347 NE1 TRP D 120 40.463 -2.255 -74.113 1.00 91.41 N

ATOM 8348 CE2 TRP D 120 39.125 -1.940 -74.219 1.00 91.41 C

ATOM 8349 CE3 TRP D 120 37.822 -0.078 -75.000 1.00 91.41 C

ATOM 8350 CZ2 TRP D 120 38.004 -2.665 -73.924 1.00 91.41 C

ATOM 8351 CZ3 TRP D 120 36.693 -0.796 -74.675 1.00 91.41 C

ATOM 8352 CH2 TRP D 120 36.783 -2.067 -74.146 1.00 91.41 C

ATOM 8353 C TRP D 120 41.716 3.287 -75.845 1.00 91.41 C

ATOM 8354 O TRP D 120 42.756 3.383 -76.492 1.00 91.41 O

ATOM 8355 N MET D 121 40.710 4.170 -75.956 1.00113.26 N

ATOM 8356 CA MET D 121 40.699 5.188 -76.964 1.00113.26 C

ATOM 8357 CB MET D 121 39.394 5.986 -76.958 1.00113.26 C

ATOM 8358 CG MET D 121 38.171 5.093 -77.163 1.00113.26 C

ATOM 8359 SD MET D 121 38.365 3.881 -78.504 1.00113.26 S

ATOM 8360 CE MET D 121 38.808 5.088 -79.786 1.00113.26 C

ATOM 8361 C MET D 121 41.859 6.132 -76.812 1.00113.26 C

ATOM 8362 O MET D 121 42.462 6.531 -77.807 1.00113.26 O

ATOM 8363 N LEU D 122 42.187 6.524 -75.568 1.00121.91 N

ATOM 8364 CA LEU D 122 43.262 7.433 -75.251 1.00121.91 C

ATOM 8365 CB LEU D 122 43.218 7.795 -73.745 1.00121.91 C

ATOM 8366 CG LEU D 122 44.272 8.771 -73.167 1.00121.91 C

ATOM 8367 CD1 LEU D 122 43.964 9.040 -71.686 1.00121.91 C

ATOM 8368 CD2 LEU D 122 45.726 8.291 -73.328 1.00121.91 C

ATOM 8369 C LEU D 122 44.573 6.785 -75.558 1.00121.91 C

ATOM 8370 O LEU D 122 45.526 7.460 -75.944 1.00121.91 O

ATOM 8371 N LEU D 123 44.665 5.463 -75.318 1.00 78.26 N

ATOM 8372 CA LEU D 123 45.849 4.697 -75.573 1.00 78.26 C

ATOM 8373 CB LEU D 123 45.714 3.229 -75.133 1.00 78.26 C

ATOM 8374 CG LEU D 123 45.450 3.077 -73.623 1.00 78.26 C

ATOM 8375 CD1 LEU D 123 45.390 1.598 -73.208 1.00 78.26 C

ATOM 8376 CD2 LEU D 123 46.450 3.897 -72.793 1.00 78.26 C

ATOM 8377 C LEU D 123 46.032 4.723 -77.046 1.00 78.26 C

ATOM 8378 O LEU D 123 47.149 4.570 -77.538 1.00 78.26 O

ATOM 8379 N THR D 124 44.887 4.783 -77.757 1.00164.27 N

ATOM 8380 CA THR D 124 44.761 5.010 -79.166 1.00164.27 C

ATOM 8381 CB THR D 124 46.035 5.341 -79.895 1.00164.27 C

ATOM 8382 OG1 THR D 124 46.676 6.454 -79.290 1.00164.27 O

ATOM 8383 CG2 THR D 124 45.674 5.711 -81.336 1.00164.27 C

ATOM 8384 C THR D 124 44.161 3.802 -79.794 1.00164.27 C

ATOM 8385 O THR D 124 44.471 2.669 -79.430 1.00164.27 O

ATOM 8386 N MET D 125 43.276 4.036 -80.776 1.00 97.45 N

ATOM 8387 CA MET D 125 42.655 2.971 -81.499 1.00 97.45 C

ATOM 8388 CB MET D 125 41.121 3.113 -81.575 1.00 97.45 C

ATOM 8389 CG MET D 125 40.411 1.969 -82.305 1.00 97.45 C

ATOM 8390 SD MET D 125 40.422 0.376 -81.431 1.00 97.45 S

ATOM 8391 CE MET D 125 39.659 -0.548 -82.795 1.00 97.45 C

ATOM 8392 C MET D 125 43.217 3.097 -82.912 1.00 97.45 C

ATOM 8393 O MET D 125 44.395 3.527 -83.027 1.00 97.45 O

ATOM 8394 OXT MET D 125 42.489 2.780 -83.892 1.00 97.45 O

TER 8394 MET D 125

HETATM 1 C34 VIA 1000 19.312 10.914 -50.307 1.00 0.00 C

HETATM 2 C33 VIA 1000 20.089 11.372 -51.512 1.00 0.00 C

HETATM 3 C32 VIA 1000 19.845 12.887 -51.828 1.00 0.00 C

HETATM 4 C30 VIA 1000 20.697 13.115 -53.034 1.00 0.00 C

HETATM 5 N29 VIA 1000 20.663 12.337 -54.200 1.00 0.00 N

HETATM 6 N28 VIA 1000 21.567 12.835 -55.093 1.00 0.00 N

HETATM 7 C31 VIA 1000 21.781 12.246 -56.418 1.00 0.00 C

HETATM 8 C24 VIA 1000 22.169 13.918 -54.504 1.00 0.00 C

HETATM 9 C23 VIA 1000 23.181 14.844 -54.930 1.00 0.00 C

HETATM 10 O27 VIA 1000 23.713 14.789 -56.015 1.00 0.00 O

HETATM 11 N22 VIA 1000 23.573 15.852 -54.052 1.00 0.00 N

HETATM 12 C21 VIA 1000 23.033 16.022 -52.769 1.00 0.00 C

HETATM 13 N26 VIA 1000 22.041 15.148 -52.311 1.00 0.00 N

HETATM 14 C25 VIA 1000 21.631 14.110 -53.196 1.00 0.00 C

HETATM 15 C9 VIA 1000 23.542 17.096 -51.989 1.00 0.00 C

HETATM 16 C8 VIA 1000 24.974 17.093 -51.773 1.00 0.00 C

HETATM 17 C7 VIA 1000 25.624 18.149 -51.000 1.00 0.00 C

HETATM 18 C6 VIA 1000 24.752 19.230 -50.439 1.00 0.00 C

HETATM 19 C5 VIA 1000 23.308 19.252 -50.646 1.00 0.00 C

HETATM 20 C4 VIA 1000 22.663 18.184 -51.435 1.00 0.00 C

HETATM 21 O3 VIA 1000 21.244 18.131 -51.716 1.00 0.00 O

HETATM 22 C2 VIA 1000 20.238 18.917 -51.045 1.00 0.00 C

HETATM 23 C1 VIA 1000 18.938 18.577 -51.754 1.00 0.00 C

HETATM 24 S10 VIA 1000 27.210 18.167 -50.779 1.00 0.00 S

HETATM 25 O11 VIA 1000 27.689 19.183 -50.085 1.00 0.00 O

HETATM 26 O12 VIA 1000 27.566 17.097 -50.107 1.00 0.00 O

HETATM 27 N14 VIA 1000 27.713 18.142 -52.287 1.00 0.00 N

HETATM 28 C19 VIA 1000 28.422 16.803 -52.731 1.00 0.00 C

HETATM 29 C18 VIA 1000 29.916 16.646 -52.390 1.00 0.00 C

HETATM 30 N17 VIA 1000 30.754 17.888 -52.494 1.00 0.00 N

HETATM 31 C20 VIA 1000 31.735 17.700 -53.607 1.00 0.00 C

HETATM 32 C16 VIA 1000 30.064 19.245 -52.654 1.00 0.00 C

HETATM 33 C15 VIA 1000 28.511 19.266 -52.920 1.00 0.00 C

TER 34 VIA 1000

CONECT 1 2

CONECT 2 1 3

CONECT 3 2 4

CONECT 4 3 5 14

CONECT 5 4 6

CONECT 6 5 7 8

CONECT 7 6

CONECT 8 6 9 14

CONECT 9 8 10 11

CONECT 10 9

CONECT 11 9 12

CONECT 12 11 13 15

CONECT 13 12 14

CONECT 14 4 8 13

CONECT 15 12 16 20

CONECT 16 15 17

CONECT 17 16 18 24

CONECT 18 17 19

CONECT 19 18 20

CONECT 20 15 19 21

CONECT 21 20 22

CONECT 22 21 23

CONECT 23 22

CONECT 24 17 25 26 27

CONECT 25 24

CONECT 26 24

CONECT 27 24 28 33

CONECT 28 27 29

CONECT 29 28 30

CONECT 30 29 31 32

CONECT 31 30

CONECT 32 30 33

CONECT 33 27 32

END
